# Late-onset methylmalonic acidemia and homocysteinemia (cblC disease): systematic review

**DOI:** 10.1186/s13023-024-03021-3

**Published:** 2024-01-20

**Authors:** Loredana Arhip, Noemi Brox-Torrecilla, Inmaculada Romero, Marta Motilla, Clara Serrano-Moreno, María Miguélez, Cristina Cuerda

**Affiliations:** 1 Unidad de Nutrición Clínica y Dietética, Hospital General Universitario Gregorio Marañón, Calle del Doctor Esquerdo 46, 28007 Madrid, Spain; 2 Instituto de Investigación Sanitaria Gregorio Marañón, Madrid, Spain; 3 Hospital Universitario Ramón y Cajal, Madrid, Spain; 4 Hospital General Universitario Gregorio Marañón, Madrid, Spain; 5 Hospital Universitario Fundación Jiménez Díaz, Madrid, Spain; 6 Universidad Complutense Madrid, Madrid, Spain

**Keywords:** Methylmalonic acidemia and homocystinuria, cblC type, Late-onset, MMACHC protein

## Abstract

**Introduction:**

Combined methylmalonic acidemia and homocystinuria, cblC type is an inborn error of intracellular cobalamin metabolism and the most common one. The age of onset ranges from prenatal to adult. The disease is characterised by an elevation of methylmalonic acid (MMA) and homocysteine and a decreased production of methionine. The aim is to review existing scientific literature of all late onset cblC patients in terms of clinical symptoms, diagnosis, and outcome.

**Methods:**

A bibliographic database search was undertaken in PubMed (MEDLINE) complemented by a reference list search. We combined search terms regarding cblC disease and late onset. Two review authors performed the study selection, data extraction and quality assessment.

**Results:**

Of the sixty-five articles included in this systematic review, we collected a total of 199 patients. The most frequent clinical symptoms were neuropathy/myelopathy, encephalopathy, psychiatric symptoms, thrombotic microangiopathy, seizures, kidney disease, mild to severe pulmonary hypertension with heart failure and thrombotic phenomena. There were different forms of supplementation used in the different studies collected and, within these studies, some patients received several treatments sequentially and/or concomitantly. The general outcome was: 64 patients recovered, 78 patients improved, 4 patients did not improve, or the disease progressed, and 12 patients died.

**Conclusions:**

Most scientific literature regarding the late onset cblC disease comes from case reports and case series. In most cases treatment initiation led to an improvement and even recovery of some patients. The lack of complete recovery underlines the necessity for increased vigilance in unclear clinical symptoms for cblC disease.

**Supplementary Information:**

The online version contains supplementary material available at 10.1186/s13023-024-03021-3.

## Introduction

Combined methylmalonic acidemia and homocystinuria, cblC type (OMIM #277400) is an inborn error of intracellular cobalamin metabolism and the most common one [1]. The incidence of cblC disease has been estimated at 1:200,000 births [2].

The age of onset ranges from prenatal to adult. The disorder has been classified as early-onset, where the first presentation of disease is within the first year of life (or 4 years of age according to some authors) and late-onset, where the first symptoms appear later on. Although less frequent that the infantile presentation, the adolescent and adult presentation usually includes neurological and neuropsychiatric manifestations including neuropsychiatric symptoms, progressive cognitive decline, brain MRI may reveal leukodystrophy ranging from isolated periventricular white matter hyperintensities to diffuse white matter loss, subacute combined degeneration of the spinal cord, renal thrombotic microangiopathy, haemolytic uremic syndrome (HUS), kidney disease, pulmonary hypertension, and pulmonary thrombotic events [1].

The disease is characterized by a decreased intracellular production of adenosylcobalamin (AdoCbl) and methylcobalamin (MeCbl), cofactors for the methylmalonyl-CoA mutase and methionine synthase enzymes. The deficient activity of these enzymes causes an elevation of methylmalonic acid (MMA) and homocysteine and a decreased production of methionine [1]. Plasmas vitamin B12 levels are normal.

CblC disease is an autosomal recessive disorder caused by mutations in the MMACHC gene (OMIM *609831) located on chromosome 1p34 [3]. There are more than 50 known mutations identified in the MMACHC gene that cause cblC disease [1].

The suggested therapeutic interventions in cblC disease include hydroxocobalamin (intramuscular, subcutaneous, or intravenous), whose purpose is to reduce serum methylmalonic acid (MMA) and total homocysteine levels. Other relevant treatments are betaine, folinic acid and levocarnitine [1].

The diagnosis can be challenging because the clinical manifestations and age of presentation are highly variable. The importance of the early diagnosis is highlighted by the relentless progression of this multi-systemic disease in the absence of appropriate treatment [1]. As such, the aim of the current study is to review existing scientific literature of all late onset cblC patients in terms of clinical symptoms, diagnosis, and outcome.

## Methods

The current review conformed to the PRISMA statement for reporting systematic reviews and meta-analyses [4]. The PRISMA checklist of items included in this systematic review is available in Additional file 1: Table S1. The review was registered on the international database of prospectively registered systematic reviews (PROSPERO) on 8 February 2021 with the number CRD42021229995 [5].

### Publication search

We conducted a search using the database PubMed (MEDLINE) without any language or time limitations. We combined search terms regarding cblC disease and late onset. See Additional file 1: Table S2 for full search strategies.

### Study selection

The title, abstract and keywords of the articles were screened for relevance by two review authors (LA and NBT). Studies that did not provide the information relevant to this systematic review were excluded. Full articles were retrieved for further review after the initial screening and subjected to full text reading and implementation of inclusion/exclusion criteria (Table 1). Hand searching of papers was also performed.

**Table 1:** Inclusion and exclusion criteria

	Inclusion criteria	Exclusion criteria
Population	Patients older than 12 months at the time of diagnosis	Patients younger than 12 months at the time of diagnosis
Design	Any study design that includes clinical data of patients with late onset CblC disease	Any study design that does not include clinical data of patients with late onset CblC disease
Intervention	Clinical spectrum of the late onset cblC desease	
Comparator	Not applicable	
Outcome	Types of clinical presentation, modes of diagnosis, biochemical and clinical response to treatment, general outcome	
Country	All countries	
Method	Primary studies	Narrative reviews Expert opinions Editorials Qualitative studies
Publication type	Published in peer-reviewed databases	Conference papers Dissertations Books/books chapters Unpublished material Grey literature
Year of publications	Published in any year	
Language	English and Spanish	Other languages
Other	Humans	Animal studies

### Data extraction

Two review authors (LA and NBT) extracted the data. Disagreement was resolved through discussion or consulting a third review author (CC). Data were extracted in standardised tables. The data included was: lead author, year of publication, country, study title, main objective, type of study, funding, number of cases, age at onset, age at diagnosis, sex, ethnicity, family history, previous medical history, clinical presentation, biochemical diagnosis (MMA, homocysteine, methionine, C3 and C3/C2 ration, other studies (MRI, ECG, etc.,), cell type analysis for diagnosis, genetic diagnosis, treatment (cobalamin, folic acid, betaine, L-carnitine, vitamin B6, diet, other), biochemical and clinical response to treatment, general outcome and follow-up. When possible, mean, and standard deviation was calculated.

### Quality assessment

Two review authors (LA and NBT) performed the quality assessment. The CARE guidelines [6] were used to assess the case reports/case series. The heterogeneity of data between the studies included in this review prevented meta-analysis.

## Results

**Fig. 1:**
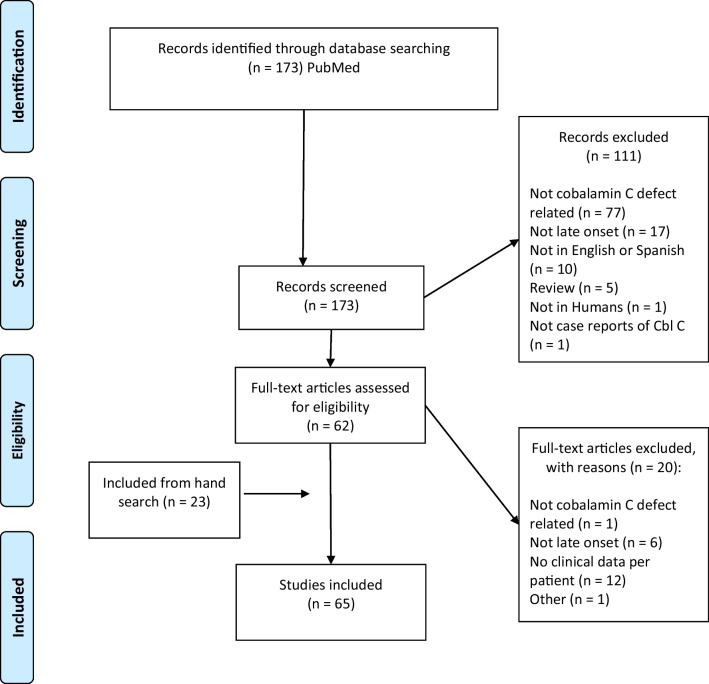
PRISMA 2009 Flow Diagram. From Moher D, Liberati A, Tetzlaff J, Altman DG, The PRISMA Group (2009). Preferred Reporting Items for Systematic Reviews and Meta-Analyses: The PRISMA Statement. PLoS Med 6(7): e1000097. https://doi.org/10.1371/journal.pmed1000097. For more information, visit www.prisma-statement.org

As shown in the PRISMA flow diagram (Fig. 1), 173 records were retrieved through database searching, with 111 articles excluded after screening of title/abstract (reasons listed in Fig. 1). This left 62 records for full text screening, of which 20 were excluded secondary to reasons listed in Fig. 1. Twenty-three additional papers were included through hand searching the references. A total of 65 papers were included in the final review [7–71].

### Quality of studies

Overall, 56.47% of the quality criteria were met for the case reports/case series. (Additional file 1: Table S3). Reasons for deductions resulted from failure to report the required keywords, items regarding patient information, the therapeutic intervention or follow-up data.

### Summary of the results

The following sections summarise the results of the included studies.

#### General characteristics of the studies included in the systematic review

A total of 65 articles were included in this systematic review. A summary of the main features of the sixty-five studies is presented in Additional file 1: Table S4.

#### Age at onset and diagnosis, patient history and clinical presentation

Of the sixty-five articles included in this systematic review, we collected a total of 199 patients, 94 were women, 92 were men and 13 patients whose gender was not recorded (Additional file 1: Table S5). The mean age at onset of symptoms was 15.29 ± 9.22 years. The mean age at diagnosis was 17.62 ± 10.82 years. 148 patients (74.3% of the sample) began their symptoms when they were > 4 years old and 19 patients (9.5% of the sample) with 4 years or less. Of 32 patients (16% of the sample) we do not have the specific age of onset.

Regarding family history, 27 patients had a history of non-consanguinity; on the contrary, 14 patients had a history of consanguinity in their parents. There were a total of 53 siblings in the included studies. Twenty-eight patients had uneventful family history. When the mutation was recorded in the parents of the patients, 41 were carriers of an inherited mutation. In 80 patients there was no family history recorded.

Among the personal history of the patients, the most frequent symptoms were symptoms of intellectual disability, learning difficulties and delayed learning, memory abilities and decreased locomotor activities during childhood that occurred in 29 patients. Other frequent personal histories were psychiatric disorders in 6 patients, anaemia in 6 patients, respiratory disorders in 6 patients, kidney disease in 5 patients, seizures in 5 patients, gait disorders in 4 patients, thrombotic microangiopathy in 4 patients and thrombotic episodes in 3 patients. Other history collected included urinary tract infections, headaches, hypertension, emesis, diarrhoea, asthenia, and weight loss. 25 patients had uneventful medical history. We don´t have data on the personal history of 126 patients.

Apropos of the clinical presentation of cobalamin C deficiency at the diagnosis, the most frequent symptoms were:

1. Neuropathy/myelopathy in 94 patients, manifested as atactic gait, numbness, weakness in both lower extremities, peripheral neuropathy, paraplegia of the legs, loss of bowel and bladder function subacute and combined degeneration of the spinal cord.
2. Encephalopathy in 80 patients with symptoms of cognitive impairment, learning difficulties, loss of learned skills, unwillingness to communicate with others, dementia, acute encephalopathy, including coma.
3. Psychiatric symptoms in 57 patients, the most frequent being behavioural changes, depression, bipolar disorder, paranoia, and visual and auditory hallucinations.
4. Thrombotic microangiopathy in 38 patients, manifested as acute renal failure, haemolytic anaemia, thrombocytopenia, and hypertension.
5. Seizures in 28 patients, tonic–clonic seizures being the most frequent.
6. Mild to severe pulmonary hypertension with heart failure in 20 patients.
7. Thrombotic phenomena in 15 patients, the most frequent being deep vein thrombosis and pulmonary thromboembolism
8. Megaloblastic anaemia was common in at least 13 patients.
9. In the paediatric age, common symptoms were developmental delay and failure to thrive.
10. Other less frequent symptoms in this pathology were anorexia or weight loss, ocular pathology, diarrhoea, pneumonia, liver failure, nausea, and vomiting.

It should be noted that 7 patients were asymptomatic and for 11 patients we do not have information on the clinical presentation.

### Biochemical diagnosis

At diagnosis, MMA levels, in urine and/or plasma, were measured in 178 patients (89.4% of the sample). Of these patients, 177 (99.4%) had elevated values. The values of 21 patients (10.55%) were not available (Additional file 1: Table S6).

Homocysteine levels, in plasma and/or urine, were measured in 181 patients (90.95% of the sample). All of the patients (100%) presented elevated values. Homocysteine value was not available for 18 of them.

Methionine levels were measured in 57 patients (28% of the sample), being decreased in 28 patients (49% of the total measurements).

C3 levels and C3/C2 ratio were measured in 41 patients (20.6% of the sample) and 63 patients (31.65% of the sample), respectively. Of these patients evaluated, elevated levels of C3 were found in 35 patients (85.36%) and elevated levels of C3/C2 ratio in 58 patients (92%).

Vitamin B12 and/or folic acid values were measured in 123 patients (61.8% of the sample). All had normal or high values, except for one patient in whom low folic acid values were observed.

### Other studies

Depending on the case, other studies were performed such as magnetic resonance imaging (cerebral and spinal), echocardiogram, EEG, renal ultrasound, etc. Additional file 1: Table S7 summarizes these complementary tests that were performed on each patient.

### Cell type analysis for diagnosis and genetic diagnosis

Measurement of AdoCbl and MetCbl synthesis in cultured skin fibroblasts was performed in 34 patients. Cultured Epstein–Barr virus (EBV)-infected lymphocytes confirmed defective MetCbl and AdoCbl synthesis in 2 patients. Sequencing analysis of the coding exons of the MMAHC gene was performed on most of the sample (183 patients) confirming the diagnosis of cobalamin C deficiency. 2 of these genetic tests were performed post-mortem (Additional file 1: Table S8).

### Cobalamin C disease treatment

There were different forms of supplementation used in the different studies collected and, within these studies; some patients received several treatments sequentially and/or concomitantly. 117 patients received treatment with Hydroxocobalamin, 30 intravenously and 75 intramuscularly. Of the remaining 12 patients, the mode of administration is not specified. 42 patients received intramuscular or intravenous cyanocobalamin. 5 patients were treated with oral vitamin B12 supplementation. 31 patients were treated with other molecules of vitamin B12 (methylcobalamin, cobalamin mixture, adenosylcobalamin). We do not have information on the treatment of 40 patients. Finally, 3 patients were not treated (Additional file 1: Table S9).

Regarding other therapeutic interventions, 127 patients received folic acid, 134 received betaine, 79 received L-carnitine and 15 vitamin B6. Also, 4 patients followed a low-protein diet and 3 a normal diet.

### Outcome and follow-up

Regarding the biochemical response after treatment, of the analysed sample, 53 patients decreased levels of homocysteine  ± MMA, and 21 patients had a complete response with normalization of these parameters. One patient presented variations of plasma homocysteine levels because of the changes in treatment. In another patient the elevated levels remained due to suboptimal compliance. No information was available for 123 patients (Additional file 1: Table S10). Thus, the general outcome of the patients was as follows: 64 patients recovered clinically and biochemically, 78 patients improved, 4 patients did not improve, and 12 patients died. Of 30 patients we have no information. It should be noted that 11 patients were asymptomatic. The mean follow-up was 31.87 ± 43.48 months.

## Discussion

The aim of the current study was to review existing scientific literature of all late onset CblC patients in terms of clinical symptoms, diagnosis, and outcome.

Of the sixty-five articles included in this systematic review, we collected a total of 199 patients. This is a much larger number of patients compared to those published in the literature. For instance, Huemer et al. reported the cases of 58 patients (and three new cases). The reason for this discrepancy might be that the article was published in 2014, it was not a systematic review and it included only case reports [72]. On the other side, in our paper, we performed a systematic review up to 2021 and we also included the data of patients reported in original research. Although this data might not be as complete as the one coming from case reports, the decision of including them has raised the number of patients with this disease, which might give us an idea of how many patients there actually are out there.

Regarding quality assessment, the CARE guidelines [6] that we used to assess the case reports/case series have been published in 2013, whereas many of the articles included were published before this year.

The numerous terminologies used by authors to refer to this pathology indicate the difficulty in retrieving fast and good results about this disease. This is especially relevant when specialists are confronting a difficult case that requires rapid literature research.

Genetic sequencing makes it possible to correctly diagnose these patients and to be able to locate asymptomatic carriers through the directed genetic study of relatives. None of these patients was diagnosed by neonatal screening (NBS). The most plausible explanation is the fact that it is a late-onset disease, and the first symptoms and metabolic alterations occur after 12 months of life. One possible way of study would be to find out if there is any metabolic alteration from birth in order to prevent the development of complications in these patients.

Most of the patients included received treatment with hydroxocobalamin intravenously or intramuscularly, although dosages were quite variable in practice.

Despite the hydroxocobalamin treatment, long-term outcome in patients with cblC disease is often unsatisfactory. While biochemical abnormalities, mortality, and non-neurological symptoms usually improve under medical treatment, long-term neurological and ophthalmological outcomes often remain uninfluenced [36].

Regarding dietary restrictions, the most recent guidelines strongly recommend not to restrict protein in cblC disease and other remethylation disorders (Quality of the evidence: moderate) [73].

It should be noted that although the diagnosis of these patients is made in the context of an acute manifestation of the disease (neurological or psychiatric disorders, thrombosis, HUS…), many of these patients already have a personal history that could lead to suspicion of the presence of this metabolic disease (such as neurological disorders, kidney failure…).

It is noteworthy that there are hardly any reported cases of ocular symptoms in patients with late-onset CblC; while in early-onset patients, maculopathy is one of the main manifestations. This can be explained because the changes occur during a critical stage of postnatal foveal development and visual maturation and raise the possibility of a particular vulnerability of central photoreceptors and/or ganglion cells to metabolic abnormality during this early period [74].

Since the onset of symptoms can vary widely, as a consequence, the first specialist seeing an adult patient affected by cblC disease can vary as well. It is therefore of great importance that all these specialists are aware of the disease and sensible to other signs and symptoms, not inherently belonging to their area, but whose presence could orientate towards the correct diagnosis.

For many decades, the inborn errors of metabolism (IEM) have been disorders predominantly managed by paediatricians. Nevertheless, nowadays, IEM represent a growing specialty in adult medicine. Reasons for this include improved diagnosis through expanded new-born screening programmes, identification of potentially affected family members and greater awareness of symptomatic presentations in adolescence and in adulthood. Greatly improved survival and reduced mortality from previously lethal and debilitating conditions have enabled survival into adulthood and reproductive age.

This requires physicians from other specialties to specialise in this area and keep up to date on the care of these patients. The physicians who might follow patients with different IEM vary, going from internal medicine, endocrinologists, nutritionists, dieticians, gastroenterologists and so on. Many of them work conjointly with clinical biochemists.

The learning that the authors have made when carrying out this systematic review deals with the severity of the clinical manifestations of this entity that can lead to the death of the patients, the heterogeneity in the clinical presentation that can delay the diagnosis, and so on. The main objective is that all of the professionals listed above whom might encounter this type of patients should be able to suspect and diagnose it correctly and thus be able to start a correct, easily available, and inexpensive treatment that prevents progression and can reverse some of the clinical manifestations of this entity.

Some of the limitations of our work include that, although we comprehensively examined the peer reviewed literature, there may be unpublished grey literature studies that were not included. We also only included studies published in English and Spanish. However, there may be studies in other languages that were not included. Also, given the different types of studies comprising this review (case reports, case series, original research and so on) the data could not be uniformly extracted and therefore some of the patients included were not extensively described. Nevertheless, the present systematic review contains several strengths. Firstly, it was performed in accordance with the PRISMA guidelines. Lastly, two review authors performed the study selection, data extraction and quality assessment.

## Conclusions

Most scientific literature regarding the late onset cblC disease comes from case reports and case series. In most cases treatment initiation led to an improvement and even recovery of some patients, the lack of complete recovery underlines the necessity for increased vigilance in unclear clinical symptoms for cblC disease.

### Supplementary Information

**Additional file 1**. Supplementary tables.

## Data Availability

All data relevant to the study are included in the article or uploaded as Additional file 1.

## Abbreviations

C3: Propionylcarnitine
C2: Acetylcarnitine
cblC: Cobalamin C disease
AdoCbl: Adenosylcobalamin
MeCbl: Methylcobalamin
MMA: Methylmalonic acid
HUS: Haemolytic uremic syndrome
MRI: Magnetic resonance imaging
ECG: Electrocardiogram
EBV: Epstein–Barr virus
