# Supplementary material for: Late-onset methylmalonic acidemia and homocysteinemia (cblC disease): systematic review
Source: Orphanet J Rare Dis. 2024 Jan 20;19:20. doi: 10.1186/s13023-024-03021-3 (PMC10799514; doi:10.1186/s13023-024-03021-3)
Supplement: Supplementary file 1 — Additional file 1. Supplementary tables. [file 13023_2024_3021_MOESM1_ESM.docx]

**Supplementary Material**

**Tables**

**Table 1. PRISMA checklist of items included in this systematic review.**

| **Section/topic** | **#** | **Checklist item** | **Reported on page #** |
| --- | --- | --- | --- |
| **TITLE** | | |  |
| Title | 1 | Identify the report as a systematic review, meta-analysis, or both. | 1 |
| **ABSTRACT** | | |  |
| Structured summary | 2 | Provide a structured summary including, as applicable: background; objectives; data sources; study eligibility criteria, participants, and interventions; study appraisal and synthesis methods; results; limitations; conclusions and implications of key findings; systematic review registration number. | 2 |
| **INTRODUCTION** | | |  |
| Rationale | 3 | Describe the rationale for the review in the context of what is already known. | 3 |
| Objectives | 4 | Provide an explicit statement of questions being addressed with reference to participants, interventions, comparisons, outcomes, and study design (PICOS). | 3 |
| **METHODS** | | |  |
| Protocol and registration | 5 | Indicate if a review protocol exists, if and where it can be accessed (e.g., Web address), and, if available, provide registration information including registration number. | 4 |
| Eligibility criteria | 6 | Specify study characteristics (e.g., PICOS, length of follow-up) and report characteristics (e.g., years considered, language, publication status) used as criteria for eligibility, giving rationale. | 4 and Table 1 |
| Information sources | 7 | Describe all information sources (e.g., databases with dates of coverage, contact with study authors to identify additional studies) in the search and date last searched. | 4 |
| Search | 8 | Present full electronic search strategy for at least one database, including any limits used, such that it could be repeated. | 4 and Supplementary Material, Table 2 |
| Study selection | 9 | State the process for selecting studies (i.e., screening, eligibility, included in systematic review, and, if applicable, included in the meta-analysis). | 4 |
| Data collection process | 10 | Describe method of data extraction from reports (e.g., piloted forms, independently, in duplicate) and any processes for obtaining and confirming data from investigators. | 4 |
| Data items | 11 | List and define all variables for which data were sought (e.g., PICOS, funding sources) and any assumptions and simplifications made. | 4 |
| Risk of bias in individual studies | 12 | Describe methods used for assessing risk of bias of individual studies (including specification of whether this was done at the study or outcome level), and how this information is to be used in any data synthesis. | 4 |
| Summary measures | 13 | State the principal summary measures (e.g., risk ratio, difference in means). | Not applicable. |
| Synthesis of results | 14 | Describe the methods of handling data and combining results of studies, if done, including measures of consistency (e.g., I^2^) for each meta-analysis. | Not applicable. |
| Risk of bias across studies | 15 | Specify any assessment of risk of bias that may affect the cumulative evidence (e.g., publication bias, selective reporting within studies). | Not applicable. |
| Additional analyses | 16 | Describe methods of additional analyses (e.g., sensitivity or subgroup analyses, meta-regression), if done, indicating which were pre-specified. | Not applicable. |
| **RESULTS** | | |  |
| Study selection | 17 | Give numbers of studies screened, assessed for eligibility, and included in the review, with reasons for exclusions at each stage, ideally with a flow diagram. | 5 and Figure 1 |
| Study characteristics | 18 | For each study, present characteristics for which data were extracted (e.g., study size, PICOS, follow-up period) and provide the citations. | 5-9 and Tables 3-8 |
| Risk of bias within studies | 19 | Present data on risk of bias of each study and, if available, any outcome level assessment (see item 12). | Not applicable. |
| Results of individual studies | 20 | For all outcomes considered (benefits or harms), present, for each study: (a) simple summary data for each intervention group (b) effect estimates and confidence intervals, ideally with a forest plot. | Not applicable. |
| Synthesis of results | 21 | Present results of each meta-analysis done, including confidence intervals and measures of consistency. | Not applicable. |
| Risk of bias across studies | 22 | Present results of any assessment of risk of bias across studies (see Item 15). | Not applicable. |
| Additional analysis | 23 | Give results of additional analyses, if done (e.g., sensitivity or subgroup analyses, meta-regression [see Item 16]). | Not applicable. |
| **DISCUSSION** | | |  |
| Summary of evidence | 24 | Summarize the main findings including the strength of evidence for each main outcome; consider their relevance to key groups (e.g., healthcare providers, users, and policy makers). | 10 |
| Limitations | 25 | Discuss limitations at study and outcome level (e.g., risk of bias), and at review-level (e.g., incomplete retrieval of identified research, reporting bias). | 11 |
| Conclusions | 26 | Provide a general interpretation of the results in the context of other evidence, and implications for future research. | 11 |
| **FUNDING** | | |  |
| Funding | 27 | Describe sources of funding for the systematic review and other support (e.g., supply of data); role of funders for the systematic review. | 11 |

**Table 2. Full search strategies.**

| Database | Date | Results | Search strategy |
| --- | --- | --- | --- |
| PubMed (MEDLINE) | 31 Dic 2021 | 173 | ((("Methylmalonic acidemia with homocystinuria"[Supplementary Concept] OR "Methylmalonic acidemia with homocystinuria"[All Fields] OR "cobalamin c methylmalonic acidemia and homocystinuria"[All Fields] OR ("cobalamin c methylmalonic acidemia"[Title/Abstract] AND "Homocystinuria"[Title/Abstract]) OR ("Methylmalonic acidemia with homocystinuria"[Supplementary Concept] OR "Methylmalonic acidemia with homocystinuria"[All Fields] OR "methylmalonic acidemia and homocystinuria cblc type"[All Fields]) OR ("methylmalonic acidemia"[Title/Abstract] AND "homocystinuria cblc type"[Title/Abstract]) OR ("Methylmalonic acidemia with homocystinuria"[Supplementary Concept] OR "Methylmalonic acidemia with homocystinuria"[All Fields] OR "methylmalonic aciduria and homocystinuria cblc type"[All Fields]) OR ("methylmalonic aciduria"[Title/Abstract] AND "homocystinuria cblc type"[Title/Abstract]) OR ("Methylmalonic acidemia with homocystinuria"[Supplementary Concept] OR "Methylmalonic acidemia with homocystinuria"[All Fields]) OR ("methylmalonic aciduria"[Title/Abstract] AND (("Homocystinuria"[MeSH Terms] OR "Homocystinuria"[All Fields] OR "homocystinurias"[All Fields]) AND "vitamin b12 responsive"[Title/Abstract])) OR ("Methylmalonic acidemia with homocystinuria"[Supplementary Concept] OR "Methylmalonic acidemia with homocystinuria"[All Fields] OR "methylmalonic acidemia and homocystinemia"[All Fields]) OR ("methylmalonic acidemia"[Title/Abstract] AND "homocystinemia"[Title/Abstract]) OR ("Methylmalonic acidemia with homocystinuria"[Supplementary Concept] OR "Methylmalonic acidemia with homocystinuria"[All Fields] OR "methylmalonic acidemia and homocystinuria cblc type"[All Fields]) OR ("methylmalonic acidemia"[Title/Abstract] AND "homocystinuria cblc type"[Title/Abstract]) OR "Methylmalonic acidemia with homocystinuria"[Supplementary Concept] OR "MMA"[All Fields] OR "MMA"[Title/Abstract] OR ("methylmalonate"[All Fields] OR "Methylmalonic"[All Fields]) OR "Methylmalonic"[Title/Abstract] OR (("methylmalonic acidemia"[Supplementary Concept] OR "methylmalonic acidemia"[All Fields] OR "methylmalonic aciduria"[All Fields]) AND ("Homocystinuria"[MeSH Terms] OR "Homocystinuria"[All Fields] OR "homocystinurias"[All Fields])) OR "methylmalonic aciduria homocystinuria"[Title/Abstract] OR ("cobalamin c methylmalonic acidemia"[Title/Abstract] AND "Homocystinuria"[Title/Abstract]) OR ("Homocysteine"[MeSH Terms] OR ("homocystein"[All Fields] OR "Homocysteine"[MeSH Terms] OR "Homocysteine"[All Fields] OR "homocysteine s"[All Fields] OR "homocysteines"[All Fields]) OR ("Homocysteine"[Title/Abstract] AND "2 amino 4 mercaptobutyric acid"[Title/Abstract]) OR ("Homocysteine"[MeSH Terms] OR "Homocysteine"[All Fields] OR "2 amino 4 mercaptobutyric acid"[All Fields]) OR "2 amino 4 mercaptobutyric acid"[Title/Abstract] OR ("Homocysteine"[MeSH Terms] OR "Homocysteine"[All Fields] OR "2 amino 4 mercaptobutyric acid"[All Fields]) OR (("homocystein"[All Fields] OR "Homocysteine"[MeSH Terms] OR "Homocysteine"[All Fields] OR "homocysteine s"[All Fields] OR "homocysteines"[All Fields]) AND "L-Isomer"[Title/Abstract]) OR ("Homocysteine"[MeSH Terms] OR "Homocysteine"[All Fields]) OR (("homocystein"[All Fields] OR "Homocysteine"[MeSH Terms] OR "Homocysteine"[All Fields] OR "homocysteine s"[All Fields] OR "homocysteines"[All Fields]) AND "L-Isomer"[Title/Abstract]) OR ("Homocysteine"[MeSH Terms] OR "Homocysteine"[All Fields]) OR ("L-Isomer"[All Fields] AND "Homocysteine"[Title/Abstract]) OR ("Homocysteine"[MeSH Terms] OR "Homocysteine"[All Fields]))) AND ("Vitamin B 12 Deficiency"[MeSH Terms] OR ("Vitamin B 12 Deficiency"[MeSH Terms] OR "Vitamin B 12 Deficiency"[All Fields] OR ("Vitamin"[All Fields] AND "b12"[All Fields] AND "deficiency"[All Fields]) OR "vitamin b12 deficiency"[All Fields]) OR ("Vitamin B 12 Deficiency"[MeSH Terms] OR "Vitamin B 12 Deficiency"[All Fields] OR ("deficiency"[All Fields] AND "Vitamin"[All Fields] AND "b12"[All Fields]) OR "deficiency vitamin b12"[All Fields]) OR ("Vitamin B 12 Deficiency"[MeSH Terms] OR "Vitamin B 12 Deficiency"[All Fields] OR ("deficiencies"[All Fields] AND "Vitamin"[All Fields] AND "b12"[All Fields]) OR "deficiencies vitamin b12"[All Fields]) OR ("Vitamin B 12 Deficiency"[MeSH Terms] OR "Vitamin B 12 Deficiency"[All Fields] OR ("Vitamin"[All Fields] AND "b12"[All Fields] AND "deficiencies"[All Fields]) OR "vitamin b12 deficiencies"[All Fields]) OR (("vitamin b 12"[MeSH Terms] OR "vitamin b 12"[All Fields] OR ("Vitamin"[All Fields] AND "b12"[All Fields]) OR "vitamin b12"[All Fields]) AND "definciencies"[All Fields]) OR ("Vitamin B 12 Deficiency"[MeSH Terms] OR "Vitamin B 12 Deficiency"[All Fields] OR "deficiency vitamin b 12"[All Fields]) OR "MMACHC"[All Fields] OR "cblc"[All Fields] OR ("cobalamine"[All Fields] OR "cobalamines"[All Fields] OR "vitamin b 12"[MeSH Terms] OR "vitamin b 12"[All Fields] OR "cobalamin"[All Fields] OR "cobalamins"[All Fields]) OR ("vitamin b12 deficiency"[Title/Abstract] OR "deficiency vitamin b12"[Title/Abstract] OR "deficiencies vitamin b12"[Title/Abstract] OR "vitamin b12 deficiencies"[Title/Abstract] OR "deficiency vitamin b 12"[Title/Abstract] OR "MMACHC"[Title/Abstract] OR "cblc"[Title/Abstract] OR "cobalamin"[Title/Abstract])) AND ("Age of Onset"[MeSH Terms] OR "onset age"[Title/Abstract] OR (("Age of Onset"[MeSH Terms] OR ("age"[All Fields] AND "onset"[All Fields]) OR "Age of Onset"[All Fields] OR ("onset"[All Fields] AND "age"[All Fields]) OR "onset age"[All Fields]) AND "age-at-onset"[Title/Abstract]) OR ("Age of Onset"[MeSH Terms] OR ("age"[All Fields] AND "onset"[All Fields]) OR "Age of Onset"[All Fields] OR ("age"[All Fields] AND "onset"[All Fields]) OR "age-at-onset"[All Fields]) OR "age-at-onset"[Title/Abstract] OR "Late Onset Disorders"[MeSH Terms] OR "adult onset"[Title/Abstract] OR (("adult"[MeSH Terms] OR "adult"[All Fields] OR "adults"[All Fields] OR "adult s"[All Fields]) AND ("Age of Onset"[MeSH Terms] OR ("age"[All Fields] AND "onset"[All Fields]) OR "Age of Onset"[All Fields] OR "onset"[All Fields] OR "onsets"[All Fields] OR "onsetting"[All Fields]) AND "late onset"[Title/Abstract]) OR ("late"[All Fields] AND ("Age of Onset"[MeSH Terms] OR ("age"[All Fields] AND "onset"[All Fields]) OR "Age of Onset"[All Fields] OR "onset"[All Fields] OR "onsets"[All Fields] OR "onsetting"[All Fields])))) OR ("European journal of pediatrics"[Jour] AND 2014[pdat] AND reversible pulmonary arterial)) |
| Abbreviations: Mesh = Medical Subject Headings | | | |

**Table 3. Quality assessment of the case reports/case series included in this systematic review.**

| **Authors and year** | **Yes** | **No** | **N/A** | **% yes** |
| --- | --- | --- | --- | --- |
| Ardissino et al. 2017 | 20 | 5 | 5 | 66.66 |
| Augoustides-Savvopoulou et al. 1999 | 16 | 7 | 9 | 53.33 |
| Backe et al. 2013 | 20 | 4 | 6 | 66.66 |
| Ben-Omran et al. 2007 | 17 | 9 | 4 | 56.66 |
| Bodamer et al. 2001 | 12 | 4 | 14 | 40 |
| Boxer et al. 2005 | 19 | 6 | 5 | 63.33 |
| Brox-Torrecilla et al. 2021 | 20 | 6 | 4 | 66.66 |
| Brunelli et al. 2002 | 13 | 13 | 4 | 43.33 |
| Collison et al. 2015 | 17 | 8 | 5 | 56.66 |
| Cornec-Legall et al. 2014 | 21 | 5 | 4 | 70 |
| Cui et al. 2019 | 21 | 3 | 6 | 70 |
| Davin et al. 2009 | 19 | 7 | 4 | 63.33 |
| Gilson et al. 2018 | 14 | 5 | 11 | 46.66 |
| Gold et al. 1996 | 11 | 9 | 10 | 36.66 |
| Goodman et al. 1970 | 7 | 13 | 10 | 23.33 |
| Grandone et al. 2019 | 19 | 6 | 5 | 63.33 |
| Grangé et al. 2015 | 9 | 11 | 10 | 30 |
| Guigonis et al. 2005 | 17 | 9 | 4 | 56.66 |
| Gündüz et al. 2014 | 19 | 5 | 6 | 63.33 |
| Gurkas et al. 2015 | 20 | 5 | 5 | 66.66 |
| Heil et al. 2007 | 14 | 12 | 4 | 46.66 |
| Higashimoto et al. 2019 | 23 | 3 | 4 | 76.66 |
| Huemer et al. 2014 | 18 | 3 | 9 | 60 |
| Iodice et al. 2013 | 17 | 8 | 5 | 56.66 |
| Jiménez-Varo et al. 2015 | 11 | 15 | 4 | 36.66 |
| Killiç et al. 2013 | 15 | 9 | 6 | 50 |
| Koenig et al. 2015 | 19 | 7 | 4 | 63.33 |
| Kömhoff et al. 2013 | 18 | 8 | 4 | 60 |
| Lin et al. 2009 | 15 | 8 | 7 | 50 |
| Liu et al. 2015 (1) | 16 | 3 | 11 | 53.33 |
| Liu et al. 2017 | 22 | 5 | 3 | 73.33 |
| Losito et al. 2012 | 17 | 9 | 4 | 56.66 |
| Mitchell et al. 1986 | 20 | 5 | 5 | 66.66 |
| Navarro et al. 2018 | 21 | 5 | 4 | 70 |
| Petropoulos et al. 2018 | 22 | 4 | 4 | 73.33 |
| Philipponnet et al. 2020 | 22 | 4 | 4 | 73.33 |
| Pollini et al. 2020 | 19 | 7 | 4 | 63.33 |
| Powers et al. 2001 | 12 | 13 | 5 | 40 |
| Rahmandar et al. 2014 | 21 | 6 | 3 | 70 |
| Roze et al. 2003 | 17 | 8 | 5 | 56.66 |
| Shinnar et al. 1984 | 16 | 4 | 10 | 53.33 |
| Thauvin-Robinet et al. 2007 | 20 | 5 | 5 | 66.66 |
| Tsai et al. 2007 | 20 | 6 | 4 | 66.66 |
| Van Hove et al. 2002 | 16 | 10 | 4 | 53.33 |
| Wang et al. 2012 | 15 | 11 | 4 | 50 |
| Wang et al. 2019 (1) | 13 | 13 | 4 | 43.33 |
| Wang et al. 2019 (4) | 7 | 13 | 10 | 23.33 |
| Wei et al. 2020 | 13 | 9 | 8 | 43.33 |
| Wen et al. 2020 | 24 | 3 | 3 | 80 |
| Wu et al. 2017 | 19 | 3 | 8 | 63.33 |
| Wu et al. 2018 | 9 | 12 | 9 | 30 |
| Zhao et al. 2021 | 19 | 7 | 4 | 63.33 |
| Total % Yes | 56.74 | | | |

**Table 4. General characteristics of the studies included in the systematic review**

| **First author^1^** | **Year of publication** | **Country^2^** | **Study title** | **Main objective** | **Type of study (study design)** | **Funding** |
| --- | --- | --- | --- | --- | --- | --- |
| Ardissino et al. | 2017 | Italy | Late onset cobalamin disorder and hemolytic uremic syndrome: a rare cause of nephrotic syndrome | To describe a case of atypical HUS (aHUS) related to CblC disease which first presented in a previously healthy boy at age of 13.6 years | Case report | None |
| Augoustides-Savvopoulou et al. | 1999 | Greece  Germany  Canada | Reversible dementia in an adolescent with cblC disease: Clinical heterogeneity within the same family | To describe a girl with progressive dementia that responded dramatically to intramuscular OHCbl with the objective of demonstrating clues to diagnosis, the importance of monitoring therapy, and clinical heterogeneity within the same family | Case report | None |
| Backe et al. | 2013 | Norway  Switzerland | Novel deletion mutation identified in a patient with late-onset combined methylmalonic acidemia and homocystinuria, cblc type | To report a novel homozygous deletion mutation (NM_015506.2: c.392_394del) resulting in an in-frame deletion of amino acid Gln131 and late-onset disease in a 23-year-old male | Research report | This work was supported by grants from University of Oslo and the Norwegian Research Council of Norway and the South-East Health Authority of Norway. B.F. was supported by a grant from Swiss National Science Foundation (320000_122568 and 31003A_138521). P.H.B. also received grants from “Legatet til Henrik Homans Minde” and “Dr. F€ urst medisinske laboratoriums fond til klinisk kjemisk og klinisk fysiologisk forskning” |
| Ben-Omran et al. | 2007 | Canada | Late-Onset Cobalamin-C Disorder: A Challenging Diagnosis | To describe clinical and biochemical findings of two unrelated patients with late-onset cblC disease who presented with neuropsychiatric symptoms | Case report | None |
| Bodamer et al. | 2001 | Canada  USA | Adult-onset combined methylmalonic aciduria and homocystinuria (cblC) | To report a case with documented cblC who presented during adulthood | Case report | None |
| Boxer et al. | 2005 | USA | Executive dysfunction in hyperhomocystinemia responds to homocysteine lowering treatment | To report a late-onset case of hyperhomocystinemia due to a vitamin B12 metabolic deficit (cobalamin C) with cognitive impairment, primarily in frontal/executive function. | Case report | None |
| Brox-Torrecilla et al. | 2021 | Spain | Late-onset methylmalonic acidemia and homocysteinemia: a case report | To report the case of a 45-year-old man with a 20-year history of chronic kidney disease and recently diagnosed spastic paraparesis, both of unknown origin. | Case report | None |
| Brunelli et al. | 2002 | USA | Cobalamin C deficiency complicated by an atypical glomerulopathy | To report a case of an atypical glomerulopathy in a 16-year-old male patient with cbl C deficiency. | Case report | None |
| Chang et al. | 2020 | China | Adolescent/adult-onset homocysteine remethylation disorders characterized by gait disturbance with/without psychiatric symptoms and cognitive decline: a series of seven cases | To analyze the clinical and genetic characteristics of seven cases with adolescent/adult remethylation disorders, including 5 cases of the cobalamin C disease (cblC) and 2 cases of the methylenetetrahydrofolate reductase deficiency. | Original research | None |
| Chu et al. | 2020 | China  USA | Peripheral nervous system involvement in late-onset cobalamin c disease? | To verify and summarize the clinical, electrophysiological, and pathological features of peripheral nerve involvement in late-onset cblC disease. | Original article | None |
| Collison et al. | 2015 | USA | Whole exome sequencing identifies an adult-onset case of methylmalonic aciduria and homocystinuria type C (CblC) with non-syndromic bull’s eye maculopathy | To report a late-onset case of an adult patient with bull’s eye macular lesions and no clinically relevant systemic symptoms was diagnosed with cblC by genetic screening and follow-up biochemical laboratory tests. | Case report | This study is supported in part by NIH grants EY021163, EY019861, HG006542 and EY019007 (Core Support for Vision Research), by unrestricted funds from Research to Prevent Blindness (New York, NY) to the Department of Ophthalmology, Columbia University, and by the Pangere Family Corporation, The Chicago Lighthouse for People Who Are Blind or Visually Impaired (Chicago, IL). |
| Cornec-Legall et al. | 2014 | France | Adult-onset eculizumab-resistant hemolytic uremic syndrome associated with cobalamin c deficiency | To report a case of adult-onset HUS secondary to Cbl C disease. | Case report | None |
| Cui et al. | 2019 | China | Isolated subacute combined degeneration in late-onset cobalamin C deficiency in children. Two case reports and literature review | To report 2 pediatric cases of SCD in late-onset cobalamin C (CblC) deficiency. | Case report | This work was supported by the Key Research Program in Medical Science of Hebei province, China (No. 20180632) |
| Davin et al. | 2009 | The Netherlands  UK | Prophylactic plasma exchange in CD46-associated atypical haemolytic uremic syndrome | Here, we describe the effect of this strategy in a child with aHUS and a CD46 mutation. | Case report | None |
| Gerth et al. | 2008 | Canada | Ocular phenotype in patients with methylmalonic aciduria and homocystinuria, cobalamin C type | To assess and compare longitudinal visual function and retinal morphology in patients with methylmalonic aciduria with homocystinuria, cobalamin C type (cblC), and identified mutations in the MMACHC gene. | Original article  Longitudinal | This study was funded in part by Brandan’s Eye Research Fund. |
| Gilson et al. | 2018 | USA | Dementia, diarrhea, desquamating shellac-like dermatitis revealing late-onset cobalamin C deficiency | To present a case of late-onset cobalamin C deficiency presenting with a unique shellac-like erosive desquamation and mental status changes that was dramatically responsive to treatment. | Case report | None |
| Gold et al. | 1996 | Germany | Hereditary defect of cobalamin metabolism (homocystinuria and methylmalonic aciduria) of juvenile onset | To present a case of a 30 year old woman (patient 1) with a 13 year disease course of relapsing and remitting myelopathy and neuropathy due to the cblC defect and her 34 year old sister (patient 2) are reported. | Case report | None |
| Goodman et al. | 1970 | USA | Homocystinuria with methylmalonic aciduria: two cases in a sibship | To report upon two brothers, each of whom has both homocystinuria and methylmalonic aciduria. Such an association has been reported only once before | Case report | None |
| Grandone et al. | 2019 | Italy  Russia | Prospective evaluation of pregnancy outcome in an Italian woman with late-onset combined homocystinuria and methylmalonic aciduria | To report the case of a patient who was apparently healthy until the age of 20, when she presented with impaired renal function and normocytic anaemia. | Case report | None |
| Grangé et al. | 2015 | France | Adult-onset renal thrombotic microangiopathy and pulmonary arterial hypertension in cobalamin C deficiency | To report an 18-year old who presented with kidney failure, pulmonary hypertension, haemolytic anaemia, and thrombocytopenia. | Case report | None |
| Guigonis et al. | 2005 | France  Canada | Late-onset thrombocytic microangiopathy caused by cblC disease: association with a factor H mutation | To report on 2 patients with cblC disease who had HUS during childhood without any neurologic involvement. A factor H mutation was seen in the patient with the most severe presentation. | Case report | None |
| Gündüz et al. | 2014 | Turkey  Spain | Reversible pulmonary arterial hypertension in cobalamin-dependent cobalamin C disease due to a novel mutation in the MMACHC gene | To present a case with CblC disease and pulmonary arterial hypertension (PAH) as the main symptom. | Case report | None |
| Gurkas et al. | 2015 | Turkey | Reversible clinical and magnetic resonance imaging findings in late-onset Cobalamina C defect | To describe an 8-year-old with late-onset Cbl C disease presenting with neuropsychiatric symptoms. | Case report | None |
| Heil et al. | 2007 | The Netherlands | Marfanoid features in a child with combined methylmalonic aciduria and homocystinuria (CblC type) | To describe two siblings of a consanguineous family: a girl who presented with anaemia, cognitive regression and Marfanoid features at the age of 13 years, and her brother who presented at the age of 10 months with motor and mental retardation. They discuss the clinical, biochemical and molecular findings in these patients and present the clinical and biochemical effects of therapy. | Case report | This study was supported by grants from the Dutch Kidney Foundation (C011928) and the Netherlands Heart Foundation (1999T023). |
| Higashimoto et al. | 2019 | USA  Saudi Arabia | High-dose hydroxocobalamin achieves biochemical correction and improvement of neuropsychiatric deficits in adults with late onset cobalamin C deficiency | To report three adult siblings with late onset cblC disease, and their biochemical and clinical responses to high-dose hydroxocobalamin. | Case report | National Institute of General Medical Sciences, Grant/Award Number: GM007471; National Human Genome Research Institute, Grant/Award Number: Intramural Research Program; Johns Hopkins University; NIH, Grant/Award Number: T32GM007471 |
| Huemer et al. | 2014 | Switzerland | Three new cases of late-onset cblC defect and review of the literature illustrating when to consider inborn errors of metabolism beyond infancy | To review the published clinical data and add three new cases to raise awareness for this severe but often treatable disease. | Case report Review | This work was supported by the Swiss National Science Foundation [grant number 31003A_138521 to M.R.B. and B.F.] and the Rare Disease Initiative Zurich (radiz), a clinical research priority program for rare diseases of the University of Zurich, Switzerland. |
| Iodice et al. | 2013 | Italy | Cobalamin C defect presenting with isolated pulmonary hypertension | To report a case of a young boy with cblC defect, who did not undergo newborn screening, presenting at the age of 2 years with isolated pulmonary hypertension as the leading symptom. | Case report | Dr Dionisi-Vici was supported by the grant “CCM 2010: Costruzione di percorsi diagnostico-assistenziali per le malattie oggetto di screening neonatale allargato” from the Italian Ministry of Health. |
| Jiménez-Varo et al. | 2015 | Spain | [Combined methylmalonic acidemia and homocystinuria; a case report] | To report the case of a patient of 18 years with a history of epilepsy who consults for acute renal failure requiring renal replacement therapy and diagnosed with combined methylmalonic acidemia and homocystinuria cblC variant | Case report | None |
| Kılıç et al. | 2013 | Turkey  Switzerland | Cobalamin C defect: a patient of late-onset type with homozygous p.R132* mutation | To present a Turkish patient who had neurological impairment at the age of four years as presented with late-onset cblC defect. | Case report | None |
| Koenig et al. | 2015 | Germany  Switzerland | Nephrotic syndrome and thrombotic microangiopathy caused by cobalamin C deficiency | To describe a patient with CblC deficiency presenting with nephrotic range proteinuria and arterial hypertension. | Case report | None |
| Kömhoff et al. | 2013 | The Netherlands  Italy | Combined pulmonary hypertension and renal thrombotic microangiopathy in cobalamin C deficiency | To investigate the clinical, biochemical, and genetic aspects of 5 children with the rare combination of PAH and rTMA. | Case series | None |
| Lemoine et al. | 2018 | France  Belgium | Cobalamin C deficiency induces a typical histopathological pattern of renal arteriolar and glomerular thrombotic microangiopathy | To describe the characteristics of kidney disease in cblC deficiency, and to provide a comparative histological analysis with cblC-independent renal TMA. | Original article | None |
| Li et al. | 2015 | China | Clinical characteristics of hemolytic uremic syndrome secondary to cobalamin C disorder in Chinese children | To present with clinical characteristics of 3 Chinese children with HUS secondary to cbl-C disorder | Original article | The study was supported by a grant from the Special Program for Clinical Research of the Beijing Municipal Commission of Science and Technology and WU JIE PING Medical Foundation, China (Grant No. Z121107005112008) |
| Lin et al. | 2009 | USA  Canada | Asymptomatic maternal combined homocystinuria and methylmalonic aciduria (cblC) detected through low carnitine levels on newborn screening | To report the first example of maternal cblC disorder presenting with low carnitine on newborn screening. | Case report | None |
| Liu et al. (1) | 2015 | China | Clinical analysis of late-onset methylmalonic acidaemia and homocystinuria, cblC type with a neuropsychiatric presentation | To report five cases of late-onset cblC with a neuropsychiatric presentation. | Case report | This study was supported by a grant from the Foundation of He’nan Educational Committee (No. 14A360010). |
| Liu et al. (2) | 2015 | China | First Chinese case of successful pregnancy with combined methylmalonic aciduria and homocystinuria, cblC type | To report the first case of a Chinese woman with cblC undergoing a successful pregnancy and delivering a healthy boy. | Original article | This work was supported by the National Nature Science Foundation of China (No. 30872794), the 12^th^ Five-year Plan National Key Technology R & D Program from the Ministry of Science and Technology (2012BAI09B04). |
| Liu et al. | 2017 | China | Combined methylmalonic acidemia and homocysteinemia presenting predominantly with late-onset diffuse lung disease: a case series of four patients | To report 4 children with combined MMA and homocysteinemia who presented predominantly with late-onset diffuse lung diseases (DLD). | Letter to the Editor | This work was supported by Beijing Municipal and Commission Health and Family Planning [2015-3-076]. The funding body had no role in the design of the study and collection, analysis, and interpretation of data and in writing the manuscript. |
| Liu et al. | 2020 | China | Cobalamin C deficiency presenting with diffuse alveolar hemorrhage and pulmonary microangiopathy | To summarize the new clinical features mainly diffuse alveolar hemorrhage (DAH) in cblC deficiency. | Original article | Beijing Municipal and Commission Health and Family Planning, Grant/Award Number: 2015‐3‐076; The National Key Research and Development Program of China, Grant/Award Number: 2016YFC0901502 |
| Losito et al. | 2012 | Italy | Thrombotic microangiopathic nephropathy, pulmonary hypertension and nephromegaly: case report of a patient treated with endothelin receptor antagonist | To describe a 14-year-old boy with no prior medical history who presented with hypertension, proteinuria and nephromegaly, and then developed progressive pulmonary hypertension. | Case report | None |
| Mitchell et al. | 1986 | Canada | Clinical heterogeneity in cobalamin C variant of combined homocystinuria and methylmalonic aciduria | To describe two patients with methylmalonic aciduria and homocystinuria (Cbl C). | Case report | None |
| Navarro et al. | 2018 | Portugal | Atypical adult-onset methylmalonic acidemia and homocystinuria presenting as hemolytic uremic syndrome | To present a challenging case of a 19-year old woman who presented with thrombotic microangiopathy, which was found to be caused by methylmalonic acidemia and homocystinuria, a rare vitamin B12 metabolism deficiency. | Case report | None |
| Nogueira et al. | 2017 | Portugal | Molecular picture of cobalamin C/D defects before and after newborn screening era | To compare the genotype/phenotype of patients identified with CblC or CblD before and after the implementation of expanded newborn screening. | Original article | European Network and Registry for Homocystinurias and Methylation Defects – EHOD project (Nº2012_12_02) for the partial support of this work. |
| Petropoulos et al. | 2018 | Canada | Renal thrombotic microangiopathy and pulmonary arterial hypertension in a patient with late-onset cobalamin C deficiency | To report a case of late-onset cblC deficiency presenting with rTMA and PAH. | Case report | None |
| Philipponnet et al. | 2020 | France | Cobalamin c deficiency associated with antifactor h antibody-associated hemolytic uremic syndrome in a young adult | To report the first case of adult onset cobalamin C (Cbl C) disease associated with anti-factor H antibody-associated hemolytic uremic syndrome (HUS). | Case report | None |
| Pollini et al. | 2020 | Italy | Multiple sclerosis and intracellular cobalamin defect (MMACHC/ PRDX1) comorbidity in a young male | To report on the first case of a patient with comorbid cblC defect and MS. | Case report | None |
| Powers et al. | 2001 | Canada  USA | Neurological and neuropathologic heterogeneity in two brothers with cobalamin C deficiency | To report the spectrum of cblC and remind us that neuropsychiatric deficits due to cerebral white matter lesions can dominate in diseases of Cbl deficiency. | Case report | None |
| Profitlich et al. | 2009 | USA | High prevalence of structural heart disease in children with cblC-type methylmalonic aciduria and homocystinuria | To describe a high prevalence of clinically significant structural heart disease in a cohort of patients with cblC, suggesting that routine cardiovascular screening may be indicated in these patients. | Original article | None |
| Rahmandar et al. | 2014 | USA | Cobalamin C deficiency in an adolescent with altered mental status and anorexia | To report a case of an adolescent girl who presented with psychosis and anorexia and later developed seizures and ataxia due to cblC deficiency. | Case report | None |
| Roze et al. | 2003 | France | Neuropsychiatric disturbances in presumed late-onset cobalamin C disease | To describe clinical and biochemical features of the disease in 2 siblings affected with presumed late-onset cobalamin C disease. | Case report | None |
| Shinnar et al. | 1984 | USA | Cobalamin C mutation (methylmalonic aciduria and homocystinuria) in adolescence. A treatable cause of dementia and myelopathy | To describe an adolescent girl with progressive dementia and myelopathy secondary to a familial intracellular defect of B12 metabolism (cobalamin C mutation), whose symptoms and biochemical abnormalities improved markedly after she received large doses of hydroxocobalamin | Case report | None |
| Thauvin-Robinet et al. | 2007 | France | The adolescent and adult form of cobalamin C disease: clinical and molecular spectrum | To report on the clinical, metabolic and molecular spectrum of three new cases and follow-up of two previously reported cases of adolescent and adult onset Cbl-c disease. | Case report | None |
| Tsai et al. | 2007 | USA  Canada | Late-onset combined homocystinuria and methylmalonic aciduria (cblC) and neuropsychiatric disturbance | To report on the case of a 36-year-old Hispanic woman with a spinal cord infarct, who was subsequently diagnosed with methylmalonic aciduria and homocystinuria, cblC type (cblC). | Case report  Review | None |
| Van Hove et al. | 2002 | Belgium  Australia  The Netherlands  Switzerland | Cobalamin disorder cbl-C presenting with late-onset thrombotic microangiopathy | To describe two siblings with Cbl-C disorder who presented in childhood with chronic thrombotic microangiopathic syndrome without neurologic dysfunction. | Case report | None |
| Wang et al. | 2012 | China | A clinical and gene analysis of late-onset combined methylmalonic aciduria and homocystinuria, cblC type, in China | To describe the clinical presentation and imaging of three patients with late-onset combined methylmalonic aciduria and homocystinuria, cblC type, whose diagnoses were confirmed by genetic analysis. | Short communication | None |
| Wang et al. | 2018 | China | Late-onset cobalamin C deficiency Chinese sibling patients with neuropsychiatric presentations | To analyze the clinical presentations and treatment outcomes of late-onset cblC in Chinese sibling patients with neuropsychiatric presentations. | Original article | This work is supported by grants from Natural Science Foundation of Shandong Province, China (No.ZR2017MH082) and Innovative Research Project of Resident Standardization Training of Qilu Hospital, Shandong University (No.ZPZX2017B10) |
| Wang et al. (1) | 2019 | China  Japan | Mutation spectrum of MMACHC in Chinese pediatric patients with cobalamin C disease: A case series and literature review | To summarise the clinical phenotype data of 28 pediatric cblC probands and sequenced and analysed the MMACHC gene mutations in all pedigrees. To review and list the MMACHC gene mutation spectrum of 564 Caucasian and 341 Chinese patients with cblC disease and compared the similarities and differences among them. The relationship between the common mutations and the clinical phenotype, such as the age of onset and disease severity, was also analysed. | Case series  Review | This study was supported by the Natural Science Foundation of Tianjin City [grants numbers 16JCQNJC11900]; the Natural Science Foundation of China [grants numbers 81771589]; and the Program of Tianjin Science and Technology Plan [grants numbers 18ZXDBSY00170]. |
| Wang et al. (2) | 2019 | China | Clinical feature and outcome of late-onset cobalamin C disease patients with neuropsychiatric presentations: a Chinese case series | To analyse the clinical presentations, gene mutations, and treatment of Chinese patients with late-onset cblC disease. | Original article | This work was supported by grants from the Natural Science Foundation of Shandong Province, China (No. ZR2017MH082), the Innovative Research Project of Resident Standardization Training of Qilu Hospital, Shandong University (No. ZPZX2017B10), and the Taishan Scholars Program of Shandong Province. |
| Wang et al. (3) | 2019 | China | Distinct clinical, neuroimaging and genetic profiles of late-onset cobalamin C defects (cb1C): a report of 16 Chinese cases | To present, 16 Chinese cases with late-onset cobalamin C disorder were diagnosed and confirmed by mutation analysis of the MMACHC gene (NM_015506.2). The aim to characterize the clinical and neuroimaging profiles, as well as the mutational spectrum and genotype-phenotype correlation of the late-onset cblC cases. | Original article | None |
| Wang et al. (4) | 2019 | China | Reversible encephalopathy caused by an inborn error of cobalamin metabolism | To report a case with documented cblC who presented during adulthood. | Case report | Innovative Research Project of Resident Standardisation Training of Qilu Hospital, Shandong University supported production of this manuscript (No.ZPZX2017B10). |
| Wei et al. | 2019 | China | Treatable cause of hereditary spastic paraplegia: eight cases of combined homocysteinaemia with methylmalonic aciduria | To review the clinical and biochemical features of a cohort of eight patients with MMA/HCY that mimicked HSP. | Original article | None |
| Wei et al. | 2020 | China | Late-onset cobalamin C disease presenting with acute progressive polyneuropathy | To describe an adult man genetically diagnosed with cblC disease who presented with acute sensorimotor neuropathy associated with cognitive changes | Case report | None |
| Wen et al. | 2020 | China | Pulmonary hypertension in late-onset  Methylmalonic Aciduria and Homocystinemia: a case report | To report a 12-year-old girl with cblC deficiency, who presented with PH as her first symptom. | Case report | None |
| Wu et al. | 2017 | China | Manic-depressive psychosis as the initial symptom in adult siblings with late-onset combined methylmalonic aciduria and homocystinemia, cobalamin c type | To report two sibling cases of late-onset cblC disease that presented with manic-depressive psychosis as the first symptom. | Case report | This work was supported by a grant from the National Natural Science Foundation of China (No. 81470074). |
| Wu et al. | 2018 | China | Serial magnetic resonance imaging changes in a patient with late-onset cobalamin C disease with a misdiagnosis of metachromatic leukodystrophy | To report serial magnetic resonance changes in a 45-year-old man with cblC deficiency. | Case report | None |
| Zhao et al. | 2021 | China | Adult-onset hypoxemia, diffuse lung lesions, and pulmonary hypertension in cobalamin C defect: a case report | To present a case of severe PH with diffuse lung lesions secondary to cobalamin C (cbl-C) defect that has been successfully treated. | Case report | This study was funded by the National Natural Science Foundation of China (81870042 and 81900050), Natural Science Foundation of Shanghai (18ZR1431500), National Science and Technology Information System of the China (2018YFC1313603) and Program of Shanghai Municipal Commission of Health (20204Y0384). |
| **Notes: ^1^** Articles in alphabetical order according to the first author. **^2^** Considering authors´ affiliations.  **Abbreviations:** N/A: Not available | | | | | | |

**Table 5. Age at onset and diagnosis, patient history and clinical presentation**

| **Author and year** | **# of cases** | **Age at onset (years)** | **Age at diagnosis (years)** | **Sex**  **M/F** | **Background** | **Family history** | **Previous medical history** | **Clinical presentation** |
| --- | --- | --- | --- | --- | --- | --- | --- | --- |
| Ardissino et al. 2017 | 1 | 8 | 13.6 | M | Caucasian | Non consanguinity  Uneventful family history | Generalised seizure in apyrexia, severe headache, recurrent emesis, weight loss | Nephrotic syndrome,  microhematuria, severe hypertension,  TMA |
| Augoustides-Savvopoulou et al. 1999 **^1^** | 1 | 10 | 11 | F | Greek | Non consanguinity  Sister: undiagnosed neurological disease who died at 13 years of age | Uneventful medical history | Learning difficulties, behavioural changes, ataxia, myoclonic jerks |
| Backe et al. 2013 | 1 | 19 | 23 | M | Asian | N/A | Slightly impaired intellectual disability  Uneventful medical history | Atactic gait, numbness, partial paralysis in the lower limbs |
| Ben-Omran et al. 2007 | 1 | 12 | 14 | F | Pakistan | Consanguinity: cousins  Mutations inherited from parents | Depression, progressive dementia regression, school failure | Dementia, lactic acidosis, vasculitic-like peripheral neuropathy |
|  | 2 | 6.5 | 10 | F | Bengali descent | Non consanguinity | Learning disability, school failure, seizures | Acute dementia, anorexia, and progressive weight loss |
| Bodamer et al. 2001 **^1^** | 1 | 19 | 20 | M | Hispanic | Non consanguinity  Uneventful family history | Uneventful medical history | Bilateral paraplegia of the legs, loss of bowel and bladder function, chronic progressive encephalopathy, venous thrombosis |
| Boxer et al. 2005 **^1^** | 1 | 38 | 42 | M | Middle Eastern | Consanguinity: cousins  Case 1 and 2 were siblings | Hypertension | Seizures, cognitive deficits |
|  | 2 | 6 | N/A | F | Middle Eastern | Consanguinity: cousins  Case 1 and 2 were siblings | N/A | Seizures, dementia, paraplegia |
| Brox-Torrecilla et al. 2021 | 1 | 23 | 45 | M | Caucasian | Non consanguinity | Chronic kidney disease requiring haemodialysis | Neurological manifestations, thrombosis in the arteriovenous fistula, tonic-clonic seizures, acute encephalopathy with spastic paraparesis, neurogenic bladder, lower limb mobility |
| Brunelli et al. 2002 | 1 | 16 | 16 | M | Caucasian | Non consanguinity  Uneventful family history | Learning disability | Unsteady gait, toppling over, slowed speech, memory lapses, decreased attention span, and decreased comprehension, atypical glomerulopathy with overlapping features of MPGN and TMA that are not characteristic of either histopathological category |
| Chang et al. 2020 | 1 | 14 | 21 | F | China | N/A | N/A | 14 y: introversion 15 y: cognitive decline, gait disturbance, acute encephalopathy, dysarthria, lethargy, delirium, hallucinations, anorexia  Gait: Spastic paraplegia |
|  | 2 | 14 | 40 | M | China | Cases 2 and 3 were siblings. | N/A | 14 y: bipolar disorder 37 y: gait disturbance 38 y: dysarthria  Gait: Sensory ataxia |
|  | 3 | 17 | 45 | M | China | Cases 2 and 3 were siblings. | N/A | 17 y: paranoia 38 y: cataract 45 y: gait disturbance  Gait: Sensory ataxia |
|  | 4 | 35 | 35 | M | China | N/A | N/A | 35 y: gait disturbance, urinary incontinence  Gait: Sensory ataxia |
|  | 5 | 14 | 34 | M | China | N/A | N/A | 14 y: introversion, seizures, cognitive decline 27 y: gait disturbance 29 y: hand tremor, constipation  Gait: Steppage |
| Chu et al. 2020 | 1 | 13 | 17 | M | China | The parents were heterozygous carriers of the MMACHC gene  mutations  The parents were heterozygous carriers of the MTHFR gene mutations. | N/A | Major symptoms: Clinical cognitive deficit, limb weakness, vomiting, anorexia  Major signs: Hyporeflexia, hypesthesia |
|  | 2 | 24 | 24 | M | China | N/A | N/A | Major symptoms: Psychiatric changes, coma, clinical cognitive deficit, limb weakness, vomiting, anorexia  Major signs: Pyramidal signs, hyporeflexia, hypesthesia |
|  | 3 | 25 | 27 | M | China | The parents were heterozygous carriers of the MMACHC gene  mutations | N/A | Major symptoms: coma, clinical cognitive deficit, limb weakness  Major signs: Pyramidal signs |
|  | 4 | 14 | 16 | M | China | The parents were heterozygous carriers of the MMACHC gene  mutations | N/A | Major symptoms: clinical cognitive deficit, limb weakness  Major signs: Pyramidal signs |
|  | 5 | 20 | 22 | M | China | N/A | N/A | Major symptoms: clinical cognitive deficit, limb weakness, epilepsy, anorexia  Major signs: Pyramidal signs |
|  | 6 | 8 | 20 | F | China | N/A | N/A | Major symptoms: Psychiatric changes, clinical cognitive deficit, limb weakness  Major signs: Pyramidal signs |
|  | 7 | 14 | 14 | M | China | The parents were heterozygous carriers of the MMACHC gene  mutations | N/A | Major symptoms: Clinical cognitive deficit, limb weakness  Major signs: Pyramidal signs, Hyporeflexia |
|  | 8 | 12 | 13 | F | China | The parents were heterozygous carriers of the MMACHC gene  mutations | N/A | Major symptoms: Clinical cognitive deficit, limb weakness  Major signs: Pyramidal signs, Hyporeflexia |
| Collison et al. 2015 | 1 | 28 | 35 | F | Hispanic | A few fine drusen near the fovea were observed in the examinations of both parents. | Iron deficiency anemia, occasional urinary tract infections, recurrent kidney infections | Decreased central vision, mild photo aversion |
| Cornec-Legall et al. 2014 | 1 | 20 | 20 | M | Caucasian | Non consanguinity.  Uneventful family history | Uneventful medical history | Malignant hypertension, kidney failure, hemolysis, asthenia |
| Cui et al. 2019 | 1 | 13 | 13 | F | China | Uneventful family history  Mutations inherited from parents  Cases 1, 2 and 3 were siblings. | N/A | Unsteady walking, unwillingness to communicate with others |
|  | 2 | N/A | N/A | N/A | China | Uneventful family history  Mutations inherited from parents  Cases 1, 2 and 3 were siblings. | N/A | N/A |
|  | 3 | N/A | N/A | N/A | China | Uneventful family history  Mutations inherited from parents  Cases 1, 2 and 3 were siblings. | N/A | N/A |
|  | 4 | 6 | 6 | M | China | Mutations inherited from parents.  Cases 4 and 5 were siblings. | Uneventful medical history | Unsteady walking |
|  | 5 | N/A | N/A | F | China | Mutations inherited from parents.  Cases 4 and 5 were siblings. | N/A | Weakness in both lower extremities |
| Davin et al. 2009 | 1 | 3 | 3 | M | N/A | N/A | N/A | Renal failure, haemolytic anaemia, thrombocytopenia, hypertension |
| Gerth et al. 2008 | 1 | 12 | N/A | F | N/A | Consanguinity | N/A | Progressive dementia, mood disorder, seizures, peripheral, neuropathy, lactic acidosis |
|  | 2 | 6 | N/A | F | N/A | N/A | N/A | Weight loss, developmental delay,  seizure disorder, encephalopathy |
|  | 3 | 12 | N/A | F | N/A | N/A | N/A | Developmental delay, behavioral  difficulties, megaloblastic anemia |
|  | 4 | 6 | N/A | F | N/A | Consanguinity | N/A | Developmental delay, regression of skills, failure to thrive, severe behavioral problems, macrocytic anemia, arachnodactyly with Marfanoid habitus, osteopenia, seizures |
| Gilson et al. 2018 | 1 | 21 | 21 | M | N/A | N/A | Wheelchair-bound, autoimmune cerebritis, seizure disorder, recurrent deep-vein thrombosis | Intractable diarrhea acute and progressive reduction in mentation, skin desquamation |
| Gold et al. 1996 **^3^** | 1 | 12 | 25 | F | N/A | Non consanguinity.  Uneventful family history  Cases 1 and 2 were siblings. | Uneventful medical history | Gait disorder, fatigue, urinary incontinence |
|  | 2 | N/A | 31 | F | N/A | Non consanguinity.  Uneventful family history  Cases 1 and 2 were siblings. | N/A | Asymptomatic |
| Goodman et al. 1970 | 1 | 14 | 14 | M | Spanish American | Consanguinity  Cases 1 and 2 were siblings. | N/A | Psychiatric symptoms, mental retardation, incoordination |
|  | 2 | N/A | 1 | M | Spanish American | Consanguinity  Cases 1 and 2 were siblings. | Uneventful medical history | Asymptomatic |
| Grandone et al. 2019 | 1 | 20 | 34 | F | Caucasian | N/A | Uneventful medical history | Elevated inflammatory markers, impaired renal function, proteinuria, microhaematuria, normocytic anaemia. Late (20th week gestational age) pregnancy loss of a morphologically normal intrauterine growth restricted foetus |
| Grangé et al. 2015 **^1, 2^** | 1 | 18 | 18 | M | White-France | Mutations inherited from parents.  Cases 1 and 2 were siblings. | Language retardation | Dyspnea, haemolytic anaemia and mild thrombocytopenia, renal failure with nephrotic syndrome and haematuria, hypertension, edema |
|  | 2 | N/A | 18 | M | White-France | Mutations inherited from parents.  Cases 1 and 2 were siblings. | End-stage kidney disease of unknown origin | Pulmonary veno-occlusive disease associated with hypertrophic cardiomyopathy. |
| Guigonis et al. 2005 | 1 | N/A | 6 | F | White-France | Non consanguinity.  Cases 1 and 2 were siblings. | N/A | Microscopic hematuria, proteinuria with nephrotic syndrome, hypertension, severe renal failure, thrombosis (HUS) |
|  | 2 | N/A | 8.5 | F | White-France | Non consanguinity.  Cases 1 and 2 were siblings. | N/A | Microscopic hematuria, proteinuria with nephrotic syndrome (HUS) |
| Gündüz et al. 2014 | 1 | N/A | 1.3 | F | N/A | Consanguinity  Mutations inherited from parents  One of her siblings had died at 4 years because  of undefined renal failure. | N/A | Dyspnea, legs edema (PAH) |
| Gurkas et al. 2015 | 1 | 8 | 8 | F | N/A | Consanguinity | Mild learning disability | Slowing movements, slurring of speech,  psychotic behaviours, visual hallucinations |
| Heil et al. 2007 | 1 | 12 | 13 | F | Turkey | Consanguinity: second cousins  Case 1 and 2 were siblings  Mutations inherited from parents | Uneventful medical history | Concentration problems, extensive fatigability, loss of appetite, difficulties with daily task, significant macrocytic anaemia, Marfanoid features |
|  | 2 | 6 | 9 | M | Turkey | Consanguinity: second cousins  Case 1 and 2 were siblings  Mutations inherited from parents | He had psychomotor developmental delay  and speech delay from the age of 10 months.  He developed central obesity at the age of 5 years. | Attention deficit hyperactivity disorder and behavioural abnormalities |
| Higashimoto et al. 2019 | 1 | 28 | 28 | F | N/A | Mother: multiple sclerosis  Case 1, 2 and 3 were siblings | Migraine headaches  Vegetarian | Reduced finger dexterity, gait disturbance, severe paranoid, delusions, blurred vision, provoked deep venous thrombosis complicated by a pulmonary embolism. |
|  | 2 | 26 | 26 | F | N/A | Mother: multiple sclerosis  Case 1, 2 and 3 were siblings | Uneventful medical history | Asymptomatic |
|  | 3 | 29 | 29 | F | N/A | Mother: multiple sclerosis  Case 1, 2 and 3 were siblings | Hearing impairment of uncertain aetiology, unilateral renal hypoplasia, hypertension,  chronic renal insufficiency (stage 3a) | Asymptomatic |
| Huemer et al. 2014 | 1 | 14 | 24 | M | Portuguese | Non consanguinity.  The patient’s sister had died at age 18 months from an unknown condition | Learning deficits, admitted to a psychiatric ward due to “strange” behavior, anxiety and signs of depersonalization disorder | Psychiatric symptoms in adolescence and malignant hypertension with secondary renal failure and/or HUS in adulthood |
|  | 2 | 26 | 30 | M | Austrian | Non consanguinity  Uneventful family history | Uneventful medical history | Neuropathy, subacute and combined degeneration of the spinal cord, cognitive impairment, depression, thromboembolism |
|  | 3 | 32 | 34 | F | Moroccan descent | Uneventful family history | Uneventful medical history | Apathy, reversible white matter abnormalities, paresis and respiratory insufficiency |
| Iodice et al. 2013 | 1 | 2 | 2 | M | African ancestry | Non consanguinity.  Case 1 and 2 were siblings | At the age of 45 days, the patient was admitted because of breathing difficulties, pallor, fever, and anemia | Feeding difficulties, failure to thrive, anemia treated with iron supplementation, isolated pulmonary hypertension |
|  | 2 | 3 | N/A | M | African ancestry | Non consanguinity.  Case 1 and 2 were siblings | N/A | HUS |
| Jiménez-Varo et al. 2015 | 1 | 13 | 18 | F | N/A | Consanguinity: cousins  Mutations inherited from parents | Epilepsy, anemia | Severe asthenia, concentration problems, memory loss, HUS |
| Kılıç et al. 2013 | 1 | 4 | 4 ^4/12^ | F | Turkish descent | Consanguinity | N/A | Loss of speech, inability to walk, stereotypic hand-clapping movements, ataxia, difficulty swallowing solid food, anemia |
| Koenig et al. 2015 | 1 | 3.5 | 4 | M | Caucasian | Non consanguinity.  Uneventful family history | Uneventful medical history | Pneumonia, night sweat, arterial hypertension, nephrotic range proteinuria and microhematuria, macrocytic anemia |
| Kömhoff et al. 2013 | 1 | 1.5 | 1.5 | N/A | Spanish/  Turkish | Case 1 and 4 were siblings | N/A | Failure to thrive, cyanosis, gallop rhythm, right ventricular failure, tachydyspnea (PAH), TMA |
|  | 2 | 2.5 | 2.5 | N/A | Dutch | N/A | N/A | Longstanding fatigue, coughing, and failure to thrive, subcomatose state, pallor, cyanosis, tachydyspnea, hepatomegaly, systemic hypertension (PAH), and aHUS with renal failure |
|  | 3 | 3 | 3 | N/A | Dutch | N/A | N/A | Fatigue, atypical HUS, renal transplantation  Seven years later → PAH |
|  | 4 | 4 | 6.5 | N/A | Spanish/  Turkish | Case 1 and 4 were siblings | N/A | Fatigue, malaise, failure to thrive (rTMA)  2.5 years later → PAH |
|  | 5 | 14 | 32 | N/A | Italian | N/A | N/A | Fatigue, pallor, elevated systemic blood pressure (rTMA)  Five years later → PAH |
| Lemoine et al. 2018 | 1 | 18 | 18 | N/A | N/A | Case 1 and 2 were siblings | N/A | Hypertension, edema, neurologíc symptoms, hemolysis, rTMA, kidney failure (dialysis) |
|  | 2 | 18 | 15 years after his death, when his brother (patient 1) was diagnosed. | N/A | N/A | Case 1 and 2 were siblings | N/A | Hypertension, PAH, hemolysis, rTMA, kidney failure (dialysis) |
|  | 3 | 20 | 21 | N/A | N/A | N/A | N/A | Hypertension, edema, hemolysis, rTMA, kidney failure (dialysis) |
|  | 4 | 6 | 6.4 | N/A | N/A | Case 4 and 5 were siblings | N/A | Hypertension, edema, thromboembolic event, hemolysis, rTMA, kidney failure (dialysis) |
|  | 5 | 8.5 | 8.6 | N/A | N/A | Case 4 and 5 were siblings | N/A | Hemolysis, rTMA, kidney failure |
|  | 6 | 15 | 15.1 | N/A | N/A | N/A | N/A | Hypertension, hemolysis, rTMA, kidney failure (dialysis) |
|  | 7 | 26 | 33 | F | N/A | N/A | Diagnosed with renal TMA during pregnancy and received a kidney transplant 5 years later | Hypertension, edema, hemolysis, rTMA, kidney failure (kidney transplant) |
| Li et al. 2015 | 1 | 1.5 | 1.5 | M | Chinese-Mongolian ethnic | Non consanguinity.  Brother with early onset Cbl C died at 10 months. | Anemia | Anemia, edema of eyelids, hematuria, poor feeding, failure to thrive, HUS |
|  | 2 | 3.3 | 3.3 | F | Chinese-Mongolian ethnic | N/A | Uneventful medical history | Anemia, fever, edema in face, poor feeding, failure to thrive, fever, hypertension, kidney failure (rTMA) |
|  | 3 | 2.7 | 3.7 | M | Chinese Han ethnic | N/A | Iron deficiency anemia | Anemia, poor feeding, failure to thrive, kidney failure, hypertension, multiple organ injury |
| Lin et al. 2009 | 1 | 29 | 29 | F | Hispanic | Her parents were from ranchos (<50 houses) in Guerrero, Mexico | Uneventful medical history | Asymptomatic detected through low carnitine levels on new-born screening |
| Liu et al. 2015 (1) | 1 | 17 | 35 | M | N/A | No family history of cblC. | Normal physical appearance, introverted personality, delayed learning, memory abilities and decreased locomotor activities during childhood. | Symptoms of mental and psychiatric disorders |
|  | 2 | 14 | 16 | M | N/A | No family history of cblC. | Normal physical appearance, introverted personality, delayed learning, memory abilities and decreased locomotor activities during childhood.  Anorexic to meat and eggs | Symptoms of mental and psychiatric disorders |
|  | 3 | 7 | 13 | M | N/A | No family history of cblC. | Normal physical appearance,  introverted personality, delayed learning, memory abilities and decreased locomotor activities during childhood.  Anorexic to meat and eggs | Symptoms of gait and posture abnormalities |
|  | 4 | 12 | 12 | F | N/A | No family history of cblC. | Normal physical appearance, introverted personality, delayed learning, memory abilities and decreased locomotor activities during childhood.  Anorexic to meat and eggs | Symptoms of mental and psychiatric disorders |
|  | 5 | 9 | 9 | F | N/A | No family history of cblC. | Normal physical appearance, introverted personality, delayed learning, memory abilities and decreased locomotor activities during childhood | Symptoms of gait and posture abnormalities |
| Liu et al. 2015 (2) | 1 | 14 | 15 | F | Chinese | Non consanguinity | Uneventful medical history | Learning difficulty, leg weakness |
| Liu et al. 2017 | 1 | 1.25 | 1.75 | F | N/A | Mutations inherited from parents | Fever, pulmonary consolidation, pleural effusions | Cough, dyspnea, PAH |
|  | 2 | 3 | 4.7 | F | N/A | Not included | Lower than expected levels of physical activity, and slightly delayed intellectual and language development since birth. | Slightly delayed intellectual and language development, cough, short of breath, PAH |
|  | 3 | 2.5 | 8.5 | F | N/A | Mutations inherited from parents | Renal failure, hypertension, moderate anemia | Decreased activity, vomiting, diarrhea, abnormal renal function, cough, PAH  Mesangioproliferative glomerulonephritis, Renal TMA |
|  | 4 | 7.7 | 7.8 | M | N/A | Mutations inherited from parents | Acute glomerulonephritis with mild microscopic hematuria | Mild wet cough, shortness of breath  Mesangioproliferative glomerulonephritis, Renal TMA |
| Liu et al. 2020 | 1 | 6.5 | 7.5 | F | Asian | Mutations inherited from parents | Suspected of refractory idiopathic pulmonary hemosiderosis or interstitial lung disease (ILD) of unknown reasons treated with methylprednisolone | Cough, short of breath, megaloblastic anemia  PAH mild |
|  | 2 | 4.5 | 4.5 | M | Asian | Mutations inherited from parents | Suspected of refractory idiopathic pulmonary hemosiderosis or interstitial lung disease (ILD) of unknown reasons treated with methylprednisolone | Vomiting, fatigue, short of breath, kidney failure  PAH severe |
|  | 3 | 4.1 | 4.1 | M | Asian | Mutations inherited from parents | Suspected of refractory idiopathic pulmonary hemosiderosis or interstitial lung disease (ILD) of unknown reasons treated with methylprednisolone.  Respiratory failure (type I) treated with pulsed methylprednisolone and nasal continuous positive airway pressure. | Vomiting, fatigue, dyspnea, megaloblastic anemia  PAH moderate |
|  | 4 | 4.1 | 6.1 | F | Asian | Mutations inherited from parents | Suspected of refractory idiopathic pulmonary hemosiderosis or interstitial lung disease (ILD) of unknown reasons treated with methylprednisolone.  Respiratory failure (type I) treated with pulsed methylprednisolone and nasal continuous positive airway pressure. | Cough, fatigue, dyspnea, kidney failure  PAH severe |
| Losito et al. 2012 | 1 | 14 | 14 | M | Caucasian | N/A | Transient episode of fever, vomiting, diarrhea, followed by asthenia, all occurring in the same year | Fatigue, anemia, abnormal urinalysis (microscopic hematuria, proteinuria and hyaline-granular casts), hypertension  4 years later → PAH |
| Mitchell et al. 1986 | 1 | 4.5 | 4.5 | M | Portuguese | Non consanguinity  27 months old sister developed convulsions and hemiparesis and died  11 months old brother developed lethargy and coma and died  Another sister is normal | Growth and development were normal until 4 years, except for moderate language delay and frequent temper tantrums. | Fatigue, anorexia, increasing somnolence, hyperirritability, spasticity, delirium |
| Navarro et al. 2018 | 1 | 16 | 19 | F | White | N/A | Depression and learning difficulties | Nausea, macrocytic anemia, fatigue, abdominal pain, generally feeling unwell, hypertensive, neuropsychiatric symptoms (compulsive behavior and visual hallucinations, ataxic gait, drooling, and extreme somnolence)  TMA |
| Nogueira et al. 2017 | 1 | >1 | 15 | F | Portugal | N/A | N/A | N/A |
|  | 2 | >1 | 9 | M | Portugal | N/A | N/A | N/A |
|  | 3 | >1 | 16 | M | Portugal | N/A | N/A | N/A |
|  | 4 | >1 | 16 | M | Portugal | N/A | N/A | N/A |
|  | 5 | >1 | 4 | F | Portugal | N/A | N/A | N/A |
| Petropoulos et al. 2018 | 1 | 14 | 20 | F | Jewish descent | Non consanguinity.  Uneventful family history | Hypothyroidism and ovarian cysts | Severe hypertensive, hypertensive retinopathy with retinal hemorrhages and bilateral retinal detachment  Acute renal failure, thrombocytopenia and microangiopathic hemolytic anemia. |
| Philipponnet et al. 2020 | 1 | 19 | 19 | F | N/A | Mutations inherited from parents | Uneventful medical history | Asthenia and dyspnea, hypertension, kidney failure, severe neurologic impairment, TMA, anti-factor H antibody-associated HUS |
| Pollini et al. 2020 | 1 | 14 | 17 | M | N/A | Non consanguinity.  Mutations inherited from parents  Father's first cousin: multiple sclerosis | At birth anophthalmia of the right eye was detected and brain imaging showed a congenital arachnoid cyst in the left temporal lobe (pregnancy complicated by rubeola infection) | Learning difficulties, loss of strength in lower limbs, sudden visual loss, spastic paraparesis |
| Powers et al. 2001 | 1 | 32 | 32 | M | Italian-French-Irish descent | Case 1 and 2 were siblings  Father with Grave’s disease, mother with hypothyroidism.  A younger brother, age 39, had insulin-dependent diabetes mellitus but no neurological symptoms. | Uneventful medical history | Extremities numbness/weakness, incontinence, Lhermitte’s sign, waddling/scissoring gait, gradually quadriplegia |
|  | 2 | N/A | 44 | M | Italian-French-Irish descent | Case 1 and 2 were siblings  Father with Grave’s disease, mother with hypothyroidism.  A younger brother, age 39, had insulin-dependent diabetes mellitus but no neurological symptoms. | Hypoadrenalism and hypothyroidism, diagnosed as Schmidt’s syndrome, since age 16. | Impairments of calculation/speech, abnormal gait, inability to feed himself, deep venous thrombosis with pulmonary embolism |
| Profitlich et al. 2009 | 1 | N/A | 3 | F | Italian-American | N/A | N/A | Developmental delay, nystagmus, strabismus, retinopathy |
| Rahmandar et al. 2014 | 1 | 13 | 13 | F | N/A | Uneventful family history | Recent candidal vulvovaginitis, urinary tract infections, musculoskeletal pain.  History of sexual abuse and forced use of unknown illicit substances. | Visual hallucinations, disturbed sleep, confusion, anorexia, and difficulty with activities of daily living, seizure, ataxia |
| Roze et al. 2003 | 1 | 22 | 25.5 | F | N/A | Non consanguinity.  Case 1 and 2 were siblings | Uneventful medical history | Subacute myelopathy  Progressive gait disorder with frequent falls, hyperreflexia, bilateral proximal weakness, spasticity, impaired proprioception of lower extremities |
|  | 2 | 16 | 16 | F | N/A | Non consanguinity.  Case 1 and 2 were siblings | Uneventful medical history | Psychosis, subacute myelopathy, peripheral neuropathy, deep venous thrombosis, progressive motor disease with respiratory failure, unsteady gait,  Urinary incontinence, areflexic paraparesis, dissociative symptoms with delusions and hallucinations, impaired vibration, and position sense |
| Shinnar et al. 1984 | 1 | 14 | 14 | F | Asiatic-Indian | Case 1 and 2 were siblings | Uneventful medical history | Apathetic, slow in performing daily activities, unsteady gait |
|  | 2 | 12 | 12 | F | Asiatic-Indian | Case 1 and 2 were siblings | N/A | Asymptomatic |
| Thauvin-Robinet et al. 2007 | 1 | 18 | 39 | F | N/A | Uneventful family history | Thromboembolic disease  Encephalopathy  Myoclonia  Seizures  Myelopathy  Psychiatric disturbances  Microangiopathic nephropathy  Optic pallor | Acute mental confusion, severe and diffuse myoclonus, visual hallucinations, dysarthria, dysmetria, adiadochokinesia and tetraparesis. |
|  | 2 | 41 | 42 | M | N/A | Uneventful family history | Depression | Thromboembolic disease  Encephalopathy  Myelopathy  Psychiatric disturbances |
|  | 3 | 33 | 40 | F | N/A | Uneventful family history | Thromboembolic disease  Glomerulopathy | Recurrent venous thrombosis |
| Tsai et al. 2007 | 1 | Teens | 36 | F | Hispanic | Non consanguinity  A half-brother and a maternal cousin with schizophrenia. | Obesity  Hysterectomy  Chronic anemia  Unilateral hearing loss  Frequent urinary Tract infections due to a urogenital fistula  Adult-onset bilateral cataracts with surgical removal of the left eye cataract  Depression and psychosis | Muscular weakness  Paraesthesia of the legs Difficulty ambulating  Hemiplegia due to spinal infarct |
| Van Hove et al. 2002 | 1 | 11.9 | 12.7 | M | N/A | Case 1 and 2 were siblings | N/A | Proteinuria and hematuria, severe hypertensive encephalopathy with coma, convulsions, chronic hemolytic anemia  Thrombotic microangiopathy |
|  | 2 | 4 | 7 | F | N/A | Case 1 and 2 were siblings | Excellent student in the third grade | Proteinuria, hypertension, and chronic hemolytic anemia  Thrombotic microangiopathy |
| Wang et al. 2012 | 1 | 22 | 22 | F | N/A | Uneventful family history | Uneventful personal history | Sluggish responses and weakness in both legs during late stages of pregnancy |
|  | 2 | 40 | 40 | M | N/A | Uneventful family history | N/A | Progressive cognitive impairment, gait instability, bilateral upper and lower limb rigidity, urine incontinence, delirium, auditory hallucinations |
|  | 3 | 7 | 18 | F | N/A | Uneventful family history  Mutations inherited from parents | Difficulty walking and incontinence since the age of 7 years | Dysphasia, cognitive impairment, epilepsy |
| Wang et al. 2018 | 1 | 14 | 15 | F | China | Case 1 and 2 were siblings | N/A | Lower limb weakness, abnormal gait |
|  | 2 | 2 | 3 | F | China | Case 1 and 2 were siblings | N/A | N/A |
|  | 3 | 18 | 19 | F | China | Case 3 and 4 were siblings | N/A | Lower limb weakness, abnormal gait, psychiatric symptoms |
|  | 4 | 13 | 14 | F | China | Case 3 and 4 were siblings | N/A | Mild cognitive impairment |
|  | 5 | 7 | 18 | M | China | Case 5 and 6 were siblings | N/A | Lower limb numbness, nephropathy, psychiatric symptoms |
|  | 6 | 19 | 20 | F | China | Case 5 and 6 were siblings | N/A | Psychiatric symptoms, cognitive, impairment, nephropathy, megaloblastic anemia |
|  | 7 | 14 | 24 | F | China | Case 7 and 8 were siblings | N/A | Psychiatric symptoms, pulmonary embolism, phlebothrombosis, megaloblastic anemia |
|  | 8 | 6 | 19 | F | China | Case 7 and 8 were siblings | N/A | Mild cognitive impairment |
| Wang et al. 2019 (1) | 1 | N/A | 1 | F | China | Mutations inherited from parents | N/A | Development delay, mental retardation |
|  | 2 | N/A | 5 | M | China | Mutations inherited from parents | N/A | Developmental delay, failure to thrive |
|  | 3 | N/A | 1 | F | China | Mutations inherited from parents | N/A | Convulsions, pneumonia, diarrhea, hyponatremia, hyperkalemia, metabolic acidosis, patent foramen ovale, congenital laryngeal wheezing, bilateral hearing loss |
|  | 4 | N/A | 9 | M | China | N/A | N/A | Epilepsy, lethargy, mental retardation |
|  | 5 | N/A | 2 | F | China | Mutations inherited from parents | N/A | Pneumonia, granulocytopenia |
|  | 6 | N/A | 4 | M | China | N/A | N/A | Pneumonia, diarrhea, epilepsy |
|  | 7 | N/A | 16 | M | China | Mutations inherited from parents | N/A | Epilepsy, diarrhea |
|  | 8 | N/A | 3 | F | China | Mutations inherited from parents | N/A | N/A |
|  | 9 | N/A | 15 | F | China | Mutations inherited from parents | N/A | N/A |
|  | 10 | N/A | 8 | M | China | Mutations inherited from parents | N/A | Encephalopathy, hepatic dysfunction |
|  | 11 | N/A | 9 | M | China | Mutations inherited from parents | N/A | Epilepsy, dyskinesia, hypophrenia, joint contracture |
|  | 12 | N/A | 6 | F | China | Mutations inherited from parents | N/A | Fever, vomiting, hematuria, hyponatremia |
|  | 13 | N/A | 2 | M | China | Mutations inherited from parents | N/A | N/A |
|  | 14 | N/A | 6 | M | China | Mutations inherited from parents | N/A | Encephalopathy, tracheitis |
| Wang et al. 2019 (2) | 1 | 9 | 10 | M | China | N/A | N/A | Abnormal gait, anemia |
|  | 2 | 14 | 14 | F | China | N/A | N/A | Limb weakness, abnormal gait |
|  | 3 | 13 | 13 | M | China | N/A | N/A | Limb weakness |
|  | 4 | 15 | 16 | M | China | N/A | N/A | Psychiatric changes, seizures, abnormal gait |
|  | 5 | 18 | 19 | F | China | N/A | N/A | Psychiatric changes, cognitive deficit, abnormal gait |
|  | 6 | 20 | 22 | F | China | N/A | N/A | Psychiatric changes |
|  | 7 | 24 | 31 | F | China | N/A | N/A | Psychiatric changes, limb weakness |
|  | 8 | 13 | 15 | F | China | N/A | N/A | Limb weakness |
|  | 9 | 16 | 16 | F | China | N/A | N/A | Psychiatric changes, abnormal gait |
|  | 10 | 4 | 5 | F | China | N/A | N/A | Limb weakness, seizures, anemia |
|  | 11 | 7 | 11 | F | China | N/A | N/A | Cognitive deficit, limb weakness, seizures |
|  | 12 | 14 | 14 | F | China | N/A | N/A | Cognitive deficit, abnormal gait |
|  | 13 | 7 | 18 | M | China | N/A | N/A | Psychiatric changes, cognitive deficit, renal dysfunction, anemia |
|  | 14 | 19 | 20 | F | China | N/A | N/A | Psychiatric changes, cognitive deficit, renal dysfunction, anemia |
|  | 15 | 12 | 14 | M | China | N/A | N/A | Cognitive deficit, seizures, anemia |
|  | 16 | 14 | 24 | F | China | N/A | N/A | Psychiatric changes, pulmonary embolism, anemia |
|  | 17 | 12 | 16 | M | China | N/A | N/A | Limb weakness, seizures, abnormal gait |
|  | 18 | 10 | 14 | M | China | N/A | N/A | Limb weakness |
|  | 19 | 38 | 39 | M | China | N/A | N/A | Limb weakness, abnormal gait |
|  | 20 | 15 | 15 | F | China | N/A | N/A | Psychiatric changes, limb weakness |
|  | 21 | 29 | 29 | M | China | N/A | N/A | Abnormal gait |
|  | 22 | 18 | 20 | M | China | N/A | N/A | Psychiatric changes, limb weakness |
|  | 23 | 19 | 29 | M | China | N/A | N/A | Cognitive deficit, limb weakness, abnormal gait |
|  | 24 | 17 | 22 | F | China | N/A | N/A | Limb weakness, abnormal gait, anemia |
|  | 25 | 18 | 18 | F | China | N/A | N/A | Psychiatric changes, seizures |
|  | 26 | 29 | 29 | M | China | N/A | N/A | Psychiatric changes, seizures |
| Wang et al. 2019 (3) | 1 | 14 | 14 | F | China | N/A | N/A | Cognitive impairment, irritability, psychosis, moderate memory decline, paraplegia, bilateral pyramidal tract signs |
|  | 2 | 14 | 14 | M | China | N/A | N/A | Cognitive impairment, apathy, lethargy, depression, severe cognitive impairment, deterioration in school performance, bilateral pyramidal tract signs |
|  | 3 | 40 | 40 | M | China | N/A | N/A | Cognitive impairment, moderate memory impairment, quadriplegia, bilateral pyramidal tract signs |
|  | 4 | 22 | 22 | F | China | N/A | N/A | Cognitive impairment, moderate impaired memory and calculation ability, paraplegia, bilateral pyramidal tract signs |
|  | 5 | 11 | 18 | F | China | N/A | N/A | Gait disturbance, mild memory impairment, progressive spastic paraplegia, bilateral pyramidal tract signs |
|  | 6 | 13 | 13 | F | China | N/A | N/A | Cognitive impairment, irritability, aggressiveness, moderate cognitive impairment, deterioration in school performance, mild paraplegia, bilateral pyramidal tract signs |
|  | 7 | 26 | 26 | M | China | N/A | N/A | Weakness of lower limbs, generalized tonic-clonic seizures, progressive spastic paraplegia, bilateral pyramidal tract signs |
|  | 8 | 16 | 16 | M | China | N/A | N/A | Weakness of lower limbs, generalized tonic-clonic seizures, paraplegia, bilateral pyramidal tract signs |
|  | 9 | 30.5 | 32 | M | China | Case 9 and 10 were siblings. | N/A | Psychiatric symptoms, euphoria, agitation, auditory and vision hallucinations, aggressiveness, mild memory impairment, paraplegia, bilateral pyramidal tract signs |
|  | 10 | 29 | 29 | M | China | Case 9 and 10 were siblings. | N/A | Psychiatric symptoms, euphoria, agitation, irritability, aggressiveness, mild memory impairment, paraplegia, bilateral pyramidal tract signs |
|  | 11 | 14 | 15 | F | China | N/A | N/A | Weakness of lower limbs, mild memory impairment, progressive spastic paraplegia, bilateral pyramidal tract signs |
|  | 12 | 23 | 23 | M | China | N/A | N/A | Weakness of lower limbs, mild memory impairment, progressive spastic paraplegia, bilateral pyramidal tract signs |
|  | 13 | 15 | 15 | M | China | N/A | N/A | Cognitive impairment, impaired memory and calculation ability, mild paraplegia, bilateral pyramidal tract signs |
|  | 14 | 18 | 29 | M | China | N/A | N/A | Cognitive impairment, moderate impaired memory, speech difficulties, paraplegia, bilateral pyramidal tract signs |
|  | 15 | 14 | 20 | M | China | N/A | N/A | Epilepsy, generalized tonic-clonic seizures, bilateral pyramidal tract signs, thrombosis of peroneal vein and intramuscular vein of left lower limb |
|  | 16 | 14 | 24 | F | China | N/A | N/A | Decreased vision, moderate depression, mild memory impairment, generalized tonic-clonic seizures, paraplegia, bilateral pyramidal tract signs |
| Wang et al. 2019 (4) | 1 | 19 | 19 | F | N/A | N/A | N/A | Declined cognitive ability, change in posture, strange behaviour |
| Wei et al. 2019 | 1 | 7 | 10 | M | China | N/A | N/A | Decline performance |
|  | 2 | 16 | 16 | M | China | N/A | N/A | Spastic paraplegia |
|  | 3 | 13 | 17 | M | China | N/A | N/A | Spastic paraplegia |
|  | 4 | 10 | 15 | F | China | N/A | N/A | Depression |
|  | 5 | 24 | 24 | M | China | N/A | N/A | Spastic paraplegia |
|  | 6 | 13 | 13 | M | China | N/A | N/A | Spastic paraplegia |
|  | 7 | 7 | 12 | M | China | N/A | N/A | Decline performance |
|  | 8 | 26 | 26 | M | China | N/A | N/A | Cognitive impairment |
| Wei et al. 2020 | 1 | 31 | 31 | M | N/A | Uneventful family history  Mutations inherited from parents | Milder lower extremity weakness for 1 month at age 13 years with previous memory impairment | Lower limb weakness |
| Wen et al. 2020 | 1 | 9 | 12 | F | China | Uneventful family history | Uneventful medical history | Pulmonary hypertension |
| Wu et al. 2017 | 1 | 32 | 33 | M | China | Case 1 and 2 were siblings.  Uneventful family history  Non consanguinity. | Uneventful medical history | Limb weakness, difficulty walking, blurred vision  Manic-depressive psychosis |
|  | 2 | 29 | 29 | M | China | Case 1 and 2 were siblings.  Uneventful family history  Non consanguinity.  Mutations inherited from parents | Uneventful medical history | Limb weakness, difficulty walking, bilateral  visual decline, cognitive impairment  Manic-depressive psychosis |
| Wu et al. 2018 | 1 | 45 | 45 | M | China | Case 1, 2 and 3 were siblings.  Uneventful family history | N/A | Psychiatric symptoms  Spastic paresis of lower limbs |
|  | 2 | N/A | N/A | F | China | Case 1, 2 and 3 were siblings.  Uneventful family history | N/A | Asymptomatic |
|  | 3 | N/A | N/A | F | China | Case 1, 2 and 3 were siblings.  Uneventful family history | N/A | Asymptomatic |
| Zhao et al. 2021 | 1 | 2 | 25 | M | N/A | Non consanguinity.  Uneventful family history | IgA nephropathy | Pulmonary hypertension, haemolytic anaemia |
|  | Total: 199 patients | Mean age: 15.29+9.22 years | Mean age at diagnosis: 17.62+10.82 years | F: 94  M: 92  N/A: 13 | Asian origin:109 patients  Caucasian origin: 33 patients  Hispanic origin: 6 patients  African origin: 3 patients    American origin: 2 patients | History of non-consanguinity: 27 patients  History of consanguinity: 14 patients  Siblings: 53 patients  Uneventful family history: 28 patients | Symptoms of intellectual disability, learning difficulties and delayed learning, memory abilities and decreased locomotor activities during childhood: 29 patients  Psychiatric disorders: 6 patients  Anemia: 6 patients  Respiratory disorders: 6 patients  Kidney disease: 5 patients  Seizures: 5 patients  Gait disorders: 4 patients  Thrombotic microangiopathy: 4 patients  Thrombotic episodes: 3 patients.  Uneventful medical history: 25 patients  N/A: 126 patients. | Neuropathy/myelopathy: 94 patients.  Encephalopathy: 80 patients.  Psychiatric symptoms: 57 patients.  Thrombotic microangiopathy: 38 patients.  Seizures: 28 patients.  Pulmonary hypertension: 20 patients.  Thrombotic phenomena: 15 patients.  Megaloblastic anaemia: 13 patients.  Other symptoms: anorexia or weight loss, ocular pathology, diarrhoea, pneumonia, liver failure, nausea, vomiting, and developmental delay and failure to thrive (in the paediatric age).  Asymptomatic: 7 patients  N/A: 11 patients |
| **Notes:**  **^1^** Ethnicity taken from Morel et al. 2006  **^2^** Additional data on Patient 1 and data on Patient 2 were taken from Lemoine et al. 2018  **^3^** Additional data on Patient 1 and Patient 2 were taken from Motte et al. 2019  **Abbreviations:** F: female, M: male, N/A: Not available, PAH: pulmonary arterial hypertension, TMA: renal thrombotic microangiopathy, HUS: hemolytic uremic syndrome | | | | | | | | |

**Table 6. Biochemical diagnosis**

| **Author and year** | **# of cases** | **Biochemical diagnosis** | | | | |
| --- | --- | --- | --- | --- | --- | --- |
|  |  | **MMA** | **Homocysteine** | **Methionine** | **C3 and C3/C2** | **Vitamin B12 and folic acid** |
| Ardissino et al. 2017 | 1 | Urine: 20 mMol/mol (N: <2) | 364 mMol/L (N: <15.4) | 8 uMol/L (N: 15-20) | N/A | N/A |
| Augoustides-Savvopoulou et al. 1999 | 1 | Urine: 558 mmol/mol creatinine (N: <2) | Plasma: 225 μmol/L (N: 4-11) | 4 μmol/L (N: 11-43) | N/A | Normal levels |
| Backe et al. 2013 | 1 | Serum: 118 μmol/L (N: 0.07-0.30)  Urine: increased | Serum: 158–176 μmol/L (N: 6-16)  Urine: 31 μmol/mmol (N: <1.1) | 6 μmol/L (N: 14-39) | 11.2 μmol/L (N: 0.18–0.80)  N/A | Normal levels of vitamin B12  Elevated levels of folic acid |
| Ben-Omran et al. 2007 | 1 | Urine: 3240 mmol/ mol creatinine (N: <10) | Free Hcy: 15 μmol/L (N: <5) | 5 μmol/L (N: 6-39) | N/A | Normal levels |
|  | 2 | Urine: 5558 mmol/ mol creatinine (N: <10) | Plasma: 184 μmol/L (N: 5-15) | 11 μmol/L (N: 6-39) | N/A | Normal levels of vitamin B12 |
| Bodamer et al. 2001 | 1 | 1722 mmol/mol creatinine (N: <2) | Plasma: 27.9 μmol/L (N: 5.4-16.2) | N/A | N/A | Normal levels of vitamin B12 |
| Boxer et al. 2005 | 1 | 41.3 μΜ (N: <0.3) (after starting treatment) | Serum: 308.9 μM (N: 6-16) | 12 μM (N: 10-42) | N/A | Normal levels |
|  | 2 | N/A | N/A | N/A | N/A | N/A |
| Brox-Torrecilla et al. 2021 | 1 | Plasma: 117.18 µmol/L (N: 0.08-0.56) | Serum: 45.4 µmol/L (N: 0.2 ±  0.4) | N/A | N/A | Elevated levels |
| Brunelli et al. 2002 | 1 | 53087 nmol/l (N: 73-271)  Urine: 1100 mg/mg creatinine (N: <10) | 134 µmol/l (N: N/A)  Free Hcy: 21.1 nmol/ml (N: undetectable) | 7.3 nmol/ml (N: 8-49) | C3 73.2 mg/dl (N: 71.0–  150.0) | N/A |
| Chang et al. 2020 | 1 | Urine: 332.9 mmol/mol creatinine (N: 0.2–3.6) | Serum: 154.5 μmol/L (N: 5–15) | N/A | N/A | Normal levels |
|  | 2 | Urine: 175.2 mmol/mol creatinine (N: 0.2-3.6) | Serum: 103.2 μmol/L (N: 5–15) | N/A | N/A | Normal levels |
|  | 3 | Urine: 142.4 mmol/mol creatinine (N: 0.2–3.6) | Serum: 97.3 μmol/L (N: 5–15) | N/A | N/A | Normal levels |
|  | 4 | Urine: 220.1 mmol/mol creatinine (N: 0.2–3.6) | Serum: 136.5 μmol/L (N: 5-15) | N/A | N/A | Normal levels |
|  | 5 | Urine: 263.1 mmol/mol creatinine (N: 0.2–3.6) | Serum: 59.2 μmol/L (N: 5-15) | N/A | N/A | Normal levels |
| Chu et al. 2020 | 1 | Urine: 1012.83 µg (N: 0.2–3.6) | Plasma: 93.20 µmol/L (N: 6–17) | N/A | 2.73 µmol/L (N: 1.00-4.00)  1 (N: 0.03-0.50) | N/A |
|  | 2 | Urine: 467.25 µg (N: 0.2–3.6) | Plasma: 100.22 µmol/L (N: 6–17) | N/A | 2.79 µmol/L (N: 1.00-4.00)  0.85 (N: 0.03-0.50) | N/A |
|  | 3 | Urine: 64.70 µg (N: 0.2–3.6) | Plasma: 111.88 µmol/L (N: 6–17) | N/A | N/A | N/A |
|  | 4 | Urine: 73.12 µg (N: 0.2–3.6) | Plasma: 129.04 µmol/L (N: 6–17) | N/A | 4.45 µmol/L (N: 1.00-4.00)  0.39 (N: 0.03–0.50) | N/A |
|  | 5 | Urine: 1213.92 µg (N: 0.2–3.6) | Plasma: 230.97 µmol/L (N: 6–17) | N/A | 6.81 µmol/L (N: 1.00-4.00)  0.78 (N: 0.03–0.50) | N/A |
|  | 6 | Urine: 687.99 µg (N: 0.2–3.6) | Plasma: 219.72 µmol/L (N: 6–17) | N/A | 5.12 µmol/L (N: 1.00-4.00)  0.49 (N: 0.03–0.50) | N/A |
|  | 7 | Urine: 190.10 µg (N: 0.2–3.6) | Plasma: 72.37 µmol/L (N: 6–17) | N/A | 4.17 µmol/L (N: 1.00-4.00)  0.85 (N: 0.03–0.50) | N/A |
|  | 8 | Urine: 41.66 µg (N: 0.2–3.6) | Plasma: 86.56 µmol/L (N: 6–17) | N/A | 4.70 µmol/L (N: 1.00-4.00)  0.18 (N: 0.03–0.50) | N/A |
| Collison et al. 2015 | 1 | Urine: 510 mmol/mol creatinine (N: 0-3.60) | Urine: 51 mg/day (N: 0–32) | N/A | N/A | N/A |
| Cornec-Legall et al. 2014 | 1 | Urine: 244 mmol per mole of creatinine (N: < 10 mmol/mol) | 25.1 mg/L (N: 0.5-1.9) | Plasma: 0.06 mg/dL (N: 0.12-0.63) | N/A | Normal levels |
| Cui et al. 2019 | 1 | Urine: elevated | Serum: 133.4 µmol/L (N: 0-15) | N/A | Elevated C3/C2 levels | Normal levels |
|  | 2 | N/A | N/A | N/A | N/A | N/A |
|  | 3 | N/A | N/A | N/A | N/A | N/A |
|  | 4 | Urine: 216.7 (N: 0-4) | Serum: 44.552 µmol/L (N: 0–15) | 20.80 µmol/L (N: 8–50) | 10.15 µmol/L (N: 0.3–5)  1.05/1 (N: 0.02–0.25) | Normal levels |
|  | 5 | N/A | N/A | N/A | N/A | N/A |
| Davin et al. 2009 | 1 | N/A | Plasma: 185 µg/L (N: N/A) | N/A | N/A | N/A |
| Gerth et al. 2008 | 1 | N/A | N/A | N/A | N/A | N/A |
|  | 2 | N/A | N/A | N/A | N/A | N/A |
|  | 3 | N/A | N/A | N/A | N/A | N/A |
|  | 4 | N/A | N/A | N/A | N/A | N/A |
| Gilson et al. 2018 | 1 | Serum: 53.93 μmol/L (N: 0-0.4) | 81.7 mcmol/L (N: 3.7-13.9) | N/A | N/A | Normal levels of vitamin B12 |
| Gold et al. 1996 **^1^** | 1 | Urine: 2900 mmol/mol creatinine (N:< 2) | Plasma: 174 µmol/L (N: <15) | 7 µmol/L (N: 13-28) | N/A | Normal levels |
|  | 2 | Urine: 1550 mmol/mol creatinine (N: <2) | Plasma: 79 µmol/L (N: <15) | 8 µmol/L (N: 13-28) | N/A | N/A |
| Goodman et al. 1970 | 1 | Urine: 180-2100 mg (N: N/A) | Urine: 6.1-87.0 µmol | Plasma: 0.02 μmoles/ml (N: 0.01-0.05) | N/A | Normal levels |
|  | 2 | Urine: 150-690 mg | N/A | Plasma: 0.02 μmoles/ml (N: 0.01-0.05) | N/A | Normal levels |
| Grandone et al. 2019 | 1 | 1.09 μmol/L (N: 0-0.7) | N/A | N/A | N/A | Normal levels |
| Grangé et al. 2015 **^2^** | 1 | Elevated | 73 μmol/l (N: N/A) | 17 μmol/l (N: N/A) | Elevated C3 levels | 558 pg/ml |
|  | 2 | N/A | N/A | N/A | N/A | N/A |
| Guigonis et al. 2005 | 1 | Urine: 19 μmol/mmol creatinine (N: 1-10.3) | Plasma: 14.6 mg/L (N: 0.73-2.31) | Plasma: 0.5 mg/dL (N: 0.3-0.4) | N/A | Normal levels |
|  | 2 | Urine: 22.5 μmol/mmol creatinine (N: 1-10.3) | Plasma: 6.12 mg/L (N: 0.73-2.31) | Plasma: 0.5 mg/dL (N: 0.3-0.4) | N/A | Normal levels |
| Gündüz et al. 2014 | 1 | Urine: 980 mmol/mmol creatinine (N: <130) | 39.3 mmol/L (N: 3.3-8.3) | 10.5 μmol/L (N: 9-44) | N/A | Normal levels |
| Gurkas et al. 2015 | 1 | Urine: > 250 μmol/mmol creatinine (N: not detectable) | Plasma: 196.6 μmol/L (N: 5-14) | Plasma: 7 μmol/L (N: 31-83) | N/A | Normal levels |
| Heil et al. 2007 | 1 | Urine: 3697 mmol/mol creatinine (N: <20)  Plasma: 140 μmol/L (N: <0.32) | Plasma: 179.2 μmol/L (N: <9.9) | Plasma: 4 μmol/L (N: >10) | N/A | Normal levels |
|  | 2 | Urine: 2977 mmol/mol creatinine (N: <20)  Plasma: 120 μmol/L (N: <0.32) | Plasma: 119.6 μmol/L (N: <9.9) | Plasma: 10 μmol/L (N: >10) | N/A | N/A |
| Higashimoto et al. | 1 | Serum: 165.8 μmol/L (N: 0.045-0.325) | Serum: 201.9 μmol/L (N: 0-12.2) | Plasma: 20 μmol/L (N: 16-34) | N/A | Normal levels of vitamin B12  Elevated levels of folic acid |
|  | 2 | Serum: 28.5 μmol/L (N 0.045-0.325) | Serum: 162.98 μmol/L (N: 0-12.2) | N/A | N/A | Normal levels of vitamin B12 |
|  | 3 | Serum: 60.7 μmol/L (N 0.045-0.325) | Serum: 215 μmol/L (N: 0-12.2) | N/A | N/A | Normal levels of vitamin B12 |
| Huemer et al. 2014 | 1 | N/A | 353 μmol/L (N: 5-15) | 16 μmol/L (N: N/A) | N/A | N/A |
|  | 2 | Urine: elevated | 228–264 μmol/L (N: N/A) | 7.7 μmol/L (N: N/A) | N/A | Normal levels of vitamin B12 |
|  | 3 | Urine: 1168 mmol/mol creatinine (N: N/A) | Plasma: 53.3 μmol/L (N: N/A) | 9.6 μmol/L (N: N/A) | N/A | Normal levels of vitamin B12 |
| Iodice et al. 2013 | 1 | Urine: elevated | Plasma: 66.9 μmol/L (N: 4-19) | 11 μmol/L (N: 11-50) | N/A | Normal levels |
|  | 2 | N/A | N/A | N/A | N/A | N/A |
| Jiménez-Varo et al. 2015 | 1 | Urine: elevated | 12 μmol/L (N: <1)  Urine: 344.9 μmol/L (N <16 μmol/L) | 0 | N/A | N/A |
| Kılıç et al. 2013 | 1 | Urine: 2080 µmol/mmol creatinine (N: not detectable) | 232 µmol/L (N: 5.5–17) | 5.4 µmol/L (N: 43-223) | N/A | Normal levels |
| Koenig et al. 2015 | 1 | Urine: normal levels | Serum: 140.7 µmol/l (N: <10) | 20 µmol/l (N: 20-40) | N/A | Normal levels |
| Kömhoff et al. 2013 | 1 | N/A | N/A | N/A | N/A | Elevated levels of vitamin B12 |
|  | 2 | 14 424 nmol/L (N: 90-340) | 123 µmol/l (N: 4-12) | N/A | N/A | Elevated levels of vitamin B12 |
|  | 3 | 1546 nmol/L (N: 90-340) | 185 µmol/l (N: 4-12) | N/A | N/A | N/A |
|  | 4 | 8602 nmol/L (N: 90-340) | 142 µmol/l (N: 4-12) | N/A | N/A | Elevated levels of vitamin B12 |
|  | 5 | N/A | 147 µmol/l (N: 4-12) | N/A | N/A | Elevated levels of vitamin B12 |
| Lemoine et al. 2018 | 1 | N/A | 73 µmol/l (N: <13) | 17 µmol/l (N: 11-37) | N/A | Vitamin B12 558 pg/ml |
|  | 2 | N/A | N/A | N/A | N/A | N/A |
|  | 3 | Urine: 244 µmol/mmol (N:<4) | 185 µmol/L (N: <13) | 4 µmol/L (N: 11-37) | N/A | Vitamin B12 500 pg/ml |
|  | 4 | Urine: 19 µmol/mmol (N:<4) | 104 µmol/L (N: <13) | 30 µmol/L (N: 11-37) | N/A | Vitamin B12 600 pg/ml |
|  | 5 | Urine: 22.5 µmol/mmol (N:<4) | 44 µmol/L (N: <13) | 30 µmol/L (N: 11-37) | N/A | Vitamin B12 550 pg/ml |
|  | 6 | Urine: 91 µmol/mmol (N:<4) | 97 µmol/L (N: <13) | 40 µmol/L (N: 11-37) | N/A | Vitamin B12 580 pg/ml |
|  | 7 | Urine: 83 µmol/mmol (N:<4) | 230 µmol/L (N: <13) | N/A | N/A | Vitamina B12 1082 pg/ml |
| Li et al. 2015 | 1 | Urine: 0.412 mmol/creatinine (N: <0.001) | 97.58 mmol/L (N: 1.90-12.98) | N/A | N/A | 1200 pg/mL (N: 140.0-960.0) |
|  | 2 | Urine: 2.804 mmol/creatinine (N: <0.001) | 150.38 mmol/L (N: 1.90-12.98) | N/A | N/A | 557 pg/mL (N: 140.0-960.0) |
|  | 3 | Urine: 0.550 mmol/creatinine (N: <0.001) | 134.15 mmol/L (N: 1.90-12.98) | N/A | N/A | 1500 pg/mL (N: 140.0-960.0) |
| Lin et al. 2009 | 1 | 58 360 nmol/L (N: 87-318)  Urine: 3600 mmol/mol creatinine (N: N/A) | 147 µmol/L (N: 5.4-11.90) | N/A | N/A | Serum:  Free carnitine 1 µmol/L (N 19-48)  Esterified carnitine 1 |
| Liu et al. 2015 (1) | 1 | Urine: increased by 500.5-fold | Serum: >50 μM (N: 4–20) | Normal levels | N/A | Normal levels |
|  | 2 | Urine: increased by 3619.8-fold | Serum: >50 μM (N: 4–20) | Normal levels | N/A | Normal levels |
|  | 3 | Urine: increased by 463.7-fold | Serum: >50 μM (N: 4–20) | Normal levels | N/A | Normal levels |
|  | 4 | Urine: increased by 133.3-fold | Serum: >50 μM (N: 4–20) | Normal levels | N/A | Normal levels |
|  | 5 | Urine: increased by 13 831.8-fold | Serum: 631.4 μM (N: 4–20) | Normal levels | N/A | Normal levels |
| Liu et al. 2015 (2) | 1 | Urine MMA: 92.66 μg/mg creatinine (N: 0.2–3.6) | Plasma tHcy: >50 μmol/L (N: 5–15) | 7.05 μmol/L (N: 10 -50) | 7.90 μmol/L (N: 1-5)  1.06 (N: 0.03-0.25) | Normal levels |
| Liu et al. 2017 | 1 | Serum: 0.218 mg/dL (N: N/A)  Urine: 0.428 mg/dL (>420 times the reference value) | Plasma: >50.0 μmol/L (N: 5-15) | 7.6 μmol/L (N: 8.6 -23.3) | Elevated C3 and C3/C2 levels | Normal levels |
|  | 2 | Serum: 0.294 mg/dL (N: N/A)  Urine: 0.354 mg/dL (>350 times the reference value) | Plasma: >50.0 μmol/L (N: 5-15) | 22.6 μmol/L (N: 8.6 -23.3) | Elevated C3 and C3/C2 levels | Normal levels |
|  | 3 | Serum: 0.299 mg/dL (N: N/A)  Urine: 0.127 mg/dL (>120 times the reference value) | Plasma: >50.0 μmol/L (N: 5-15) | 8.4 μmol/L (N: 8.6 -23.3) | Elevated C3 and C3/C2 levels | Normal levels |
|  | 4 | Serum: 0.383 mg/dL (N: N/A)  Urine: 0.1034 mg/dL (>103 times the reference value) | Plasma: 193.76 μmol/L (N: 5-15) | 11.1 μmol/L (N: 8.6 -23.3) | Elevated C3 and C3/C2 levels | Normal levels |
| Liu et al. 2020 | 1 | Elevated levels | Plasma: 108 μmol/L (N: N/A) | N/A | Elevated C3 and C3/C2 | Normal levels |
|  | 2 | Elevated levels | Plasma: 131 μmol/L (N: N/A) | N/A | Elevated C3 and C3/C2 | Elevated levels |
|  | 3 | Elevated levels | Plasma: 103 μmol/L (N: N/A) | N/A | Elevated C3 and C3/C2 | Normal levels |
|  | 4 | Elevated levels | Plasma: 136 μmol/L (N: N/A) | N/A | Elevated C3 and C3/C2 | Normal levels |
| Losito et al. 2012 | 1 | N/A | 147.2 μmol/L (N: 2.8-15.7) | N/A | N/A | Normal levels of vitamin B12  Low levels of folic acid |
| Mitchell et al. 1986 | 1 | 2.1 mmol/g creatinine (N: 0.035+0.010) | 12.7 μmol/L (N: undetectable) | 16.8 μmol/L (N: 10-41) | N/A | Normal levels of folic acid  Elevated levels of vitamin B12 |
| Navarro et al. 2018 | 1 | Serum: 9.3 μmol/L (N: <0.27) | Serum: 434 μmol/L (N: <20) | 6 μmol/L (N: 11-37) | N/A | N/A |
| Nogueira et al. 2017 | 1 | Urine: 539 μmol/mmol create (N: <3.3) | 66 μmol/l (N: 4-14) | N/A | N/A | N/A |
|  | 2 | Urine: 1674 μmol/mmol create (N: <3.3) | 86 μmol/l (N: 4-14) | N/A | N/A | N/A |
|  | 3 | Urine: 1560 μmol/mmol create (N: <3.3) | 66 μmol/l (N: 4-14) | N/A | N/A | N/A |
|  | 4 | Urine: 1009 μmol/mmol create (N: <3.3) | 42 μmol/l (N: 4-14) | N/A | N/A | N/A |
|  | 5 | Urine: 2420 μmol/mmol create (N: <3.3) | 72 μmol/l (N: 4-14) | N/A | N/A | N/A |
| Petropoulos et al. 2018 | 1 | Plasma: 80.15 nmol/L (100-fold over the expected level for patients with renal failure) | Plasma: 124 μmol/L (N: N/A) | N/A | N/A | Normal levels |
| Philipponnet et al. 2020 | 1 | Urine: 247 mmol/mol of creatinine (N: <4 μmol/L) | Serum: 285 μmol/L (N: 3.2-10.7) | Plasma: 12 μmol/L (N: 21-35)  Urine: <1 μmol/mmol of creatinine (N: 2-16) | N/A | Normal levels |
| Pollini et al. 2020 | 1 | 1075 mmol/ mol creatinine (N: 0.4-23) | 162 μmol/L (N: 4.7-11.3) | 23 μmol/L (N: 26-38) | N/A | N/A |
| Powers et al. 2001 | 1 | N/A | N/A | N/A | N/A | Normal levels of vitamin B12 |
|  | 2 | N/A | N/A | N/A | N/A | Normal levels of vitamin B12 |
| Profitlich et al. 2009 | 1 | Mean urine MMA 57 mmol/mol Cr | Mean Hcy 99 μmol/L (N: 5–15) | N/A | N/A | N/A |
| Rahmandar et al. 2014 | 1 | Plasma: 30.99 μmol/L (N: <0.4) after 6 months of treatment | Plasma: 178 μmol/L (N: 5-15) | Plasma: 8 μmol/L (N: 7-47) | 4.32 μmol/L (N: <1)  N/A | Normal levels |
| Roze et al. 2003 | 1 | Urine: 3330 μmol/mmol of creatinine (N: <50) | Plasma: 125 μmol/L (N: <15) | Plasma: 10 μmol/L (N: >20) | N/A | Normal levels of vitamin B12 |
|  | 2 | Urine: 3890 μmol/mmol of creatinine (N: <50) | Plasma: 205 μmol/L (N: <15) | Plasma: 7 μmol/L (N: >20) | N/A | Normal levels of vitamin B12 |
| Shinnar et al. 1984 | 1 | Urine: elevated | 46 μmol/L (N: 0) | Traces (N: 1-49 μmol/L) | N/A | Normal levels |
|  | 2 | Urine: elevated | 12 μmol/L (N: 0) | N/A | N/A | 1062 pg/ml |
| Thauvin-Robinet et al. 2007 | 1 | Urine: 1708 mmol/mol creatinine (N: <5) | Plasma: 172 μmol/L (N: 6-14) | Plasma: 7 μmol/L (N: 11-29) | N/A | N/A |
|  | 2 | Urine: 1067 mmol/mol creatinine (N: <5) | Plasma: 228 μmol/L (N: 6-14) | N/A | N/A | Normal levels of vitamin B12 |
|  | 3 | Urine: 1209 mmol/mol creatinine (N: <5) | Plasma: 288 μmol/L (N: 6-14) | Plasma: 12 μmol/L (N: 11-29) | N/A | N/A |
| Tsai et al. 2007 | 1 | 21,696 nmol/L (N: 73-271) | Serum: 57 μmol/L (N: 5.1-13.9) | Normal levels | N/A | Normal levels of vitamin B12 |
| Van Hove et al. 2002 | 1 | Urine: 27 mmol/mol creatinine (N: <2) | Plasma: 123.1 μΜ (N: 5-14) | N/A | N/A | Normal levels of folate |
|  | 2 | Urine: 10 mmol/mol creatinine (N: <2) | Plasma: 53.7 μΜ (N: 5-14) | N/A | N/A | Normal levels of folate  Elevated of vitamin B12 |
| Wang et al. 2012 | 1 | Urine: 166.64 μg/mg creatinine (N: 0.2–3.6) | Serum: 79.8 μM (N: 5.0–15.0) | N/A | N/A | Normal levels of folate  Elevated of vitamin B12 |
|  | 2 | Urine: 70.53 μg/mg creatinine (N: 0.2-3.6) | Serum: 57.2 μM (N: 5.0–15.0) | N/A | N/A | Normal levels |
|  | 3 | Urine: 94.71 μg/mg creatinine (N: 0.2-3.6) | Serum: 56.8 μM (N: 5.0–15.0) | N/A | N/A | Normal levels |
| Wang et al. 2018 | 1 | Urine: 54.5 μM/L (N: 0.2-5.6) | Serum: 69.5 μM/L (N: 0-15) | N/A | N/A | Normal levels |
|  | 2 | Urine: 26.7 μM/L (N:0.2-5.6) | Serum: 80.2 μM/L (N: 0-15) | N/A | N/A | Normal levels |
|  | 3 | Urine: 52.6 μM/L (N: 0.2-5.6) | Serum: 138 μM/L (N: 0-15) | N/A | N/A | Normal levels |
|  | 4 | Urine: 185.8 μM/L (N: 0.2-5.6) | Serum: 110 μM/L (N: 0-15) | N/A | N/A | Normal levels |
|  | 5 | Urine: 97 μM/L (N: 0.2-5.6) | Serum: 127.6 μM/L (N: 0-15) | N/A | N/A | Normal levels |
|  | 6 | Urine: 178 μM/L (N: 0.2-5.6) | Serum: 155 μM/L (N: 0-15) | N/A | N/A | Normal levels |
|  | 7 | Urine: 111.7 μM/L (N: 0.2-5.6) | Serum: 114 μM/L (N: 0-15) | N/A | N/A | Normal levels |
|  | 8 | Urine: 123.7 μM/L (N:0.2-5.6) | Serum: 96 μM/L (N: 0-15) | N/A | N/A | Normal levels |
| Wang et al. 2019 (1) | 1 | Urine: 127.62 (N: <1) | Serum: 138.80 μM (N: 0-15) | N/A | 5.97 μM (N: 0.00-3.19)  0.22 (N: 0.01–0.37) | N/A |
|  | 2 | Urine: 724.16 (N: <1) | Serum: 179.50 μM (N: 0-15) | N/A | 15.91 μM (N: 0.00–5.14)  0.97 (N: 0.02–0.29) | N/A |
|  | 3 | Urine: 90.19 (N: <1) | N/A | N/A | N/A | N/A |
|  | 4 | Urine: 169.95 (N: <1) | Serum: 141.4 μM (N: 0-15) | N/A | N/A | N/A |
|  | 5 | Urine: 108.54 (N: <1) | N/A | N/A | N/A | N/A |
|  | 6 | Urine: 29.75 (N: <1) | Serum: 66.60 μM (N: 0-15) | N/A | N/A | N/A |
|  | 7 | Urine: 1077.2 (N: <1) | Serum: 130 μM (N: 0-15) | N/A | 6.93 μM (N: 0.00–3.97)  0.41 (N: 0.02–0.30) | N/A |
|  | 8 | Urine: 1752.5 (N: <1) | Serum: 139.5 μM (N: 0-15) | N/A | 20.16 μM (N: 0.00-3.19)  0.61 (N: 0.01–0.37) | N/A |
|  | 9 | Urine: 704.25 (N: <1) | Serum: 191.10 μM (N: 0-15) | N/A | 4.21 μM (N: 0.00–3.97)  0.50 (N: 0.02–0.30) | N/A |
|  | 10 | Urine: 142.68 (N: <1) | Serum: 94.70 μM (N: 0-15) | N/A | 4.52 μM (N: 0.00–5.23)  1.07 (N: 0.02–0.23) | N/A |
|  | 11 | Urine: 386.36 (N: <1) | Serum: 79.5 μM (N: 0-15) | N/A | 8.40 μM (N: 0.00–5.23)  0.88 (N: 0.02–0.23) | N/A |
|  | 12 | Urine: 126.94 (N: <1) | Serum: 97.5 μM (N: 0-15) | N/A | 4.25 μM (N: 0.00–5.14)  0.46 (N: 0.02–0.29) | N/A |
|  | 13 | Urine: 459.92 (N: <1) | Serum: 72.9 μM (N: 0-15) | N/A | 3.22 μM (N: 0.00-3.19)  0.51 (N: 0.01–0.37) | N/A |
|  | 14 | Urine: 67.27 (N: <1) | Serum: 64.70 μM (N: 0-15) | N/A | 2.50 μM (N: 0.00–5.14)  0.17 (N: 0.02–0.29) | N/A |
| Wang et al. 2019 (2) | 1 | Urine: 89.2 μmol/L (N: 0.2-5.6) | Serum: 102 μmol/L (N: 0-15) | N/A | N/A  0.62 (N:<0.30) | Normal levels |
|  | 2 | Urine: 54.5 μmol/L (N: 0.2-5.6) | Serum: 69.5 μmol/L (N: 0-15) | N/A | N/A  0.59 (N:<0.30) | Normal levels |
|  | 3 | Urine: 198 μmol/L (N: 0.2-5.6) | Serum: 62.8 μmol/L (N: 0-15) | N/A | N/A  0.97 (N:<0.30) | Normal levels |
|  | 4 | Urine: 101.9 μmol/L (N: 0.2-5.6) | Serum: 110.1 μmol/L (N: 0-15) | N/A | N/A  0.64 (N:<0.30) | Normal levels |
|  | 5 | Urine: 52.6 μmol/L (N: 0.2-5.6) | Serum: 69.5 μmol/L (N: 0-15) | N/A | N/A  0.48 (N:<0.30) | Normal levels |
|  | 6 | Urine: 103.2 μmol/L (N: 0.2-5.6) | Serum: 128 μmol/L (N: 0-15) | N/A | N/A  0.56 (N:<0.30) | Normal levels |
|  | 7 | Urine: 91.9 μmol/L (N: 0.2-5.6) | Serum: 75.4 μmol/L (N: 0-15) | N/A | N/A  0.53 (N:<0.30) | Normal levels |
|  | 8 | Urine: 141.9 μmol/L (N: 0.2-5.6) | Serum: 111.8 μmol/L (N: 0-15) | N/A | N/A  0.34 (N:<0.30) | Normal levels |
|  | 9 | Urine: 140.9 μmol/L (N: 0.2-5.6) | Serum: 120.4 μmol/L (N: 0-15) | N/A | N/A  0.60 (N:<0.30) | Normal levels |
|  | 10 | Urine: 105.2 μmol/L (N: 0.2-5.6) | Serum: 109 μmol/L (N: 0-15) | N/A | N/A  0.35 (N:<0.30) | Normal levels |
|  | 11 | Urine: 236.5 μmol/L (N: 0.2-5.6) | Serum: 103.3 μmol/L (N: 0-15) | N/A | N/A  0.68 (N:<0.30) | Normal levels |
|  | 12 | Urine: 31 μmol/L (N: 0.2-5.6) | Serum: 77.2 μmol/L (N: 0-15) | N/A | N/A  0.52 (N:<0.30) | Normal levels |
|  | 13 | Urine: 97 μmol/L (N: 0.2-5.6) | Serum: 127.6 μmol/L (N: 0-15) | N/A | N/A  0.58 (N:<0.30) | Normal levels |
|  | 14 | Urine: 178 μmol/L (N: 0.2-5.6) | Serum: 115.2 μmol/L (N: 0-15) | N/A | N/A  0.72 (N:<0.30) | Normal levels |
|  | 15 | Urine: 116 μmol/L (N: 0.2-5.6) | Serum: 146.3 μmol/L (N: 0-15) | N/A | N/A  1.03 (N:<0.30) | Normal levels |
|  | 16 | Urine: 111.7 μmol/L (N: 0.2-5.6) | Serum: 114 μmol/L (N: 0-15) | N/A | N/A  0.96 (N:<0.30) | Normal levels |
|  | 17 | Urine: 130 μmol/L (N: 0.2-5.6) | Serum: 98 μmol/L (N: 0-15) | N/A | N/A  0.49 (N:<0.30) | Normal levels |
|  | 18 | Urine: 37.4 μmol/L (N: 0.2-5.6) | Serum: 61.4 μmol/L (N: 0-15) | N/A | N/A  0.44 (N:<0.30) | Normal levels |
|  | 19 | Urine: 34.9 μmol/L (N: 0.2-5.6) | Serum: 67.1 μmol/L (N: 0-15) | N/A | N/A  0.36 (N:<0.30) | Normal levels |
|  | 20 | Urine: 82.4 μmol/L (N: 0.2-5.6) | Serum: 62.3 μmol/L (N: 0-15) | N/A | N/A  0.51 (N:<0.30) | Normal levels |
|  | 21 | Urine: 141.6 μmol/L (N: 0.2-5.6) | Serum: 102.8 μmol/L (N: 0-15) | N/A | N/A  0.69 (N:<0.30) | Normal levels |
|  | 22 | Urine: 59.3 μmol/L (N: 0.2-5.6) | Serum: 193.4 μmol/L (N: 0-15) | N/A | N/A  0.56 (N:<0.30) | Normal levels |
|  | 23 | Urine: 229.1 μmol/L (N: 0.2-5.6) | Serum: 114.2 μmol/L (N: 0-15) | N/A | N/A  0.59 (N:<0.30) | Normal levels |
|  | 24 | Urine: 43.6 μmol/L (N: 0.2-5.6) | Serum: 57.5 μmol/L (N: 0-15) | N/A | N/A  0.62 (N:<0.30) | Normal levels |
|  | 25 | Urine: 65.7 μmol/L (N: 0.2-5.6) | Serum: 273.3 μmol/L (N: 0-15) | N/A | N/A  0.56 (N:<0.30) | Normal levels |
|  | 26 | Urine: 120.1 μmol/L (N: 0.2-5.6) | Serum: 103.3 μmol/L (N: 0-15) | N/A | N/A  0.89 (N:<0.30) | Normal levels |
| Wang et al. 2019 (3) | 1 | Urine: 232.18 μg/ml mg creatinine (N: 0.2-3.6) | Plasma: 101.60 μmol/L (N: 5-15) | N/A | N/A | N/A |
|  | 2 | Urine: 191.22 μg/ml mg creatinine (N: 0.2-3.6) | Plasma: 135.7 μmol/L (N: 5-15) | N/A | N/A | N/A |
|  | 3 | Urine: 70.53 μg/ml mg creatinine (N: 0.2-3.6) | Plasma: 57.2 μmol/L (N: 5-15) | N/A | N/A | N/A |
|  | 4 | Urine: 166.64 μg/ml mg creatinine (N: 0.2-3.6) | Plasma: 79.8 μmol/L (N: 5-15) | N/A | N/A | N/A |
|  | 5 | Urine: 321.12 μg/ml mg creatinine (N: 0.2-3.6) | Plasma: 99 μmol/L (N: 5-15) | N/A | N/A | N/A |
|  | 6 | Urine: 340.8 μg/ml mg creatinine (N: 0.2-3.6) | Plasma: 88 μmol/L (N: 5-15) | N/A | N/A | N/A |
|  | 7 | Urine: 172.4 μg/ml mg creatinine (N: 0.2-3.6) | Plasma: 97.7 μmol/L (N: 5-15) | N/A | N/A | N/A |
|  | 8 | Urine: 189.16 μg/ml mg creatinine (N: 0.2-3.6) | Plasma: 99.1 μmol/L (N: 5-15) | N/A | N/A | N/A |
|  | 9 | Urine: 253.68 μg/ml mg creatinine (N: 0.2-3.6) | Plasma: 115.3 μmol/L (N: 5-15) | N/A | N/A | N/A |
|  | 10 | Urine: 262.03 μg/ml mg creatinine (N: 0.2-3.6) | Plasma: 75.7 μmol/L (N: 5-15) | N/A | N/A | N/A |
|  | 11 | Urine: 168.05 μg/ml mg creatinine (N: 0.2-3.6) | Plasma: 121 μmol/L (N: 5-15) | N/A | N/A | N/A |
|  | 12 | Urine: 81.62 μg/ml mg creatinine (N: 0.2-3.6) | Plasma: 93.6 μmol/L (N: 5-15) | N/A | N/A | N/A |
|  | 13 | Urine: 58.42 μg/ml mg creatinine (N: 0.2-3.6) | Plasma: 102.6 μmol/L (N: 5-15) | N/A | N/A | N/A |
|  | 14 | Urine: 288.85 μg/ml mg creatinine (N: 0.2-3.6) | Plasma: 86 μmol/L (N: 5-15) | N/A | N/A | N/A |
|  | 15 | Urine: 116.04 μg/ml mg creatinine (N: 0.2-3.6) | Plasma: 114.1 μmol/L (N: 5-15) | N/A | N/A | N/A |
|  | 16 | Urine: 184.71 μg/ml mg creatinine (N: 0.2-3.6) | Plasma: 124.5 μmol/L (N: 5-15) | N/A | N/A | N/A |
| Wang et al. 2019 (4) | 1 | Urine: 101.5 μmol/L (N: 0.2-5.6) | Serum: 69.5 μmol/L (N: <15) | N/A | N/A | N/A |
| Wei et al. 2019 | 1 | Urine: 1514 (N: Increased fold over control)  Serum: 0.926 mg/dL (N: <0.047) | Serum: 217.4 μmol/L (N: 5-15) | N/A | 10.93 μmol/L (N: 0.5-4)  1.266 (N: 0.01–0.24) | Normal levels |
|  | 2 | Urine: 233.46 (N: Increased fold over control)  Serum: 0.453 mg/dL (N: <0.047) | Serum: 119.3 μmol/L (N: 5-15) | N/A | 7.06 μmol/L (N: 0.5-4)  0.82 (N: 0.01–0.24) | Normal levels |
|  | 3 | Urine: 1731.4 (N: Increased fold over control)  Serum: 0.947 mg/dL (N: <0.047) | Serum: 96.2 μmol/L (N: 5-15) | N/A | 9.32 μmol/L (N: 0.5-4)  0.91 (N: 0.01–0.24) | Normal levels |
|  | 4 | Urine: 344.7 (N: Increased fold over control)  Serum: 0.607 mg/dL (N: <0.047) | Serum: 153.3 μmol/L (N: 5-15) | N/A | 10.27 μmol/L (N: 0.5-4)  0.67 (N: 0.01–0.24) | Normal levels |
|  | 5 | Urine: 770.5 (N: Increased fold over control)  Serum: 0.821 mg/dL (N: <0.047) | Serum: 162 μmol/L (N: 5-15) | N/A | 12.31 μmol/L (N: 0.5-4)  0.71 (N: 0.01–0.24) | Normal levels |
|  | 6 | Urine: 44.1 (N: Increased fold over control)  Serum: 0.115 mg/dL (N: <0.047) | Serum: 75.4 μmol/L (N: 5-15) | N/A | 8.13 μmol/L (N: 0.5-4)  0.84 (N: 0.01–0.24) | Normal levels |
|  | 7 | Urine: 70.8 (N: Increased fold over control)  Serum: 0.147 mg/dL (N: <0.047) | Serum: 250.1 μmol/L (N: 5-15) | N/A | 9.05 μmol/L (N: 0.5-4)  0.49 (N: 0.01–0.24) | Normal levels |
|  | 8 | Urine: 395.91 (N: Increased fold over control)  Serum: 0.619 mg/dL (N: <0.047) | Serum: 221.8 μmol/L (N: 5-15) | N/A | 12.01 μmol/L (N: 0.5-4)  0.696 (N: 0.01–0.24) | Normal levels |
| Wei et al. 2020 | 1 | Urine: 112 times higher than normal | Serum: 223.6 μmol/L (N: 5-15) | N/A | 5.94 μmol/L (N: 0.5-4)  0.76 (N: 0.02-0.20) | Normal levels |
| Wen et al. 2020 | 1 | Urine: 49.7 mmol/molCr | Plasma: 155.8 μmol/L (N: 5-15) | 6.2 μmol/L (N: 8-45) | 7.3 μmol/L (N: 0.2-5)  N/A | Normal levels |
| Wu et al. 2017 | 1 | Urine: 253.68  mmol/mol/ creatinine (N: 0.2-3.6) | Plasma: 65 μmol/L (N: 0-20) | 8.86 μmol/L (N: 5.72-28.38) | N/A | N/A |
|  | 2 | Urine: 262.03  mmol/mol/ creatinine (N: 0.2-3.6) | Plasma: 115.3 μmol/L (N: 0-20) | 9.70 μmol/L (N: 5.72-28.38) | N/A | N/A |
| Wu et al. 2018 | 1 | Urine: elevated | Plasma: elevated | N/A | N/A | N/A |
|  | 2 | N/A | Plasma: elevated | N/A | N/A | N/A |
|  | 3 | N/A | Plasma: elevated | N/A | N/A | N/A |
| Zhao et al. 2021 | 1 | Serum: 101.5 μmol/L (N: 0.2-5.6)  Urine: 50.97 μmol/L (N: 0.2-3.6) | Serum: elevated | N/A | N/A | N/A |
|  | Total: 199 patients | Total measured: 178 patients  Elevated MMA levels: 177 patients  N/A: 21 patients | Total measured: 181 patients  Elevated Homocysteine levels: 181 patients  N/A: 18 patients | Total measured: 57 patients  Decreased Methionine levels: 28 patients  N/A: 142 patients | Total measured C3 levels: 41 patients  Total measured C3/C2 ratio: 63 patients  Elevated C3 levels: 35 patients  Elevated C3/C2 ratio: 58 patients  N/A: 131 patients | Total measured: 123 patients  Elevated or normal vitamin B12 levels: 123 patients  Elevated or normal acid folic levels: 122 patients  Decreased acid folic level: 1 patient  N/A: 76 patients |
| **Notes:**  **^1^** Additional data on Patient 1 and Patient 2 were taken from Motte et al. 2019  **^2^** Additional data on Patient 1 and data on Patient 2 were taken from Lemoine et al. 2018  **Abbreviations:** N/A: Not available, MMA: methylmalonic acid | | | | | | |

**Table 7. Other studies**

| **Author and year** | **# of cases** | **MRI** | | **Other studies** | | | | | | | |
| --- | --- | --- | --- | --- | --- | --- | --- | --- | --- | --- | --- |
|  |  | **Brain** | **Spinal** | **Echocardiogram** | **EEG** | **Renal ultrasonography** | **CT scan** | **EMG** | **Nerve pathology** | **Nerve Conduction Study** | **Other** |
| Ardissino et al. 2017 | 1 | N/A | N/A | Left ventricular hypertrophy and a slightly enlarged aortic bulb | N/A | Enlarged, hyperechogenic kidneys with reduced corticomedullary differentiation | N/A | N/A | N/A | N/A | N/A |
| Augoustides-Savvopoulou et al. 1999 | 1 | Dilation of the subarachnoid space frontoparietally | N/A | N/A | Frequent generalized bursts from slow waves (4-6 c/s) of high voltage and generalized bursts from slow-wave spike complexes (4 c/s) during photic stimulation | N/A | N/A | N/A | N/A | N/A | N/A |
| Backe et al. 2013 | 1 | Cerebellar edema | Extended high intensity lesion in the spinal cord at levels Th9–Th11 and C4–C6 | N/A | N/A | N/A | Cerebellar edema | N/A | Incipient sensomotoric polyneuropathy | N/A | Cerebrospinal fluid: traces of blood, leukocytes 33 per mm3, and total protein  0.71 g/L. CSF isoelectric: negative results. |
| Ben-Omran et al. 2007 | 1 | Diffuse cerebral atrophy with no focal lesions or active demyelination. | N/A | N/A | Normal | N/A | N/A | N/A | Sural nerve biopsy: significant epineural perivascular lymphocytic infiltrate and axonal neuropathy. | Nerve conduction velocities indicated a large fiber axonal neuropathy affecting motor and sensory nerves of upper and lower limbs. | Muscle biopsy: variability  in fiber diameter with many small type II fibers but no  abnormalities on histochemistry and electron microscopy. |
|  | 2 | 1º MRI: Volume loss within the corpus callosum and vermis and delay in peripheral myelin arborization and maturation  2º MRI: complete myelin arborization, but progressive  volume loss of supra and infratentorial structures.  There were subtle bilateral and symmetric  signal increase within the basal ganglia, involvement  was slightly more prominent within the globi pallidi | N/A | N/A | Bifrontal epileptiform activity. | N/A | N/A | N/A | N/A | N/A | N/A |
| Bodamer et al. 2001 | 1 | Normal | N/A | N/A | Normal | N/A | N/A | Scattered denervation in both legs and distal denervation in the left arm and mild chronic denervation of the tongue. | N/A | N/A |  |
| Boxer et al. 2005 | 1 | 1º MRI: Periventricular white matter lesions  2º MRI: confluent periventricular white matter hyperintensities in the frontal and parietal cortices  3º MRI (after starting treatment): decreased white matter hyperintensity in the patient’s centrum semiovale, despite a subjective loss of cortical volume | N/A | N/A | Normal | N/A | N/A | N/A | N/A | N/A | N/A |
|  | 2 | N/A | N/A | N/A | N/A | N/A | N/A | N/A | N/A | N/A | N/A |
| Brox-Torrecilla et al. 2021 | 1 | Brain MRI FLAIR-T2 Sequence. Diffuse supra and infratentorial cortico-subcortical retraction pattern (striking given the patient) | Dorsal spine MRI: Signal alteration that effects its entire length and is preferably located in white matter.  T2 cervical spine MRI: Extensive involvement of the white matter in relation to myelinolysis in the cervical and dorsal medulla. | N/A | N/A | N/A | N/A | N/A | N/A | N/A | N/A |
| Brunelli et al. 2002 | 1 | N/A | N/A | N/A | N/A | N/A | N/A | N/A | N/A | N/A | Light microscopic: patchy interstitial fibrosis, chronic inflammation, tubular  atrophy and thickened tubular basement membranes. Several tubules  contained protein casts. There was a variable appearance of  the glomeruli in the sample. |
| Chang et al. 2020 | 1 | Reversible cerebellar changes, delayed/  impaired myelination, periventricular hyperintensity | | N/A | N/A | N/A | N/A | Reduced tibial motor conduction velocity | Modest loss of myelinated fibers | N/A | MoCA: 15 |
|  | 2 | Periventricular hyperintensity | | N/A | N/A | N/A | N/A | Reduced tibial motor conduction velocity | Modest loss of myelinated fibers | N/A | MoCA: 26 |
|  | 3 | Normal | | N/A | N/A | N/A | N/A | Reduced tibial motor conduction velocity | Modest loss of myelinated fibers | N/A | MoCA: 28 |
|  | 4 | Hyperintensity was found in the terminal  areas of the white matter, which suggested delayed/impaired  myelination | | N/A | N/A | N/A | N/A | Reduced tibial motor conduction velocity | Modest loss of myelinated fibers | N/A | MoCA: 29 |
|  | 5 | Normal | | N/A | N/A | N/A | N/A | Peripheral nerve involvement | Modest loss of myelinated fibers | N/A | MoCA: 20 |
| Chu et al. 2020 | 1 | Cerebral cortical atrophy | N/A | N/A | N/A | N/A | N/A | N/A | N/A | Decreased motor nerve conduction velocities with reduced amplitude of CMAPs of common peroneal nerve and tibial nerve | N/A |
|  | 2 | N/A | N/A | N/A | N/A | N/A | N/A | N/A | N/A | Reduction of motor nerve conduction velocities with reduced  amplitude of CMAPs and prolonged distal motor latencies in  bilateral tibial nerve and prolonged distal motor latencies of  bilateral common peroneal nerve, absence of the sensory nerve  action potentials amplitude in the bilateral posterior tibial nerve, superficial peroneal nerve. | N/A |
|  | 3 | Cerebral cortical atrophy with lacunar infarcts (brain) | N/A | N/A | N/A | N/A | N/A | N/A | N/A | No obvious abnormality | N/A |
|  | 4 | Normal | N/A | N/A | N/A | N/A | N/A | N/A | N/A | The sympathetic skin response was prolonged in both upper limbs and lower limbs | N/A |
|  | 5 | Cerebral cortical atrophy | N/A | N/A | N/A | N/A | N/A | N/A | N/A | No obvious abnormality | N/A |
|  | 6 | Normal | N/A | N/A | N/A | N/A | N/A | N/A | N/A | Decreased motor nerve conduction velocities with reduced amplitude of CMAPs of common peroneal nerve and tibial nerve. No sympathetic skin response in the right lower limb and prolonged latency of the left lower limb were recorded | N/A |
|  | 7 | Cerebral cortical atrophy | N/A | N/A | N/A | N/A | N/A | N/A | N/A | Decreased amplitude of sensory nerve action potentials of right sural nerve and decreased amplitude of CMAPs of left common peroneal nerve | N/A |
|  | 8 | Normal | N/A | N/A | N/A | N/A | N/A | N/A | N/A | Reduction of sensory nerve conduction velocities of the right sural nerve, the prolonged distal motor latencies and decreased amplitude of the CMAPs and reduction of motor nerve conduction velocities of the right tibial nerve and the left common peroneal nerve | N/A |
| Collison et al. 2015 | 1 | N/A | N/A | N/A | N/A | N/A | N/A | N/A | N/A | N/A | Goldmann visual field testing: pericentral ring scotoma in each eye  Funduscopy: bilateral bull’s eye-appearing macular lesions with no visible fundus flecks, vessel attenuation, optic nerve pallor or peripheral pigmentary changes  A scotopic and photopic full-field electroretinogram (ERG) of the right eye: normal a- and b-wave amplitudes and implicit times |
| Cornec-Legall et al. 2014 | 1 | Normal | N/A | Normal | N/A | Kidney ultrasonography  and Doppler were normal. | Normal | N/A | N/A | N/A | Kidney biopsy showed  typical glomerular and arteriolar TMA.  Second kidney biopsy  showed a severe degree of glomerular and arteriolar TMA and 1 scleroti glomerulus out of 16.  Ophthalmologic evaluation, including ocular funduscopy,  excluded pigmentary retinopathy. |
| Cui et al. 2019 | 1 | N/A | Neck MRI: symmetrical long T2 signals in the posterior portion of the spinal cord in the 2 to 6 vertebral bodies | N/A | N/A | N/A | N/A | N/A | N/A | The bilateral lower limb sensory evoked potential showed  abnormalities (central segment) | Cerebrospinal fluid routine examination was normal. |
|  | 2 | N/A | N/A | N/A | N/A | N/A | N/A | N/A | N/A | N/A | N/A |
|  | 3 | N/A | N/A | N/A | N/A | N/A | N/A | N/A | N/A | N/A | N/A |
|  | 4 | Right choroid cyst, the cavity of septum pellucidum, and slightly wider sulci | Abnormally long T2 weighted image (T2W1) signal in the posterior columns from T8 to T11 | N/A | Normal | Normal | N/A | N/A | N/A | N/A | Chest radiography: normal.  Cerebrospinal fluid  routine examination was normal, except that the lactic acid level  was 3.43mmol/L. |
|  | 5 | N/A | N/A | N/A | N/A | N/A | N/A | N/A | N/A | N/A | N/A |
| Davin et al. 2009 | 1 | N/A | N/A | N/A | N/A | N/A | N/A | N/A | N/A | N/A | N/A |
| Gerth et al. 2008 | 1 | N/A | N/A | N/A | N/A | N/A | N/A | N/A | N/A | N/A | N/A |
|  | 2 | N/A | N/A | N/A | N/A | N/A | N/A | N/A | N/A | N/A | N/A |
|  | 3 | N/A | N/A | N/A | N/A | N/A | N/A | N/A | N/A | N/A | N/A |
|  | 4 | N/A | N/A | N/A | N/A | N/A | N/A | N/A | N/A | N/A | N/A |
| Gilson et al. 2018 | 1 | N/A | N/A | N/A | N/A | N/A | N/A | N/A | N/A | N/A | N/A |
| Gold et al. 1996 | 1 | Normal | | N/A | N/A | N/A | N/A | Chronic denervation | Predominantly axonal neuropathy | A reduction in amplitude and a low normal conduction velocity | N/A |
|  | 2 | N/A | N/A | N/A | N/A | N/A | N/A | N/A | N/A | Normal motor conduction velocity of the peroneal nerve (58 m/s; normal > 43 m/s).  Tibial nerve conduction velocity was at the lower limit of normal | N/A |
| Goodman et al. 1970 | 1 | N/A | N/A | N/A | N/A | N/A | N/A | N/A | N/A | N/A | N/A |
|  | 2 | N/A | N/A | N/A | N/A | N/A | N/A | N/A | N/A | N/A | N/A |
| Grandone et al. 2019 | 1 | N/A | N/A | N/A | N/A | N/A | N/A | N/A | N/A | N/A | Neurological examination: normal  Blood smear: normal  Bone marrow aspiration: normal  A renal biopsy revealed thrombotic microangiopathy (TMA) with predominant lesions in the glomerulus and  minimal lesions in the arterioles. |
| Grangé et al. 2015 | 1 | Normal | N/A | Raised systolic pulmonary artery pressure with left ventricular hypertrophy without systolic dysfunction | N/A | N/A | Normal kidney morphology and pulmonary oedema. | N/A | N/A | N/A | Renal histology: major stenosing fibroproliferative myxoid lesions in the interlobular arteries, consistent with renal TMA.  The glomeruli had an ischaemic appearance with thickened, ribbon-like glomerular basement membranes; there was no proliferation, intracapillary thrombosis, or glomerular endothelial lesion. |
|  | 2 | N/A | N/A | N/A | N/A | N/A | N/A | N/A | N/A | N/A | N/A |
| Guigonis et al. 2005 | 1 | Normal | | N/A | N/A | N/A | N/A | N/A | N/A | N/A | Renal biopsy: typical features of glomerular and arterial thrombotic microangiopathy |
|  | 2 | N/A | N/A | N/A | N/A | N/A | N/A | N/A | N/A | N/A | Renal biopsy: typical features of glomerular and arterial thrombotic microangiopathy |
| Gündüz et al. 2014 | 1 | N/A | N/A | A right ventricular and atrial  dilatation. The right ventricular diastolic dimension was  32 mm (Z score +3.9). They noticed a first-degree tricuspid  valve regurgitation (velocity 4.7 m/s) and a small patent  foramen ovale, which was shunting from the left atrium to  the right atrium. The systolic and mean pulmonary  arterial pressures were estimated as 94 and 55 mmHg, respectively, | N/A | N/A | CT thorax: Normal | N/A | N/A | N/A | ECG displayed a right axis deviation, right ventricular hypertrophy, and increased P waves.  Cardiac catheterization:  the systolic and mean pulmonary arterial pressures were  55 and 45 mmHg, respectively. The ratio of pulmonary and  systemic arterial resistances (resistance index) was 0.12. |
| Gurkas et al. 2015 | 1 | 1º Before treatment: Cortical atrophy and symmetrical signa increase within the basal ganglia and bilateral patchy focal hyperintensities in the white matter at the level of centrum semiovale corresponding to corticospinal tract regions | N/A | N/A | Diffuse low-amplitude slow waves | N/A | N/A | N/A | N/A | N/A | N/A |
| Heil et al. 2007 | 1 | Normal | N/A | Normal | Normal | N/A | N/A | N/A | N/A | N/A | ECG y ophtalmological evaluation were normal |
|  | 2 | Normal | N/A | Normal | Normal | N/A | N/A | N/A | N/A | N/A | ECG y ophtalmological evaluation were norma |
| Higashimoto et al. | 1 | Diffuse periventricular white matter changes and basal ganglia involvement | 1º Extensive dorsal white matter involvement extending from C4 to C5  2º Extensive dorsal white matter lesions extending  from C2-T10 of the spinal cord | N/A | N/A | N/A | N/A | N/A | N/A | N/A | N/A |
|  | 2 | Normal | | N/A | N/A | N/A | N/A | N/A | N/A | N/A | N/A |
|  | 3 | Normal | | N/A | N/A | N/A | N/A | N/A | N/A | N/A | N/A |
| Huemer et al. 2014 | 1 | N/A | N/A | Marked concentric left ventricular hypertrophy with a moderately dilated left atrium | N/A | Normal | N/A | N/A | N/A | N/A | ECG: inverted T waves.  Ophthalmoscopy: grade III retinopathy with hemorrhages compatible with chronic hypertension. |
|  | 2 | N/A | MRI scan of the myelon supported the idea of subacute thoracal myelopathy with hypoaesthesia, disturbance of position sense, pathological reflex pattern and sudden gait difficulties | N/A | N/A | N/A | N/A | N/A | N/A | N/A | N/A |
|  | 3 | Large areas of signal abnormality without enhancement predominantly involving the white matter of both hemispheres, and extending to the midbrain structures | Progression of the signal abnormalities with additional involvement of the spinal cord. | N/A | N/A | N/A | N/A | N/A | N/A | N/A | CSF: minimal increase of lactate, normal cell counts, protein and glucose. |
| Iodice et al. 2013 | 1 | N/A | N/A | Normal pulmonary and systemic venous return, moderate tricuspid insufficiency with a right ventricular systolic pressure of 75 mmHg (systemic blood pressure 103/70mmHg), and moderate left ventricular dysfunction with nonsignificant mitral insufficiency.Z score was 23.4 with an ejection fraction of 38.5% | N/A | N/A | Increased size of the pulmonary artery and signs of pulmonary hypertension | N/A | N/A | N/A | Chest radiograph: marked interstitial thickening and a normal cardiac size  The catherization: confirmed pulmonary hypertension  responsive to oxygen and nitric  oxide. The following values were reported  in the pulmonary artery: baseline 91/41/  66 mmHg (normal values = 15–25/8–15/  20), after 100% oxygen 64/24/44 mmHg,  after nitric oxide 51/23/36 mmHg. Baseline  pulmonary arteriole resistances  were 15.4 unit woods/m2 (normal values  1–3 U.W./m2) after 100% oxygen and  nitric oxide; values were lower but  remained high respectively at 6.2 U.W./m2 and 6.4 U.W./m2. |
|  | 2 | N/A | N/A | N/A | N/A | N/A | N/A | N/A | N/A | N/A | N/A |
| Jiménez-Varo et al. 2015 | 1 | N/A | N/A | N/A | N/A | Bilateral renal echodoppler with enhancer: presence  of a bilateral renal edema. | Presence of a 7 mm hemorrhagic cyst in the cortical thickness of the left kidney | N/A | N/A | N/A | N/A |
| Kılıç et al. 2013 | 1 | Bilateral signal intensity changes in the basal ganglia and cerebral white matter and diffuse atrophy of the corpus callosum and cerebrum | N/A | N/A | Normal | N/A | N/A | N/A | N/A | N/A | N/A |
| Koenig et al. 2015 | 1 | N/A | N/A | Left ventricular hypertrophy secondary to chronic arterial hypertension but no signs of pulmonary hypertension. | N/A | Enlarged size and echogenicity of both kidneys with regular blood flow velocity and resistance indices. Neither ascites nor pleural effusions were detectable | N/A | N/A | N/A | N/A | Renal biopsy: TMA with ischemic glomerular collapse and focal and segmental splitting of glomerular basement membranes. |
| Kömhoff et al. 2013 | 1 | N/A | N/A | PAH, with no identifiable cause. | N/A | N/A | N/A | N/A | N/A | N/A | Renal biopsy: TMA. |
|  | 2 | N/A | N/A | Biventricular hypertrophy, dilated right ventricle with impaired function, increased pressure in the right ventricle, pulmonary arteries and pericardial effusion. | N/A | N/A | N/A | N/A | N/A | N/A | N/A |
|  | 3 | N/A | N/A | N/A | N/A | N/A | N/A | N/A | N/A | N/A | Renal biopsy: TMA  Right heart  catheterization  PVR (RU: <2.5) 4.9  mPAP (<20 mmHg) 30 |
|  | 4 | N/A | N/A | No signs of PAH | N/A | N/A | N/A | N/A | N/A | N/A | Renal biopsy: TMA  Right heart  catheterization  PVR (RU: <2.5) 5.2  mPAP (<20 mmHg) 26 |
|  | 5 | N/A | N/A | N/A | N/A | N/A | N/A | N/A | N/A | N/A | Renal biopsy: TMA  Right heart  catheterization  PVR (RU: <2.5) 7.6  mPAP (<20 mmHg) 68 |
| Lemoine et al. 2018 | 1,2, 3, 4, 5, 6, 7 | N/A | N/A | N/A | N/A | N/A | N/A | N/A | N/A | N/A | Renal biopsy: Light microscopic evaluation  revealed glomerular and arteriolar TMA.  Most of the glomeruli presented typical lesions of  TM, with thickening of the capillary wall. |
| Li et al. 2015 | 1 | N/A | N/A | N/A | N/A | N/A | N/A | N/A | N/A | N/A | N/A |
|  | 2 | N/A | N/A | N/A | N/A | N/A | N/A | N/A | N/A | N/A | Renal biopsy: homocysteine  crystals in kidney tubules but few lesions of thrombotic microangiopathy. |
|  | 3 | N/A | N/A | N/A | N/A | N/A | N/A | N/A | N/A | N/A | N/A |
| Lin et al. 2009 | 1 | N/A | N/A | N/A | N/A | N/A | N/A | N/A | N/A | N/A | N/A |
| Liu et al. 2015 (1) | 1 | Mild brain atrophy | Normal | N/A | N/A | N/A | N/A | Decreased conduction velocity of the peripheral nerve in both legs | N/A | N/A | N/A |
|  | 2 | Mild brain atrophy | Normal | N/A | N/A | N/A | N/A | Decreased conduction velocity of the peripheral nerve in both legs | N/A | N/A | N/A |
|  | 3 | Mild brain atrophy | Normal | N/A | N/A | N/A | N/A | Normal | N/A | N/A | N/A |
|  | 4 | Mild brain atrophy  Hyperintensity on the T2-weighted image and DWI and hypointensity on the ADC map of the bilateral cerebellar cortex | Normal | N/A | N/A | N/A | N/A | Decreased conduction velocity of the peripheral nerve in both legs | N/A | N/A | N/A |
|  | 5 | Mild brain atrophy | Normal | N/A | N/A | N/A | N/A | normal | N/A | N/A | N/A |
| Liu et al. 2015 (2) | 1 | Mild cerebral atrophy | N/A | N/A | Normal | N/A | N/A | N/A | N/A | N/A | N/A |
| Liu et al. 2017 | 1 | Dilated lateral ventricles with mild hydrocephalus | N/A | Moderate PAH (TRPG 68 mmHg) with moderate dilation of the right atrium and ventricle, mild tricuspid and pulmonary valve regurgitation, and an ejection fraction (EF) of 74%. | N/A | Echo enhancement of the renal parenchyma on both sides | Diffuse ground-glass opacification, interlobular septal thickening | N/A | N/A | N/A | N/A |
|  | 2 | Normal | N/A | Severe PAH (TRPG 81 mmHg) with moderate dilation of the right atrium and ventricle, EF 68%, moderate tricuspid regurgitation and mild pulmonary regurgitation. | N/A | Mild enlargement of both kidneys | Diffuse poorly defined centrilobular nodules,  interlobular septal thickening | N/A | N/A | N/A | N/A |
|  | 3 | Evident demyelinating lesions | N/A | Normal | N/A | Echo enhancement and diffuse injury to  the renal parenchyma on both sides | Diffuse poorly defined ground-glass  centrilobular nodules | N/A | N/A | N/A | N/A |
|  | 4 | Evident demyelinating lesions | N/A | Severe PAH | N/A | Normal | Mild diffuse ground-glass opacification | N/A | N/A | N/A | N/A |
| Liu et al. 2020 | 1 | N/A | N/A | Mild PAH | N/A | N/A | Lung computed tomography showing the presence in both lungs of diffuse poorly defined ground‐glass centrilobular nodules | N/A | N/A | N/A | N/A |
|  | 2 | N/A | N/A | Severe PAH | N/A | N/A | Lung computed tomography showing the presence in both lungs of diffuse poorly defined ground‐glass centrilobular nodules | N/A | N/A | N/A | N/A |
|  | 3 | N/A | N/A | Moderate PAH | N/A | N/A | Lung computed tomography showing the presence in both lungs of diffuse ground‐glass opacification | N/A | N/A | N/A | N/A |
|  | 4 | N/A | N/A | Severe PAH | N/A | N/A | Lung computed tomography showing the presence in both lungs of diffuse poorly defined ground‐glass centrilobular nodules | N/A | N/A | N/A | Thoracoscopic lung biopsy findings confirmed microangiopathy and negative homocysteine  in immunohistochemistry. |
| Losito et al. 2012 | 1 | N/A | N/A | 1º Normal  2º Enlargement, hypertrophy and hypokinetics of the right ventricle and a pulmonary artery with severe incontinency of the tricuspid and pulmonary valves | N/A | Extremely enlarged kidneys | N/A | N/A | N/A | N/A | Chest X-rays and ECG were normal  Renal biopsy: All of the 9 glomeruli examined by light microscopy showed a mild increase in mesangial cell numbers and matrix and diffuse thickening of the glomerular capillary walls with double contours and within the splitting empty spaces with honeycomb aspects.  Heart catheterization: cardiac output of 3.83 l/min; pulmonary artery pressure of 98 mmHg (systolic 15 – 30), 54 mmHg (diastolic 8 – 15) with a mean value of 68 mmHg (9 – 18); pulmonary capillary wedge pressure 15 mmHg (6 – 13); pulmonary vascular resistance 1,054 dyne × s × cm-5 (20 – 130). |
| Mitchell et al. 1986 | 1 | N/A | N/A | N/A | Slow wave  activity and paroxysmal wave forms. | N/A | TC craneal: the lateral ventricles were at the upper limits of normal size. | N/A | N/A | N/A | N/A |
| Navarro et al. 2018 | 1 | N/A | N/A | No signs of pulmonary hypertension | N/A | N/A | N/A | N/A | N/A | N/A | Renal biopsy: severe TMA glomerular and vascular lesions. |
| Nogueira et al. 2017 | 1 | N/A | N/A | N/A | N/A | N/A | N/A | N/A | N/A | N/A | N/A |
|  | 2 | N/A | N/A | N/A | N/A | N/A | N/A | N/A | N/A | N/A | N/A |
|  | 3 | N/A | N/A | N/A | N/A | N/A | N/A | N/A | N/A | N/A | N/A |
|  | 4 | N/A | N/A | N/A | N/A | N/A | N/A | N/A | N/A | N/A | N/A |
|  | 5 | N/A | N/A | N/A | N/A | N/A | N/A | N/A | N/A | N/A | N/A |
| Petropoulos et al. 2018 | 1 | Normal | N/A | 1º ETT: Normal left ventricular size and function with a left ventricle ejection fraction (LVEF) >55%, normal right ventricle size and systolic function, mild-to-moderate mitral regurgitation and no evidence of pulmonary hypertension based on the right ventricular systolic pressure.  2º ETT: Significant changes with mildly decreased left ventricular (LV) systolic function (LVEF 50%) and findings suggestive of right ventricular pressure and volume overload, specifically a RVSP of 65mmHg, moderate pulmonary regurgitation, severe tricuspid regurgitation, severely enlarged and thickened right ventricle and a 14-mm pericardial effusion. | N/A | Increased echogenicity of the renal cortical tissue of the right and left kidneys, which measured 8.7 and 9.9 cm, respectively. | TC thorax: enlarged pulmonary artery at 42mm, a moderately sized pericardial effusion, extensive centrilobular ground glass nodules throughout both lungs and some interlobular septal thickening in the right lower lobe. | N/A | N/A | N/A | Renal biopsy: severe thrombotic microangiopathy with 3 out of 24 glomeruli globally sclerosed with moderate interstitial fibrosis and tubular atrophy.  Right heart catheterization: severe pulmonary hypertension. |
| Philipponnet et al. 2020 | 1 | N/A | N/A | Preserved left ventricular function | N/A | Excluded an obstructive cause of acute kidney injury | N/A | N/A | N/A | N/A | Kidney biopsy: mesangial sclerosis, noticeable thickening of the capillary wall with a duplication aspect of the glomerular basement membrane (GBM) and the presence of intraglomerular and intravascular thrombi. No tubular or interstitial damage was observed. |
| Pollini et al. 2020 | 1 | Multiple subcortical and periventricular white matter lesions suggesting the diagnosis of MS | N/A | N/A | N/A | N/A | N/A | N/A | N/A | N/A | N/A |
| Powers et al. 2001 | 1 | A confluent periventricular white matter process about both trigone regions and the posterior body of the lateral ventricles. | N/A | N/A | N/A | N/A | N/A | N/A | N/A | Nerve conduction in the posterior tibial and sural nerves was mildly slowed. | CSF: protein level increased  Autopsy: cerebral perivascular demyelination |
|  | 2 | Proton density and T2-weightedMRI demonstrated periventricular nonenhancing increased signal abnormalities that were confluent in the occipital lobes and patchy in frontal white matter.  T1-weighted images were normal. | N/A | N/A | 3.5-Hz background. | N/A | Craneal TC: normal | N/A | The sural nerve: a loss of large and, especially, small myelinated axons with ongoing axonal degeneration. | N/A | CSF: protein level increased.  Brain biopsy: some perivascular demyelinative foci and a few perivascular lymphocytes without specific diagnostic features.  Autopsy: perivascular demyelination of white matter |
| Profitlich et al. 2009 | 1 | N/A | N/A | Age at evaluation 24.5 years: Normal structure. Left ventricular function (Shortening fraction %) 33% | N/A | N/A | N/A | N/A | N/A | N/A | N/A |
| Rahmandar et al. 2014 | 1 | Increased T2 signal in the cerebellum. | N/A | N/A | A left temporal epileptic focus and diffuse encephalopathy. | N/A | Craneal TC: normal | N/A | N/A | N/A | N/A |
| Roze et al. 2003 | 1 | Normal | Normal | N/A | N/A | N/A | N/A | N/A | N/A | N/A | N/A |
|  | 2 | Mild cortical atrophy and bilateral hyperintensity in the periventricular white matter on T2-weighted and flair images. | N/A | N/A | Diffuse slow waves. | N/A | N/A | N/A | Sural nerve biopsy: specimen displayed demyelination and wallerian degeneration. | Intermediate conduction velocity with reduction of amplitude and denervation. | N/A |
| Shinnar et al. 1984 | 1 | N/A | N/A | N/A | Markedly abnormal, showing pronounced diffuse slowing with an anterior maximum. | N/A | Moderate generalized cortical atrophy with mild ventricular enlargement | N/A | N/A | N/A | N/A |
|  | 2 | N/A | N/A | N/A | Small amount of bilateral slowing. | N/A | N/A | N/A | N/A | N/A | N/A |
| Thauvin-Robinet et al. 2007 | 1 | Leucoencephalopathy with corpus callosum agenesis and bilateral ventricular dilatation. | N/A | N/A | N/A | N/A | Craneal TC: Cortical  atrophy and leucoencephalopathy. | N/A | N/A | N/A | N/A |
|  | 2 | Mild periventricular leucoencephalopathy | Diffuse, abnormal, high intensity signal on T2 weighted sequences involving the posterior columns and pyramidal tracts at the cervical and dorsal levels. | N/A | N/A | N/A | N/A | N/A | N/A | N/A | Electromyography and fundoscopy were normal. |
|  | 3 | Normal | N/A | N/A | N/A | N/A | N/A | N/A | N/A | N/A | N/A |
| Tsai et al. 2007 | 1 | Areas of hyperintensity in T2-weighted consistent with a demyelinating process | Spinal infarct | N/A | N/A | N/A | N/A | N/A | N/A | N/A | N/A |
| Van Hove et al. 2002 | 1 | N/A | N/A | Normal | N/A | Normal | Cortical hypodense zones on a computer tomography (CT) scan. | N/A | N/A | N/A | Renal biopsy: elsewhere as a membranoproliferative glomerulonephritis with disruption of the basement membrane. |
|  | 2 | N/A | N/A | Normal | N/A | Increased size and echogenicity | N/A | N/A | N/A | N/A | Renal biopsy: membranoproliferative glomerulonephritis. |
| Wang et al. 2012 | 1 | Bilateral hyperintensity in the deep white matter in the T2 image and fluid attenuated inversion recovery /FLAIR) image.  Brain MRS:  decreased N-acetylaspartate (NAA), and increased choline (Cho) and lactate peaks. | N/A | N/A | N/A | N/A | N/A | Peripheral nerves in both legs were involved, by revealing both a decreased conduction velocity in motor nerves (bilateral sural nerve: 38 m/s (right) and 34 m/s (left)) and in sensory nerves (left tibial: 34 m/s), and a reduction in amplitude (left tibial nerve 1.7 μV). | N/A | N/A | N/A |
|  | 2 | Cerebral atrophy in the T1 image, and bilateral cerebellar abnormalities were detected using FLAIR and diffusion-weighted imaging (DWI) | N/A | N/A | A moderate abnormality results (5–7 C/S chronic wave, sporadic positive sharp wave, moderate arrhythmia at all leads). | N/A | N/A | Reduced electrical conduction velocity in the peripheral nerve. | N/A | N/A | N/A |
|  | 3 | Lacunar infarction in the deep white matter in the T2 image and the FLAIR image.  MRS testing performed on the brain tissue showed normal NAA and Cho peaks, and a lactate peak was not evident. | N/A | N/A | N/A | N/A | N/A | There was peripheral nerve involvement, indicated by reduced conduction velocity (sensory nerve: right tibial nerve 34 m/s; left sural nerve 26 m/s), reduction of conduction wave amplitude (motor nerve: right peroneal nerve 1.74 mV; sensory nerve: right tibial nerve 1.02 μV), and extended latency in motor nerves (right peroneal nerve 7.2 ms, left peroneal nerve: 5.5 ms). | N/A | N/A | N/A |
| Wang et al. 2018 | 1 | N/A | Thoracic cord atrophy | N/A | N/A | N/A | N/A | N/A | N/A | N/A | Spine X-ray: Scoliosis |
|  | 2 | N/A | N/A | N/A | N/A | N/A | N/A | N/A | N/A | N/A | N/A |
|  | 3 | Cerebrum hyperintensity/ atrophy | Thoracic cord atrophy | N/A | N/A | N/A | N/A | N/A | N/A | N/A | Spine X-ray: Scoliosis |
|  | 4 | N/A | N/A | N/A | N/A | N/A | N/A | N/A | N/A | N/A | N/A |
|  | 5 | Cerebrum atrophy | N/A | N/A | N/A | N/A | N/A | N/A | N/A | N/A | Spine X-ray: Scoliosis |
|  | 6 | Cerebrum atrophy | Thoracic cord atrophy | N/A | N/A | N/A | N/A | N/A | N/A | N/A | Spine X-ray: Scoliosis |
|  | 7 | Cerebrum atrophy | Thoracic cord atrophy | N/A | N/A | N/A | N/A | N/A | N/A | N/A | Spine X-ray: Scoliosis, bow feet |
|  | 8 | N/A | N/A | N/A | N/A | N/A | N/A | N/A | N/A | N/A | N/A |
| Wang et al. 2019 (1) | 1 | N/A | N/A | N/A | N/A | N/A | N/A | N/A | N/A | N/A | N/A |
|  | 2 | N/A | N/A | N/A | N/A | N/A | N/A | N/A | N/A | N/A | N/A |
|  | 3 | N/A | N/A | N/A | N/A | N/A | N/A | N/A | N/A | N/A | N/A |
|  | 4 | N/A | N/A | N/A | N/A | N/A | N/A | N/A | N/A | N/A | N/A |
|  | 5 | N/A | N/A | N/A | N/A | N/A | N/A | N/A | N/A | N/A | N/A |
|  | 6 | N/A | N/A | N/A | N/A | N/A | N/A | N/A | N/A | N/A | N/A |
|  | 7 | N/A | N/A | N/A | N/A | N/A | N/A | N/A | N/A | N/A | N/A |
|  | 8 | N/A | N/A | N/A | N/A | N/A | N/A | N/A | N/A | N/A | N/A |
|  | 9 | N/A | N/A | N/A | N/A | N/A | N/A | N/A | N/A | N/A | N/A |
|  | 10 | N/A | N/A | N/A | N/A | N/A | N/A | N/A | N/A | N/A | N/A |
|  | 11 | N/A | N/A | N/A | N/A | N/A | N/A | N/A | N/A | N/A | N/A |
|  | 12 | N/A | N/A | N/A | N/A | N/A | N/A | N/A | N/A | N/A | N/A |
|  | 13 | N/A | N/A | N/A | N/A | N/A | N/A | N/A | N/A | N/A | N/A |
|  | 14 | N/A | N/A | N/A | N/A | N/A | N/A | N/A | N/A | N/A | N/A |
| Wang et al. 2019 (2) | 1 | Cerebellum atrophy | Cervical and thoracic cord atrophy | N/A | N/A | N/A | N/A | Mild peripheral nerve damage | N/A | N/A | Spine X-ray: Scoliosis |
|  | 2 | Normal | Thoracic cord atrophy | N/A | N/A | N/A | N/A | Mild peripheral nerve damage | N/A | N/A | Spine X-ray: Scoliosis |
|  | 3 | Normal | Thoracic cord atrophy | N/A | N/A | N/A | N/A | N/A | N/A | N/A | Spine X-ray: Scoliosis |
|  | 4 | Normal | Longitudinally extensive transverse myelitis (cervical cord) | N/A | N/A | N/A | N/A | Normal | N/A | N/A | Spine X-ray: Scoliosis |
|  | 5 | Hyperintensity of basal ganglia and cerebellum | Thoracic cord atrophy | N/A | N/A | N/A | N/A | Peripheral nerve damage | N/A | N/A | Spine X-ray: Scoliosis |
|  | 6 | Normal | | N/A | N/A | N/A | N/A | N/A | N/A | N/A | N/A |
|  | 7 | Normal | Longitudinally extensive transverse myelitis (cervical cord) | N/A | N/A | N/A | N/A | Peripheral nerve damage | N/A | N/A | Spine X-ray: Scoliosis |
|  | 8 | Normal | Thoracic cord atrophy | N/A | N/A | N/A | N/A | Normal | N/A | N/A | Spine X-ray: Scoliosis |
|  | 9 | Cerebral cortical atrophy | N/A | N/A | N/A | N/A | N/A | Mild peripheral nerve damage | N/A | N/A | Spine X-ray: Scoliosis |
|  | 10 | Cerebral cortical atrophy; white matter demyelination | N/A | N/A | N/A | N/A | N/A | N/A | N/A | N/A | Spine X-ray: Scoliosis |
|  | 11 | Hydrocephalus | N/A | N/A | N/A | N/A | N/A | Peripheral nerve damage | N/A | N/A | Spine X-ray: Scoliosis |
|  | 12 | Cerebellum atrophy | N/A | N/A | N/A | N/A | N/A | N/A | N/A | N/A | N/A |
|  | 13 | Cerebral cortical atrophy | N/A | N/A | N/A | N/A | N/A | N/A | N/A | N/A | Spine X-ray: Scoliosis |
|  | 14 | Cerebral cortical atrophy | Cervical and thoracic cord atrophy | N/A | N/A | N/A | N/A | Peripheral nerve damage | N/A | N/A | Spine X-ray: Scoliosis |
|  | 15 | Reversible posterior leukoencephalopathy syndrome | N/A | N/A | N/A | N/A | N/A | Peripheral nerve damage | N/A | N/A | N/A |
|  | 16 | Cerebral cortical atrophy | Thoracic cord atrophy | N/A | N/A | N/A | N/A | N/A | N/A | N/A | Spine X-ray: Scoliosis |
|  | 17 | Normal | Thoracic cord atrophy | N/A | N/A | N/A | N/A | Normal | N/A | N/A | Spine X-ray: Scoliosis |
|  | 18 | Normal | Thoracic cord atrophy | N/A | N/A | N/A | N/A | Mild peripheral nerve damage | N/A | N/A | N/A |
|  | 19 | Normal | Longitudinally extensive transverse myelitis | N/A | N/A | N/A | N/A | Mild peripheral nerve damage | N/A | N/A | Spine X-ray: Scoliosis |
|  | 20 | Normal | N/A | N/A | N/A | N/A | N/A | N/A | N/A | N/A | Spine X-ray: Scoliosis |
|  | 21 | N/A | Normal | N/A | N/A | N/A | N/A | Peripheral nerve damage | N/A | N/A | Spine X-ray: Scoliosis |
|  | 22 | Cerebral cortical atrophy | Thoracic cord atrophy | N/A | N/A | N/A | N/A | Peripheral nerve damage | N/A | N/A | Spine X-ray: Scoliosis |
|  | 23 | Cerebral cortical atrophy | Thoracic cord atrophy | N/A | N/A | N/A | N/A | Peripheral nerve damage | N/A | N/A | N/A |
|  | 24 | Cerebral white matter demyelination | Longitudinally extensive transverse myelitis | N/A | N/A | N/A | N/A | Mild peripheral nerve damage | N/A | N/A | N/A |
|  | 25 | Normal | N/A | N/A | N/A | N/A | N/A | Mild peripheral nerve damage | N/A | N/A | N/A |
|  | 26 | Normal | N/A | N/A | N/A | N/A | N/A | N/A | N/A | N/A | N/A |
| Wang et al. 2019 (3) | 1 | Bilateral white matter lesions in the centrum ovale and corona radiata | Thoracic lesions | N/A | N/A | N/A | N/A | Sensorimotor polyneuropathy of lower limbs (involved both axon and myelin) | N/A | N/A | N/A |
|  | 2 | Bilateral white matter lesions in the bilateral periventricular white matter | N/A | N/A | Epileptic discharges | N/A | N/A | N/A | N/A | N/A | N/A |
|  | 3 | Cerebral atrophy and bilateral cerebellar cortex lesions | N/A | N/A | N/A | N/A | N/A | Sensorimotor polyneuropathy of lower limbs (involved both axon and myelin) | N/A | N/A | N/A |
|  | 4 | Bilateral white matter lesions | N/A | N/A | N/A | N/A | N/A | Sensorimotor polyneuropathy of lower limbs (involved myelin) | N/A | N/A | N/A |
|  | 5 | Cerebral atrophy | Normal | N/A | Epileptic discharges | N/A | N/A | N/A | N/A | N/A | N/A |
|  | 6 | Mild cerebral atrophy | N/A | N/A | N/A | N/A | N/A | N/A | N/A | N/A | N/A |
|  | 7 | Cerebral atrophy | Normal | N/A | N/A | N/A | N/A | N/A | N/A | N/A | Visual acuity: normal  Funduscopy examination: optic nerve atrophy |
|  | 8 | Cerebral atrophy and white matter lesions in unilateral posterior ventricular area | N/A | N/A | N/A | N/A | N/A | N/A | N/A | N/A | N/A |
|  | 9 | Normal | N/A | N/A | N/A | N/A | N/A | N/A | N/A | N/A | Visual acuity: normal  Funduscopy examination: mild optic nerve atrophy, pigmentary retinal dystrophy |
|  | 10 | Mild cerebral atrophy | N/A | N/A | N/A | N/A | N/A | N/A | N/A | N/A | N/A |
|  | 11 | Cerebral atrophy | Normal | N/A | N/A | N/A | N/A | N/A | N/A | N/A | N/A |
|  | 12 | Normal | | N/A |  | N/A | N/A | N/A | N/A | N/A | N/A |
|  | 13 | Cerebral atrophy and bilateral cerebellum atrophy | N/A | N/A | N/A | N/A | N/A | Sensory polyneuropathy of lower limbs (involved axon) | N/A | N/A | Decreased vision (right eye 0.15, left eye 0.5, optic nerve damage) |
|  | 14 | Cerebral atrophy | N/A | N/A | N/A | N/A | N/A | Sensorimotor polyneuropathy of four limbs (involved both axon and myelin) | N/A | N/A | N/A |
|  | 15 | Hippocampus atrophy | Normal | N/A | N/A | N/A | N/A | N/A | N/A | N/A | N/A |
|  | 16 | Mild cerebral atrophy | Normal | N/A | N/A | N/A | N/A | N/A | N/A | N/A | Decreased vision (left eye 0.2 right eye 0.6, bilateral optic nerve atrophy) |
| Wang et al. 2019 (4) | 1 | Hyperintensity bilaterally in the cerebellum and in the right basal ganglia: modest atrophy of the cerebrum | N/A | N/A | N/A | N/A | N/A | N/A | N/A | N/A | N/A |
| Wei et al. 2019 | 1 | Atrophy | Normal | N/A | N/A | N/A | N/A | Normal | N/A | N/A | N/A |
|  | 2 | Atrophy | Normal | N/A | N/A | N/A | N/A | Normal | N/A | N/A | N/A |
|  | 3 | Atrophy | Normal | N/A | N/A | N/A | N/A | Normal | N/A | N/A | N/A |
|  | 4 | Normal | Normal | N/A | N/A | N/A | N/A | Normal | N/A | N/A | N/A |
|  | 5 | Normal | Normal | N/A | N/A | N/A | N/A | Peripheral neuropathy | N/A | N/A | N/A |
|  | 6 | Atrophy | Normal | N/A | N/A | N/A | N/A | Normal | N/A | N/A | N/A |
|  | 7 | Atrophy | Normal | N/A | N/A | N/A | N/A | Normal | N/A | N/A | N/A |
|  | 8 | Atrophy | Abnormally high intensity signal on T2-weighted sequences that involved the posterior columns and pyramidal tract between the cervical 3 (C3) and C7 levels | N/A | N/A | N/A | N/A | Normal | N/A | N/A | N/A |
| Wei et al. 2020 | 1 | Mild atrophy | Normal | N/A | N/A | N/A | N/A | Large motor unit potentials with reduced recruitment | N/A | Axonal sensorimotor polyneuropathy | N/A |
| Wen et al. 2020 | 1 | N/A | N/A | N/A | N/A | N/A | N/A | N/A | N/A | N/A | TTE: dilated right ventricle (the inter-ventricular measurement of right ventricle is 48 mm), widened pulmonary artery trunk (immediately above the valve, 33 mm), tricuspid regurgitation (mild), and pulmonary regurgitation (mild–moderate)  Cardiac MRI: impaired biventricular function (right ventricular ejection fraction: 15.6% and left ventricular ejection fraction: 38.9%) with late enhancement at the inferior right ventricular insertion point.  Cardiac catheterization: mean pulmonary artery pressure of 55 mmHg, pulmonary artery wedge pressure of 30 mmHg, pulmonary vascular  resistance of 8.7 woods units and the withered tree  sign. |
| Wu et al. 2017 | 1 | Mild diffuse atrophy of the cerebral cortex | Normal | N/A | N/A | N/A | N/A | N/A | N/A | N/A | Ocular fundus examination: bilateral optic atrophy and pigmentary retinal degeneration in the left eye |
|  | 2 | Mild diffuse atrophy of the cerebral cortex | Normal | N/A | N/A | N/A | N/A | N/A | N/A | N/A | Ocular fundus examination: bilateral optic atrophy |
| Wu et al. 2018 | 1 | 1º MRI: focal hyperintensity in the right occipital white matter on fluid-attenuated inversion recovery  2º MRI: mild expansion of the right occipital white matter lesion  3º MRI: confluent white matter lesions that were distributed asymmetrically in bilateral centrum semiovale and periventricular areas on fluid-attenuated inversion recovery and diffusion-weighted images | N/A | N/A | N/A | N/A | N/A | N/A | N/A | N/A | N/A |
|  | 2 | Normal | N/A | N/A | N/A | N/A | N/A | N/A | N/A | N/A | N/A |
|  | 3 | Normal | N/A | N/A | N/A | N/A | N/A | N/A | N/A | N/A | N/A |
| Zhao et al. 2021 | 1 | N/A | N/A | N/A | N/A | N/A | Computed tomography pulmonary angiogram: diffuse centrilobular ground-glass shadows and nodular shadows, dilated pulmonary artery, and enlarged right ventricle | N/A | N/A | N/A | Pulmonary function test: severe decrease of diffusion function.  Echocardiography: intermediate probability of suspected PH.  Right heart catheterization (RHC) and pulmonary angiography: mean PAP (mPAP) level of 34 mmHg, a pulmonary artery wedge pressure of 10 mmHg, and a pulmonary vascular resistance of 5.8 WU. |
| **Abbreviations:** N/A: Not available, MRI: magnetic resonance imaging, EEG: electroencephalogram, Cardiac MRI: Cardiovascular magnetic resonance imaging, TTE: Transthoracic echocardiogram, EMG: electromyographic exam, MoCA: Montreal Cognitive Assessment | | | | | | | | | | | |

**Table 8. Cell type analysis for diagnosis and genetic diagnosis**

| **Author and year** | **# of cases** | **Cell type analysis for diagnosis** | **Genetic diagnosis** |
| --- | --- | --- | --- |
|  |  |  |  |
| Ardissino et al. 2017 | 1 | N/A | c.271dupA (p.Arg91Lysfs∗14) c.388T>C (p.Tyr130His) |
| Augoustides-Savvopoulou et al. 1999 **^1^** | 1 | Fibroblasts | c.271dupA  c.394C>T |
| Backe et al. 2013 | 1 | Fibroblasts | Homozygous deletion of three base pairs in exon 3 of MMACHC; NM_015506.2:c.392_394delAAC |
| Ben-Omran et al. 2007 | 1 | Fibroblasts | homozygous for the R132X mutation |
|  | 2 | Fibroblasts | homozygous for the R132X mutation |
| Bodamer et al. 2001 **^1^** | 1 | Fibroblasts | c.271dupA c.482G>A |
| Boxer et al. 2005 **^1^** | 1 | Fibroblasts | c.394C>T c.394C>T |
|  | 2 | N/A | N/A |
| Brox-Torrecilla et al. 2021 | 1 | N/A | p.Ser146Argfs*35  p.Arg161Gln |
| Brunelli et al. 2002 | 1 | Fibroblasts | N/A |
| Chang et al. 2020 | 1 | N/A | c.482G>A  c.658-660delAAG |
|  | 2 | N/A | c.482G>A c.609G>A |
|  | 3 | N/A | c.482G>A c.609G>A |
|  | 4 | N/A | c.482G>A, c.658-660delAAG |
|  | 5 | N/A | c.482G>A c.658-660delAAG |
| Chu et al. 2020 | 1 | N/A | Missense mutation of c. 482G > A (VCV000001425.8),  deletion mutation of c.658_660delAAG (VCV000095707.7)  homozygous missense mutations of c.665C>T (VCV000003520.13) in  5,10-methylenetetrahydrofolate reductase (MTHFR) gene. |
|  | 2 | N/A | Missense mutation c. 482G > A (VCV000001425.8), nonsense mutation of c.217C>T (VCV000203825.5) |
|  | 3 | N/A | Missense mutation c. 482G > A (VCV000001425.8), a frameshift mutation of c.440_441del (VCV000203834.4) |
|  | 4 | N/A | Missense mutation c. 482G > A (VCV000001425.8), nonsense mutation of c.609G>A (VCV000030800.6) |
|  | 5 | N/A | Missense mutation c. 482G > A (VCV000001425.8), nonsense mutation of c.609G>A (VCV000030800.6) |
|  | 6 | N/A | deletion mutation c.658_660delAAG (VCV000095707.7), missense mutation of c.347T>C (VCV000001422.3) |
|  | 7 | N/A | Missense mutation of c. 482G > A (VCV000001425.8), nonsense mutation of c.609G>A (VCV000030800.6) |
|  | 8 | N/A | Missense mutation of c. 482G > A (VCV000001425.8), duplication mutation of c.271dupA (VCV000001421.17) |
| Collison et al. 2015 | 1 | N/A | c.G482A:p.Arg161Gln  c.270_271insA:p.Arg91Lysfs*14 |
| Cornec-Legall et al. 2014 | 1 | N/A | c.271dupA  c.389A>G |
| Cui et al. 2019 | 1 | N/A | C.482 G>A  C.271dupA |
|  | 2 | N/A | One pathogenic heterozygous mutation for the disease. |
|  | 3 | N/A | One pathogenic heterozygous mutation for the disease. |
|  | 4 | N/A | missense mutation C.365A>T (p.H122L) and nonsense mutation C.609G>A (p.W203X) |
|  | 5 | N/A | missense mutation C.365A>T (p.H122L) and nonsense mutation C.609G>A (p.W203X) |
| Davin et al. 2009 **^4^** | 1 | N/A | c.276G > T; p.(Glu92Asp)/ erroneous splicing and c.442_444delinsA; p.(Val148MetfsX33) |
| Gerth et al. 2008 | 1 | N/A | c.394C>T  c.394C>T |
|  | 2 | N/A | c.394C>T  c.394C>T |
|  | 3 | N/A | c.3G>A  c.3G>A |
|  | 4 | N/A | c.3G>A  c.3G>A |
| Gilson et al. 2018 | 1 | N/A | p.G1n118fs  p.Arg161Gln |
| Gold et al. 1996 **^2^** | 1 | Fibroblasts | N/A |
|  | 2 | N/A | N/A |
| Goodman et al. 1970 | 1 | Fibroblasts | N/A |
|  | 2 | Fibroblasts | N/A |
| Grandone et al. 2019 | 1 | N/A | p. Tyr130His p.Tyr222Stop |
| Grangé et al. 2015 **^3^** | 1 | N/A | c.271dupA  c.82-12_82-9delTTTC |
|  | 2 | N/A | c.271dupA  c.82-12_82-9delTTTC |
| Guigonis et al. 2005 | 1 | Fibroblasts | c.82-9_12delTTTC  c.271dupA |
|  | 2 | Fibroblasts | c.82-9_12delTTTC  c.271dupA |
| Gündüz et al. 2014 | 1 | N/A | c.484G>T; (p.Gly162Trp) |
| Gurkas et al. 2015 | 1 | N/A | c.394C>T (p.R132X) |
| Heil et al. 2007 | 1 | Fibroblasts | c.1A>G p.Met1?  c.271dupA p.Arg91LysfsX14 duplication |
|  | 2 | Fibroblasts | c.1A>G p.Met1?  c.271dupA p.Arg91LysfsX14 duplication |
| Higashimoto et al. | 1 | N/A | c.271dupA; p.Arg91LysfsX14  c.389A > G; p.Tyr130Cys |
|  | 2 | N/A | c.271dupA; p.Arg91LysfsX14  c.389A > G; p.Tyr130Cys |
|  | 3 | N/A | c.271dupA; p.Arg91LysfsX14  c.389A > G; p.Tyr130Cys |
| Huemer et al. 2014 | 1 | Fibroblasts | c.565C > A (p.R189S) |
|  | 2 | Fibroblasts | c.82-1G > A  c.482G > A |
|  | 3 | Fibroblasts | c.347 T > C |
| Iodice et al. 2013 | 1 | N/A | c.271dupA  c.A389G |
|  | 2 | N/A | c.271dupA  c.A389G |
| Jiménez-Varo et al. 2015 | 1 | N/A | c.271dupA  c.565C>A |
| Kılıç et al. 2013 | 1 | Fibroblasts | c.394C>T |
| Koenig et al. 2015 | 1 | Fibroblasts | c.271dupA; c.276G>T |
| Kömhoff et al. 2013 | 1 | N/A | Post-mortem  c.276G>T/ c.271dupA  p.Glu92Asp/p.Arg91LysfsX14 |
|  | 2 | N/A | c.464G>A/ c.464G>A  p.Gly155Glu/p.Gly155Glu |
|  | 3 | N/A | Post-mortem  c.276G>T/ c.442_444delinsA  p.Glu92Asp/Val148MetfsX33 |
|  | 4 | N/A | c.276G>T/ c.271dupA  p.Glu92Asp/p.Arg91LysfsX14 |
|  | 5 | N/A | c.276G>A/ c.14_24del11  p.Glu92Glu/ p.Val5GlufsX25 |
| Lemoine et al. 2018 | 1 | N/A | c.271dupA  c.82-9_12delTTTC |
|  | 2 | N/A | c.271dupA  c.82-9_12delTTTC |
|  | 3 | N/A | c.271dupA  c.389A>G |
|  | 4 | Fibroblasts | c.271dupA  c.82-9_12delTTTC |
|  | 5 | Fibroblasts | c.271dupA  c.82-9_12delTTTC |
|  | 6 | N/A | c.271dupA  c.82-9_12delTTTC |
|  | 7 | Fibroblasts | c.271dupA  c.389A>G |
| Li et al. 2015 | 1 | N/A | c.609G>A  c.217C>T |
|  | 2 | N/A | c.609G>A  c.365A>T |
|  | 3 | N/A | N/A |
| Lin et al. 2009 | 1 | N/A | R161Q (c.482 G > A, exon 4) and IVS1 + 1 G > A (c.81 + 1 G > A, intron 1) |
| Liu et al. 2015 (1) | 1 | N/A | c.482G>A  c.609G>A |
|  | 2 | N/A | c.482G>A  IVS1, +1G>A |
|  | 3 | N/A | c.482G>A  c.658_660delAAG |
|  | 4 | N/A | c.482G>A  c.609G>A |
|  | 5 | N/A | c.482G>A  c.609G>A |
| Liu et al. 2015 (2) | 1 | N/A | c.315C>G  c.482G>A |
| Liu et al. 2017 | 1 | N/A | c.80A > G (p.Q27R) c.331C > T (p.R111Ter) |
|  | 2 | N/A | N/A |
|  | 3 | N/A | c.80A > G (p.Q27R) c.609G >A (p.W203X) |
|  | 4 | N/A | c.80A >G (p.Q27R) c.609G >A (p.W203X) |
| Liu et al. 2020 | 1 | N/A | c.80A>G (p.Q27R) c.609G>A (p.W203X) |
|  | 2 | N/A | c.80A>G (p.Q27R) c.609G>A (p.W203X) |
|  | 3 | N/A | c.80A>G (p.Q27R) c.609G>A (p.W203X) |
|  | 4 | N/A | c.80A>G (p.Q27R) c.609G>A (p.W203X) |
| Losito et al. 2012 | 1 | N/A | N/A |
| Mitchell et al. 1986 **^1^** | 1 | Fibroblasts | c.271dupA  c.394C>T |
| Navarro et al. 2018 | 1 | N/A | c.271dupA (p.Arg91Lysfs*14)  c.565C>A (p.Arg189Ser) |
| Nogueira et al. 2017 | 1 | N/A | c.394C > T (p.R132*) c.394C > T (p.R132*) |
|  | 2 | N/A | c.271dupA (p.R91Kfs*14) c.394C > T (p.R132*) |
|  | 3 | N/A | c.271dupA (p.R91Kfs*14) c.394C > T (p.R132*) |
|  | 4 | N/A | c.394C > T (p.R132*) c.394C > T (p.R132*) |
|  | 5 | N/A | c.271dupA (p.R91Kfs*14) c.394C > T (p.R132*) |
| Petropoulos et al. 2018 | 1 | N/A | c.271dupA, p.Arg91Lysfs14, heterozygous (autosomal recessive condition) and c.276G>T, p.Glu92Asp, heterozygous (autosomal recessive condition) |
| Philipponnet et al. 2020 | 1 | N/A | Heterozygous pathogenic variants: c.556G > A (p.Arg189His) and c.271dupA (p.Arg91Lysfs*14) |
| Pollini et al. 2020 | 1 | N/A | Heterozygous variant in exon 4: c.482G > A; p.Arg161Gln  splicing variant c.515-1G > T |
| Powers et al. 2001 **^1^** | 1 | Fibroblasts | c.271dupA  c.440G>C |
|  | 2 | Fibroblasts | c.271dupA  c.440G>C |
| Profitlich et al. 2009 | 1 | N/A | C666A/C666A |
| Rahmandar et al. 2014 | 1 | N/A | Compound heterozygote for 2 mutations, c.271dupA and c.482G>A |
| Roze et al. 2003 | 1 | EBV-infected lymphocytes | N/A |
|  | 2 | Fibroblasts  EBV-infected lymphocytes | N/A |
| Shinnar et al. 1984 **^1^** | 1 | Fibroblasts | c.394C > T  c.394C > T |
|  | 2 | Fibroblasts | c.394C > T  c.394C > T |
| Thauvin-Robinet et al. 2007 | 1 | Fibroblasts | Mutations (base pair): C457T and A365G  Mutation (amino acids): R153X and H122R |
|  | 2 | Fibroblasts | Mutations (base pair): 271dupA and C565A  Mutation (amino acids): R91KfsX14 and R189S |
|  | 3 | Fibroblasts | Mutations (base pair): 271dupA and A365G  Mutation (amino acids): R91KfsX14 and H122R |
| Tsai et al. 2007 | 1 | Fibroblasts | c.271dupA and c.482G>A |
| Van Hove et al. 2002 | 1 | Fibroblasts | N/A |
|  | 2 | Fibroblasts | N/A |
| Wang et al. 2012 | 1 | N/A | Heterozygous mutations: c.482G>A and c.609G>A |
|  | 2 | N/A | Heterozygous mutations: c.482G>A and c.1A>G |
|  | 3 | N/A | Heterozygous mutations: c.482G>A and c.609G>A |
| Wang et al. 2018 | 1 | N/A | c.354G>C and c.570insT |
|  | 2 | N/A | c.354G>C and c.570insT |
|  | 3 | N/A | c.445_446del and c.482G>A |
|  | 4 | N/A | c.445_446del and c.482G>A |
|  | 5 | N/A | c.452A>G and c.452A>G |
|  | 6 | N/A | c.452A>G and c.452A>G |
|  | 7 | N/A | c.656_658del and c.482G>A |
|  | 8 | N/A | c.656_658del and c.482G>A |
| Wang et al. 2019 (1) | 1 | N/A | c.609G > A  c.658-660delAAG |
|  | 2 | N/A | c.271dupA  c.609G > A |
|  | 3 | N/A | c.445_446insA  c.609G > A |
|  | 4 | N/A | c.658_660delAAG?  c.609G > A? |
|  | 5 | N/A | c.609G > A  c.626dupT |
|  | 6 | N/A | c.609G > A?  c.658_660delAAG? |
|  | 7 | N/A | c.609G > A  c.394C > T |
|  | 8 | N/A | c.609G > A  c.364dupC |
|  | 9 | N/A | c.482G > A c.658_660delAAG |
|  | 10 | N/A | c.482G > A c.658_660delAAG |
|  | 11 | N/A | c.394C > T c.609G > A |
|  | 12 | N/A | c.609G > A c.482G > A |
|  | 13 | N/A | c.482G > A c.609G > A |
|  | 14 | N/A | c.626dupT c.565C > T |
| Wang et al. 2019 (2) | 1 | N/A | c.217C>T (p.R73*), c.615C>A (p.Y205*) |
|  | 2 | N/A | c.354G>C (p.Q118H), c.570_571insT (p.A191Cfs*12) |
|  | 3 | N/A | c.567dupT (p.1190Yfs* 13), c.482G>A (p.R161Q) |
|  | 4 | N/A | c.482G>A (p.R161Q), c.609G>A (p.W203*) |
|  | 5 | N/A | c.445_446del (p.C149Lfs*16), c.482G>A (p.R161Q) |
|  | 6 | N/A | c.609G>A (p.W203*), c.365A>G (p.H122R) |
|  | 7 | N/A | c463G>C (p.G155R), c.609G>A (p.W203*) |
|  | 8 | N/A | c.482G>A (p.R161Q), c.427C>T (p.Q143*) |
|  | 9 | N/A | c.482G>A (p.R161Q), c.609G>A (p.W203*) |
|  | 10 | N/A | c.609G>A (p.W203*), c.609G>A (p.W203*) |
|  | 11 | N/A | c.609G>A (p.W203*), c.609G>A (p.W203*) |
|  | 12 | N/A | c.609G>A (p.W203*), c.482G>A (p.R161Q) |
|  | 13 | N/A | c.452A>G (p.H151A), c.452A>G (p.H151A) |
|  | 14 | N/A | c.452A>G (p.H151A), c.452A>G (p.H151A) |
|  | 15 | N/A | c.1A>G (p.M1?), c.615C>A (p.Y205*) |
|  | 16 | N/A | c.482G>A (p.R161Q), c.656_658del (p.K220Rfs*71) |
|  | 17 | N/A | c.482G>A (p.R161Q), c.657_659del (p.K220Rfs*71) |
|  | 18 | N/A | c.482G>A (p.R161Q), c.609G>A (p.W203*) |
|  | 19 | N/A | c.80A>G (p.Q27R), c.609G>A (p.W203*) |
|  | 20 | N/A | c.482G>A (p.R161Q), c.609G>A (p.W203*) |
|  | 21 | N/A | c.482G>A (p.R161Q), c.656_658del (p.K220Rfs*71) |
|  | 22 | N/A | c.482G>A (p.R161Q), c.656_658del (p.K220Rfs*71) |
|  | 23 | N/A | c.482G>A (p.R161Q), c.658_660del (p.A221Gfs*71) |
|  | 24 | N/A | c.482G>A (p.R161Q), c.658_660del (p.A221Gfs*71) |
|  | 25 | N/A | c.1A>G (p.M1?), c.445_446del (p.C149Lfs*16) |
|  | 26 | N/A | c.482G>A (p.161Q), c.567dupT (p.1190Yfs*13) |
| Wang et al. 2019 (3) | 1 | N/A | c.482G > A, c.609G > A |
|  | 2 | N/A | c.482G > A, c.567dupT |
|  | 3 | N/A | c.482G > A, c.1A > G |
|  | 4 | N/A | c.482G > A, c.609G > A |
|  | 5 | N/A | c.482G > A, c.609G > A |
|  | 6 | N/A | c.482G > A, c.626dupT |
|  | 7 | N/A | c.567dupT, c.565C > A |
|  | 8 | N/A | c.467G > A, c.482G > A |
|  | 9 | N/A | c.482G > A,c.656_658del |
|  | 10 | N/A | c.482G > A, c.656_658del |
|  | 11 | N/A | c.482G > A, c.427C > T |
|  | 12 | N/A | c.482G > A, c.609G > A |
|  | 13 | N/A | c.482G > A, c.658_660del |
|  | 14 | N/A | c.482G > A, c.609G > A |
|  | 15 | N/A | c.326_329del, .482G >A |
|  | 16 | N/A | c.482G > A, c.609G > A |
| Wang et al. 2019 (4) | 1 | N/A | c.445_446delTG, c.482G>A |
| Wei et al. 2019 | 1 | N/A | c.482G> A  c.609G> A |
|  | 2 | N/A | N/A |
|  | 3 | N/A | c.394C> T  c.565C>A |
|  | 4 | N/A | N/A |
|  | 5 | N/A | c.482G>A  c.658_660 del |
|  | 6 | N/A | c.914 T>C  c.278G>A |
|  | 7 | N/A | c.482G>A  c.656_658del |
|  | 8 | N/A | N/A |
| Wei et al. 2020 | 1 | N/A | c.217 C > T  c.482 G > A |
| Wen et al. 2020 | 1 | N/A | c.80A > G/c.609G > A |
| Wu et al. 2017 | 1 | N/A | c.482G>A and c.658_660del |
|  | 2 | N/A | c.482G>A and c.658_660del |
| Wu et al. 2018 | 1 | N/A | c.609G>A and c.349G>C |
|  | 2 | N/A | c.609G>A and c.349G>C |
|  | 3 | N/A | c.609G>A and c.349G>C |
| Zhao et al. 2021 | 1 | N/A | c.80A>G, p.Gln27Arg and c.394C>T, p.Arg 132 X |
|  | Total: 199 patients | Fibroblasts: 34 patients  EBV-infected lymphocytes: 2 patients  N/A: 164 patients | Genetic diagnosis: 183 patients |
| **Notes:**  **^1^** Genetic mutations taken from Morel et al. 2006  **^2^** Additional data on Patient 1 and Patient 2 were taken from Motte et al. 2019  **^3^** Additional data on Patient 1 and data on Patient 2 were taken from Lemoine et al. 2018  **^4^** Genetic mutations taken from Bouts et al. 2010  **Abbreviations:** N/A: Not available, EBV: Epstein-Barr virus | | | |

**Table 9. Cobalamin C disease treatment**

| **Author and year** | **# of cases** | **Vitamin B12** | **Folic acid** | **Betaine** | **L-carnitine** | **Vitamin B6** | **Diet** | **Other** |
| --- | --- | --- | --- | --- | --- | --- | --- | --- |
| Ardissino et al. 2017 | 1 | Intravenous hydroxocobalamin, 5 mg/day | 5 mg/day | 4 g/day | N/A | N/A | N/A | N/A |
| Augoustides-Savvopoulou et al. 1999 | 1 | Intramuscular hydroxocobalamin, 1 mg/day | Oral  10 mg/day | 6 g/day | 100 mg/kg/day | N/A | N/A | N/A |
| Backe et al. 2013 | 1 | Intramuscular hydroxocobalamin, 1 mg | Oral 4 mg/day | Oral 2 x 6 g | 3 x 3.75 mg | N/A | N/A | Methionine, perorally 3 x 400 mg |
| Ben-Omran et al. 2007 | 1 | Intramuscular hydroxocobalamin, 1 mg | N/A | Oral  250 mg/kg/day | Yes, later weaned off | N/A | Low protein diet; later weaned off | N/A |
|  | 2 | Intramuscular cyanocobalamin 1 mg/day  Intramuscular hydroxocobalamin, 1 mg/day | N/A | Yes | N/A | N/A | N/A | N/A |
| Bodamer et al. 2001 | 1 | Intramuscular hydroxocobalamin, 2 mg/day | N/A | N/A | Oral  80 mg/kg/day | N/A | Low protein diet; later weaned off | N/A |
| Boxer et al. 2005 | 1 | Oral vitamin B12, 1 mg/day  Intramuscular hydroxocobalamin, 1 mg/day over 7 weeks and 2 mg/3 times per week over 6 months | Oral  1 mg/day for 7 weeks and 5 mg/day over 6 months | Oral  6 g/day for 7 weeks and 10 g/twice daily over 6 months | 333 mg three times/day | Oral  50 μg/day over 7 weeks and 100 mg/day over 6 months | Mild protein restriction to 45 to 50 g/day | Vitamin E 400 UI/day  Clopidgrel 75 mg twice daily for 7 weeks  ASA 325 mg/day for 7 weeks and 81 mg/day over 6 months |
|  | 2 | N/A | N/A | N/A | N/A | N/A | N/A | N/A |
| Brox-Torrecilla et al. 2021 | 1 | Intramuscular hydroxocobalamin, 1 mg/day and 2/week | 5 mg/day | 3 g every 8 hours | 20-25 mg/kg/day | N/A | N/A | N/A |
| Brunelli et al. 2002 | 1 | Intramuscular vitamin B12, 1 mg/day over 1 week and then 2000 mg/day orally | N/A | 1.5-1.6 g/day | N/A | N/A | N/A | Methionine: 8 g/day |
| Chang et al. 2020 | 1-5 | Intramuscular hydroxocobalamin, 1 mg/day | Oral  10 mg/day | Oral  2 g/day | Intravenous  3 g/day | N/A | N/A | Compound vitamin B (oral, 40 mg/day) |
| Chu et al. 2020 | 1-8 | Intramuscular adenosylcobalamin, 1 mg/day | 5 mg/day | 9 g/day | Oral  3 g/day | N/A | N/A | Compound vitamin B |
| Collison et al. 2015 | 1 | Intramuscular hydroxocobalamin | N/A | N/A | Yes | N/A | N/A | N/A |
| Cornec-Legall et al. 2014 | 1 | Intramuscular hydroxocobalamin  1 mg/day | 10 mg/day | Oral  9 g/day | N/A | N/A | N/A | N/A |
| Cui et al. 2019 | 1 | Intramuscular injection of vitamin B12 twice a week | N/A | Yes | 1 g/twice a day | N/A | N/A | N/A |
|  | 2 | N/A | N/A | N/A | N/A | N/A | N/A | N/A |
|  | 3 | N/A | N/A | N/A | N/A | N/A | N/A | N/A |
|  | 4 | Methycobal, 500 mg/day | N/A | Yes | 1 g/twice a day | N/A | Normal diet | N/A |
|  | 5 | N/A | N/A | N/A | N/A | N/A | N/A | N/A |
| Davin et al. 2009 | 1 | N/A | N/A | N/A | N/A | N/A | N/A | N/A |
| Gerth et al. 2008 | 1-4 | N/A | N/A | N/A | N/A | N/A | N/A | N/A |
| Gilson et al. 2018 | 1 | Intramuscular hydroxocobalamin, 2 mg/day | 5 mg/day | 3 g/BID | 800 mg every 4 hours | 12.5 mg/day | N/A | Lactulose 15 mL twice a day,  Metronidazole 500 mg 3  times a day |
| Gold et al. 1996 **^1^** | 1 | Intravenous hydroxocobalamin, 500 μg/day  Oral cyanocobalamin, 300 μg/day  Oral hydroxocobalamin, 10 mg/day  Intramuscular hydroxocobalamin, 10 mg/week | N/A | N/A | N/A | N/A | N/A | N/A |
|  | 2 | Intramuscular hydroxocobalamin, 2 mg (single dose). The patient refused additional treatment | N/A | N/A | N/A | N/A | N/A | N/A |
| Goodman et al. 1970 | 1 | N/A | N/A | N/A | N/A | N/A | N/A | N/A |
|  | 2 | N/A | N/A | N/A | N/A | N/A | N/A | N/A |
| Grandone et al. 2019 | 1 | Intramuscular hydroxocobalamin, 1 mg/week and 1 mg every 3 days | Calcium folinate, 15 mg/day | N/A | N/A | N/A | N/A | Low-molecular weight heparin (enoxaparin) at prophylactic doses (4000 IU/day) during the pregnancy |
| Grangé et al. 2015 **^2^** | 1 | Intramuscular hydroxocobalamin | Yes | Oral | N/A | N/A | N/A | Plasma exchange, haemodialysis, intravenous methylprednisolone |
|  | 2 | N/A | N/A | N/A | N/A | N/A | N/A | N/A |
| Guigonis et al. 2005 | 1 | Subcutaneous hydroxycobalamin, 5 mg x 2/mo | Yes | Oral | N/A | N/A | N/A | Plasma exchange |
|  | 2 | Subcutaneous hydroxycobalamin, 5 mg x 2/mo | Yes | Oral | N/A | N/A | N/A | N/A |
| Gündüz et al. 2014 | 1 | Intramuscular hydroxocobalamin, 1 mg/day for 2 weeks, continued twice a week in the first year and once a week in the second year. | N/A | N/A | N/A | N/A | N/A | N/A |
| Gurkas et al. 2015 | 1 | Intramuscular hydroxocobalamin, 1 mg/day | Oral  5 mg/day | Oral  250 mg/kg/day | N/A | N/A | N/A | N/A |
| Heil et al. 2007 | 1 | Intramuscular B12, 1 mg/week | Oral  5 mg/day | N/A | Oral  50 mg/kg/day | Oral  100 mg/day | N/A | N/A |
|  | 2 | Intramuscular B12, 1 mg/week | Oral  5 mg/day | N/A | Oral  50 mg/kg/day | Oral  100 mg/day | N/A | N/A |
| Higashimoto et al. 2019 | 1 | Hydroxocobalamin injection, 1 mg/day (0.02 mg/kg/day) and increased to 25 mg/day (0.5 mg/kg/day) | 1 mg/day | 250 mg/kg/day | N/A | N/A | N/A | Metanx (L-methylfolate, pyridoxal-50-phosphate, and methylcobalamin) |
|  | 2 | Hydroxocobalamin injection, 25 mg/day and later weekly | Oral | Oral | N/A | N/A | N/A | Metanx (L-methylfolate, pyridoxal-50-phosphate, and methylcobalamin) |
|  | 3 | Hydroxocobalamin injection, 25 mg/day and later weekly | Oral | Oral | N/A | N/A | N/A | Metanx (L-methylfolate, pyridoxal-50-phosphate, and methylcobalamin) |
| Huemer et al. 2014 | 1 | Intravenous hydroxocobalamin, daily | 5 mg/day | Oral  16 g/day | 1 g/day | N/A | N/A | N/A |
|  | 2 | Intramuscular hydroxocobalamin, 3×2 mg/week and 3×5 mg/week | 1×5 mg PO/day | 2×6 g PO/day | N/A | N/A | N/A | Methionine, 2×125 mg/day |
|  | 3 | Intravenous hydroxocobalamin, 1000 μg/day for two weeks, then three times a week for the next four weeks, followed by weekly supplementation. | N/A | N/A | N/A | N/A | N/A | N/A |
| Iodice et al. 2013 | 1 | Intramuscular hydroxocobalamin, 1 mg/day | 5 mg/day | 250 mg/kg/day | N/A | N/A | N/A | N/A |
|  | 2 | Hydroxocobalamin | N/A | Yes | N/A | N/A | N/A | N/A |
| Jiménez-Varo et al. 2015 | 1 | Intramuscular hydroxocobalamin, 10 mg/week | N/A | 3 g/day | N/A | 2x300 mg/day | N/A | N/A |
| Kılıç et al. 2013 | 1 | Intramuscular hydroxocobalamin, 1 mg/day for seven days followed by twice weekly | 5 mg/day | 150 mg/kg/day | N/A | N/A | N/A | N/A |
| Koenig et al. 2015 | 1 | Subcutaneous hydroxocobalamin, 5000 μg for 5 days and twice a week thereafter | Oral  0.4 mg every second day | N/A | N/A | N/A | N/A | N/A |
| Kömhoff et al. 2013 | 1 | No treatment | N/A | N/A | N/A | N/A | N/A | N/A |
|  | 2 | Hydroxocobalamin, 1 mg/day for 2 weeks only | N/A | N/A | N/A | N/A | N/A | N/A |
|  | 3 | No treatment | N/A | N/A | N/A | N/A | N/A | N/A |
|  | 4 | Intramuscular hydroxocobalamin, 1 mg/day, 3 times a week | N/A | N/A | N/A | N/A | N/A | N/A |
|  | 5 | Hydroxocobalamin | N/A | N/A | N/A | N/A | N/A | N/A |
| Lemoine et al. 2018 | 1 | Intramuscular hydroxocobalamin | Yes | Yes | N/A | N/A | N/A | Plasma exchange Solumedrol |
|  | 2 | No | No | No | N/A | N/A | N/A | No |
|  | 3 | Intramuscular hydroxocobalamin | Yes | Yes | N/A | N/A | N/A | Eculizumab |
|  | 4 | Subcutaneous hydroxocobalamin | Yes | Yes | N/A | N/A | N/A | Plasma exchange |
|  | 5 | Subcutaneous hydroxocobalamin | Yes | Yes | N/A | N/A | N/A | No |
|  | 6 | Intramuscular hydroxocobalamin | Yes | Yes | N/A | N/A | N/A | Plasma exchange |
|  | 7 | Intramuscular hydroxocobalamin | Yes | Yes | N/A | N/A | N/A | Plasma exchange |
| Li et al. 2015 | 1 | Intramuscular injection of vitamin B12, 0.5-1 mg/day once or twice weekly | 2.5-5 mg/day | 500-2000 mg/day | 500 mg/day | N/A | N/A | N/A |
|  | 2 | Intramuscular injection of vitamin B12, 0.5-1 mg/day once or twice weekly | 2.5-5 mg/day | 500-2000 mg/day | 500 mg/day | N/A | N/A | N/A |
|  | 3 | Intravenous hydroxocobalamin | N/A | N/A | N/A | N/A | N/A | N/A |
| Lin et al. 2009 | 1 | Intramuscular hydroxocobalamin daily | N/A | N/A | N/A | N/A | N/A | N/A |
| Liu et al. 2015 (1) | 1-5 | Intravenous methylcobalamin, 0.5–1 mg/day  Oral methylcobalamin, 1.5–3 mg/day | Oral  5 mg/day | Oral,  3–6 g/day | N/A | N/A | N/A | N/A |
| Liu et al. 2015 (2) | 1 | Intramuscular cobalamin mixture, 1 mg, twice per week  During the pregnancy → Intramuscular hydroxocobalamin, 1 mg/day | Calcium folinate, oral, 15 mg/day  During the pregnancy → folic acid oral, 5 mg/day | Oral 2 g/day  During the pregnancy → Oral 1 g/day | Oral 2 g/day  During the pregnancy → Oral 1 g/day | N/A | Normal diet | N/A |
| Liu et al. 2017 | 1 | Intramuscular cyanocobalamin, 0.5 mg/day | Oral  5 mg/day | Oral 250 mg/kg/day | Oral  100 mg/kg/day | Oral 10 mg/kg twice daily | N/A | Oral captopril 3.125 mg twice daily |
|  | 2 | Intravenous cyanocobalamin | N/A | Yes | Yes | Yes | N/A | N/A |
|  | 3 | Intravenous cyanocobalamin | Yes | Yes | Yes | Yes | N/A | N/A |
|  | 4 | Intravenous cyanocobalamin | Yes | Yes | Yes | Yes | N/A | N/A |
| Liu et al. 2020 | 1 | Hydroxocobalamin | Yes | Yes | Yes | Yes | N/A | Sildenafil and bosentan |
|  | 2 | Hydroxocobalamin | Yes | Yes | Yes | Yes | N/A | Sildenafil and bosentan |
|  | 3 | Hydroxocobalamin | Yes | Yes | Yes | Yes | N/A | Sildenafil and bosentan |
|  | 4 | Hydroxocobalamin | Yes | Yes | Yes | Yes | N/A | Sildenafil and bosentan |
| Losito et al. 2012 | 1 | N/A | N/A | N/A | N/A | N/A | N/A | Warfarin and continuous intravenous infusion of epoprostenol (8 μg/kg/min)  After 18 months → Bosentan (125 mg b.i.d.) and prostacyclin treatment discontinued.  IECA + Amlodipino |
| Mitchell et al. 1986 | 1 | Intramuscular cyanocobalamin, 5 μg/day for 5 days and 500 μg/day for 6 days  Intramuscular hydroxocobalamin, 1 mg/day; 1 mg/month; 1 mg/3 weeks | N/A | N/A | N/A | N/A | Normal diet | N/A |
| Navarro et al. 2018 | 1 | Intramuscular hydroxocobalamin  5 mg, three times a week | 10 mg/day | N/A | 3 g/day | N/A | N/A | N/A |
| Nogueira et al. 2017 | 1-5 | N/A | N/A | N/A | N/A | N/A | N/A | N/A |
| Petropoulos et al. 2018 | 1 | Intramuscular hydroxocobalamin  1 mg | 5 mg/TID | 3 g/TID | 660 mg/TID | N/A | N/A | N/A |
| Philipponnet et al. 2020 | 1 | Intramuscular hydroxocobalamin  1 mg/day | 10 mg/day | 12 g/day | 3g/day | N/A | N/A | Rituximab infusion at 375 mg/m2 |
| Pollini et al. 2020 | 1 | Hydroxycobalamin, 5000 μg on alternate days | 15 mg/day | 6 g/day | 6 g/day | N/A | N/A | N/A |
| Powers et al. 2001 | 1-2 | N/A | N/A | N/A | N/A | N/A | N/A | N/A |
| Profitlich et al. 2009 | 1 | Hydroxocobalamin | N/A | Yes | N/A | N/A | Protein restriction | Aspirin |
| Rahmandar et al. 2014 | 1 | Intramuscular hydroxocobalamin  1 mg/day | Oral 5 mg/day | Oral 250 mg/kg/day | N/A | N/A | N/A | N/A |
| Roze et al. 2003 | 1 | Intramuscular hydroxocobalamin 1 mg | 10 mg | Oral 9 g | 3 g | N/A | N/A | N/A |
|  | 2 | Intravenous hydroxocobalamin 2 mg | 10 mg | Oral 9 g | 3 g | N/A | N/A | N/A |
| Shinnar et al. 1984 | 1 | Intravenous hydroxocobalamin, 1000 μg/day | 3 mg/day for 4 weeks | N/A | N/A | N/A | N/A | N/A |
|  | 2 | Intravenous hydroxocobalamin | N/A | N/A | N/A | N/A | N/A | N/A |
| Thauvin-Robinet et al. 2007 | 1 | Intramuscular hydroxocobalamin, 1 mg  Oral hydroxocobalamin  Intravenous hydroxocobalamin, 2 mg | Oral 10 mg | Oral 6 g | N/A | N/A | N/A | N/A |
|  | 2 | Intravenous hydroxocobalamin | Oral 10 mg | Oral 9 g | N/A | N/A | N/A | N/A |
|  | 3 | Intramuscular hydroxocobalamin, 1 mg  Oral hydroxocobalamin, 1 mg/day  Intravenous hydroxocobalamin, 2.5 g/month  Intracutaneous hydroxocobalamin | Oral 10 mg | Oral 16 g | N/A | N/A | N/A | N/A |
| Tsai et al. 2007 | 1 | Intramuscular hydroxocobalamin 1 mg | Oral 5 mg | 3 g/TID | N/A | N/A | N/A | N/A |
| Van Hove et al. 2002 | 1 | Intramuscular hydroxocobalamin  1 mg; 2.5 mg/day; 5 mg/day  Subcutaneous hydroxocobalamin | Oral 3 mg/day | Oral 150 mg/kg/day | N/A | N/A | N/A | N/A |
|  | 2 | Intramuscular hydroxocobalamin, 1 mg/day; 2.5 mg/day; 5 mg/day | Oral 3 mg/day | Oral 150 mg/kg/day | N/A | N/A | N/A | N/A |
| Wang et al. 2012 | 1 | Intravenous hydroxocobalamin 1 mg/day | Oral 5 mg/TID | N/A | N/A | N/A | N/A | N/A |
|  | 2 | Intravenous hydroxocobalamin 1 mg/day | Oral 5 mg/TID | N/A | N/A | N/A | N/A | N/A |
|  | 3 | Intravenous hydroxocobalamin 1 mg/day | N/A | N/A | N/A | N/A | N/A | N/A |
| Wang et al. 2018 | 1 | Intramuscular cyanocobalamin, 0.5-1 mg/day  Intravenous/intramuscular methylcobalamin, 0.5-1 mg/day  Intramuscular cyanocobalamin, 1–2 mg/week  Oral methylcobalamin, 1–1.5 mg/day | Oral 10-15 mg/day | Oral 0.5–2 g/day | Intravenous infusion/oral  1–3 g/day | N/A | N/A | Compound vitamin B (oral, 20–60 mg/day) |
|  | 2 | N/A | N/A | N/A | N/A | N/A | N/A | N/A |
|  | 3 | Intramuscular cyanocobalamin, 0.5-1 mg/day  Intravenous/intramuscular methylcobalamin, 0.5-1 mg/day  Intramuscular cyanocobalamin, 1–2 mg/week  Oral methylcobalamin, 1–1.5 mg/day | Oral 10-15 mg/day | Oral 0.5–2 g/day | Intravenous infusion/oral  1–3 g/day |  |  | Compound vitamin B (oral, 20–60 mg/day) |
|  | 4 | Intramuscular cyanocobalamin, 0.5-1 mg/day  Intravenous/intramuscular methylcobalamin, 0.5-1 mg/day  Intramuscular cyanocobalamin, 1–2 mg/week  Oral methylcobalamin, 1–1.5 mg/day | Oral 10-15 mg/day | Oral 0.5–2 g/day | Intravenous infusion/oral  1–3 g/day | N/A | N/A | Compound vitamin B (oral, 20–60 mg/day) |
|  | 5 | Intramuscular cyanocobalamin, 0.5-1 mg/day  Intravenous/intramuscular methylcobalamin, 0.5-1 mg/day  Intramuscular cyanocobalamin, 1–2 mg/week  Oral methylcobalamin, 1–1.5 mg/day | Oral 10-15 mg/day | Oral 0.5–2 g/day | Intravenous infusion/oral  1–3 g/day | N/A | N/A | Compound vitamin B (oral, 20–60 mg/day) |
|  | 6 | Intramuscular cyanocobalamin, 0.5-1 mg/day  Intravenous/intramuscular methylcobalamin, 0.5-1 mg/day  Intramuscular cyanocobalamin, 1–2 mg/week  Oral methylcobalamin, 1–1.5 mg/day | Oral 10-15 mg/day | Oral 0.5–2 g/day | Intravenous infusion/oral  1–3 g/day | N/A | N/A | Compound vitamin B (oral, 20–60 mg/day) |
|  | 7 | Intramuscular cyanocobalamin, 0.5-1 mg/day  Intravenous/intramuscular methylcobalamin, 0.5-1 mg/day  Intramuscular cyanocobalamin, 1–2 mg/week  Oral methylcobalamin, 1–1.5 mg/day | Oral 10-15 mg/day | Oral 0.5–2 g/day | Intravenous infusion/oral  1–3 g/day | N/A | N/A | Compound vitamin B (oral, 20–60 mg/day) |
|  | 8 | Intramuscular cyanocobalamin, 0.5-1 mg/day  Intravenous/intramuscular methylcobalamin, 0.5-1 mg/day  Intramuscular cyanocobalamin, 1–2 mg/week  Oral methylcobalamin, 1–1.5 mg/day | Oral 10-15 mg/day | Oral 0.5–2 g/day | Intravenous infusion/oral  1–3 g/day | N/A | N/A | Compound vitamin B (oral, 20–60 mg/day) |
| Wang et al. 2019 (1) | 1 | N/A | N/A | N/A | N/A | N/A | N/A | N/A |
|  | 2 | N/A | N/A | N/A | N/A | N/A | N/A | N/A |
|  | 3 | N/A | N/A | N/A | N/A | N/A | N/A | N/A |
|  | 4 | N/A | N/A | N/A | N/A | N/A | N/A | N/A |
|  | 5 | N/A | N/A | N/A | N/A | N/A | N/A | N/A |
|  | 6 | N/A | N/A | N/A | N/A | N/A | N/A | N/A |
|  | 7 | N/A | N/A | N/A | N/A | N/A | N/A | N/A |
|  | 8 | N/A | N/A | N/A | N/A | N/A | N/A | N/A |
|  | 9 | N/A | N/A | N/A | N/A | N/A | N/A | N/A |
|  | 10 | N/A | N/A | N/A | N/A | N/A | N/A | N/A |
|  | 11 | N/A | N/A | N/A | N/A | N/A | N/A | N/A |
|  | 12 | N/A | N/A | N/A | N/A | N/A | N/A | N/A |
|  | 13 | N/A | N/A | N/A | N/A | N/A | N/A | N/A |
|  | 14 | N/A | N/A | N/A | N/A | N/A | N/A | N/A |
| Wang et al. 2019 (2) **^3^** | 1 | Intramuscular cyanocobalamin, 0.5-1 mg/day  and/or  Intramuscular hydroxocobalamin, 0.5-1 mg/day | Oral  10-30 mg | Oral  1-3 g | Intravenous  1–3 g/day | N/A | N/A | Compound vitamin B (oral, 20–60 mg/day |
|  | 2 | Intramuscular cyanocobalamin, 0.5-1 mg/day  and/or  Intramuscular hydroxocobalamin, 0.5-1 mg/day | Oral  10-30 mg | Oral  1-3 g | N/A | N/A | N/A | N/A |
|  | 3 | Intramuscular cyanocobalamin, 0.5-1 mg/day  and/or  Intramuscular hydroxocobalamin, 0.5-1 mg/day | Oral  10-30 mg | Oral  1-3 g | N/A | N/A | N/A | N/A |
|  | 4 | Intramuscular cyanocobalamin, 0.5-1 mg/day  and/or  Intramuscular hydroxocobalamin, 0.5-1 mg/day | Oral  10-30 mg | Oral  1-3 g | N/A | N/A | N/A | N/A |
|  | 5 | Intramuscular cyanocobalamin, 0.5-1 mg/day  and/or  Intramuscular hydroxocobalamin, 0.5-1 mg/day | Oral  10-30 mg | Oral  1-3 g | N/A | N/A | N/A | N/A |
|  | 6 | Intramuscular cyanocobalamin, 0.5-1 mg/day  and/or  Intramuscular hydroxocobalamin, 0.5-1 mg/day | Oral  10-30 mg | Oral  1-3 g | N/A | N/A | N/A | N/A |
|  | 7 | Intramuscular cyanocobalamin, 0.5-1 mg/day  and/or  Intramuscular hydroxocobalamin, 0.5-1 mg/day | Oral  10-30 mg | Oral  1-3 g | N/A | N/A | N/A | N/A |
|  | 8 | Intramuscular cyanocobalamin, 0.5-1 mg/day  and/or  Intramuscular hydroxocobalamin, 0.5-1 mg/day | Oral  10-30 mg | Oral  1-3 g | N/A | N/A | N/A | N/A |
|  | 9 | Intramuscular cyanocobalamin, 0.5-1 mg/day  and/or  Intramuscular hydroxocobalamin, 0.5-1 mg/day | Oral  10-30 mg | Oral  1-3 g | N/A | N/A | N/A | N/A |
|  | 10 | Intramuscular cyanocobalamin, 0.5-1 mg/day  and/or  Intramuscular hydroxocobalamin, 0.5-1 mg/day | Oral  10-30 mg | Oral  1-3 g | N/A | N/A | N/A | N/A |
|  | 11 | Intramuscular cyanocobalamin, 0.5-1 mg/day  and/or  Intramuscular hydroxocobalamin, 0.5-1 mg/day | Oral  10-30 mg | Oral  1-3 g | N/A | N/A | N/A | N/A |
|  | 12 | Intramuscular cyanocobalamin, 0.5-1 mg/day  and/or  Intramuscular hydroxocobalamin, 0.5-1 mg/day | Oral  10-30 mg | Oral  1-3 g | N/A | N/A | N/A | N/A |
|  | 13 | Intramuscular cyanocobalamin, 0.5-1 mg/day  and/or  Intramuscular hydroxocobalamin, 0.5-1 mg/day | Oral  10-30 mg | Oral  1-3 g | N/A | N/A | N/A | N/A |
|  | 14 | Intramuscular cyanocobalamin, 0.5-1 mg/day  and/or  Intramuscular hydroxocobalamin, 0.5-1 mg/day | No | Oral  1-3 g | N/A | N/A | N/A | N/A |
|  | 15 | Intramuscular cyanocobalamin, 0.5-1 mg/day  and/or  Intramuscular hydroxocobalamin, 0.5-1 mg/day | Oral  10-30 mg | Oral  1-3 g | N/A | N/A | N/A | N/A |
|  | 16 | Intramuscular cyanocobalamin, 0.5-1 mg/day  and/or  Intramuscular hydroxocobalamin, 0.5-1 mg/day | No | Oral  1-3 g | N/A | N/A | N/A | N/A |
|  | 17 | Intramuscular cyanocobalamin, 0.5-1 mg/day  and/or  Intramuscular hydroxocobalamin, 0.5-1 mg/day | Oral  10-30 mg | Oral  1-3 g | N/A | N/A | N/A | N/A |
|  | 18 | Intramuscular cyanocobalamin, 0.5-1 mg/day  and/or  Intramuscular hydroxocobalamin, 0.5-1 mg/day | No | Oral  1-3 g | N/A | N/A | N/A | N/A |
|  | 19 | Intramuscular cyanocobalamin, 0.5-1 mg/day  and/or  Intramuscular hydroxocobalamin, 0.5-1 mg/day | No | Oral  1-3 g | N/A | N/A | N/A | N/A |
|  | 20 | Intramuscular cyanocobalamin, 0.5-1 mg/day  and/or  Intramuscular hydroxocobalamin, 0.5-1 mg/day | Oral  10-30 mg | Oral  1-3 g | N/A | N/A | N/A | N/A |
|  | 21 | Intramuscular cyanocobalamin, 0.5-1 mg/day  and/or  Intramuscular hydroxocobalamin, 0.5-1 mg/day | Oral  10-30 mg | Oral  1-3 g | N/A | N/A | N/A | N/A |
|  | 22 | Intramuscular cyanocobalamin, 0.5-1 mg/day  and/or  Intramuscular hydroxocobalamin, 0.5-1 mg/day | No | Oral  1-3 g | N/A | N/A | N/A | N/A |
|  | 23 | Intramuscular cyanocobalamin, 0.5-1 mg/day  and/or  Intramuscular hydroxocobalamin, 0.5-1 mg/day | Oral  10-30 mg | Oral  1-3 g | N/A | N/A | N/A | N/A |
|  | 24 | Intramuscular cyanocobalamin, 0.5-1 mg/day  and/or  Intramuscular hydroxocobalamin, 0.5-1 mg/day | No | Oral  1-3 g | N/A | N/A | N/A | N/A |
|  | 25 | Intramuscular cyanocobalamin, 0.5-1 mg/day  and/or  Intramuscular hydroxocobalamin, 0.5-1 mg/day | Oral  10-30 mg | Oral  1-3 g | N/A | N/A | N/A | N/A |
|  | 26 | Intramuscular cyanocobalamin, 0.5-1 mg/day  and/or  Intramuscular hydroxocobalamin, 0.5-1 mg/day | Oral  10-30 mg | Oral  1-3 g | N/A | N/A | N/A | N/A |
| Wang et al. 2019 (3) | 1-16 | Intravenous hydroxocobalamin | Yes | Yes | Yes | N/A | N/A | N/A |
| Wang et al. 2019 (4) | 1 | N/A | N/A | N/A | N/A | N/A | N/A | N/A |
| Wei et al. 2019 | 1 | Intramuscular cyanocobalamin, 1 mg/day for 1 week followed by a half-dose for 3 weeks | Oral | Oral | Oral | N/A | N/A | N/A |
|  | 2 | Intramuscular hydroxocobalamin, 1 mg/day for 1 week followed by a half-dose for 3 weeks | Oral | Oral | Oral | N/A | N/A | N/A |
|  | 3 | Intramuscular hydroxocobalamin, 1 mg/day for 1 week followed by a half-dose for 3 weeks | Oral | Oral | Oral | N/A | N/A | N/A |
|  | 4 | Intramuscular cyanocobalamin, 1 mg/day for 1 week followed by a half-dose for 3 weeks | Oral | Oral | Oral | N/A | N/A | N/A |
|  | 5 | Intramuscular hydroxocobalamin, 1 mg/day for 1 week followed by a half-dose for 3 weeks | Oral | Oral | Oral | N/A | N/A | N/A |
|  | 6 | Intramuscular hydroxocobalamin, 1 mg/day for 1 week followed by a half-dose for 3 weeks | Oral | Oral | Oral | N/A | N/A | N/A |
|  | 7 | Intramuscular hydroxocobalamin, 1 mg/day for 1 week followed by a half-dose for 3 weeks | Oral | Oral | Oral | N/A | N/A | N/A |
|  | 8 | Intramuscular cyanocobalamin, 1 mg/day for 1 week followed by a half-dose for 3 weeks | Oral | Oral | Oral | N/A | N/A | N/A |
| Wei et al. 2020 | 1 | N/A | N/A | N/A | N/A | N/A | N/A | N/A |
| Wen et al. 2020 | 1 | Intramuscular hydroxocobalamin, 1 mg/day | Oral  5 mg/TID | Oral  1000 mg/day | Oral  10 ml/ BID | N/A | N/A | Sildenafil (20 mg, peros, t.i.d.)  Bosentan (62.5 mg, peros, b.i.d.)  Spironolactone (20 mg, peros, b.i.d.)  Hydrochlorothiazide (25 mg, peros, b.i.d.) w |
| Wu et al. 2017 | 1 -2 | Intramuscular MeCbl, 1 mg/day and 2 mg/week | Oral  5 mg/day | Oral  3 g/day | Intravenous  1 g/day | Oral  30 mg/day | N/A | N/A |
| Wu et al. 2018 | 1-3 | N/A | N/A | N/A | N/A | N/A | N/A | N/A |
| Zhao et al. 2021 | 1 | Vitamin B12 | Yes | Yes | Yes | N/A | N/A | N/A |
|  |  | Hydroxocobalamin: 117 patients: i.v: 30 patients, i.m: 75, N/A: 12 patients  Intramuscular or intravenous cyanocobalamin: 42 patients  Oral vitamin B12 supplementation: 5 patients  Methylcobalamin, cobalamin mixture or adenosylcobalamin: 31 patients  Not treated: 3 patients  N/A: 40 patients | Treatment with folic acid: 127 patients | Treatment with betaine: 134 patients | Treatment with L-carnitine: 79 patients | Treatment with vitamin B6: 15 patients | Normal diet: 3 patients  Low -protein diet: 4 patients |  |
| **Notes:**  **^1^** Additional data on Patient 1 and Patient 2 were taken from Motte et al. 2019  **^2^** Additional data on Patient 1 and data on Patient 2 were taken from Lemoine et al. 2018  **^3^** Unclear how many patients took intravenous L-carnitine 1–3 g/day and compound vitamin B (20–60 mg/day). The data was included just for one patient.  **Abbreviations:** N/A: Not available | | | | | | | | |

**Table 10. Cobalamin C disease patients outcome and follow-up**

| **Author and year** | **# of cases** | **Biochemical and clinical response to treatment** | **General outcome** | **Follow-up** |
| --- | --- | --- | --- | --- |
| Ardissino et al. 2017 | 1 | N/A | Complete recovery | N/A |
| Augoustides-Savvopoulou et al. 1999 | 1 | Disease markers decreased | Improved | 1 year |
| Backe et al. 2013 | 1 | Gradual decrease in disease markers. | Improved. Wheelchair bound. | 1 month, 1 year, and 2 years |
| Ben-Omran et al. 2007 | 1 | Total plasma homocysteine decreased. | Improved, except anxiety symptoms and gait sequelae. | 6 years |
|  | 2 | Excellent clinical and biochemical response. | Improved. Persistent significant learning problems. | 4 years |
| Bodamer et al. 2001 | 1 | MMA decreased and homocysteine normalized. | Improved. Residual diplegia and neurogenic bladder and bowel difficulties. | 5 years |
| Boxer et al. 2005 | 1 | Decreased MMA and homocysteine. | Improved. Seizure-free. | 7 months |
|  | 2 | N/A | Died at the age of 17. |  |
| Brox-Torrecilla et al. 2021 | 1 | Methylmalonic acid levels normalized, elevated homocysteine levels. | Partial improvement of neurological manifestations. Received a kidney transplant. Wheelchair bound. | 3 years |
| Brunelli et al. 2002 | 1 | Disease markers decreased | At the age of 21 years, he had a sudden change in mental status, became deeply comatose, and died from the cerebrovascular complications of the cbl C disease. Plasma homocysteine was 61 nmol/ml. | 39 months |
| Chang et al. 2020 | 1-5 | Serum homocysteine decreased. | Improved. | 3–4 weeks  1 year |
| Chu et al. 2020 | 1-8 | Homocysteine and methylmalonic acid normalized. | Improved. | 2–8 weeks |
| Collison et al. 2015 | 1 | N/A | N/A | N/A |
| Cornec-Legall et al. 2014 | 1 | Disease markers decreased | Recovered. Cessation of hemodialysis therapy. | 3 months |
| Cui et al. 2019 | 1 | N/A | Improved. Abnormal gait. | 2 months and 2 years |
|  | 2 | N/A | Asymptomatic | N/A |
|  | 3 | N/A | Asymptomatic | N/A |
|  | 4 | N/A | Recovered | 6 months |
|  | 5 | N/A | Asymptomatic | N/A |
| Davin et al. 2009 | 1 | N/A | N/A | N/A |
| Gerth et al. 2008 | 1 | N/A | Symptoms resolved, residual peripheral neuropathy | N/A |
|  | 2 | N/A | Developmental improvement, seizures controlled | N/A |
|  | 3 | N/A | Developmental and behavioral progress | N/A |
|  | 4 | N/A | Developmental and behavioral improvement | N/A |
| Gilson et al. 2018 | 1 | Homocysteine levels diminished. | Improved. | N/A |
| Gold et al. 1996 | 1 | MMA decreased. Stable metabolic control. | Improved. Persistent bladder control problems. Spastic paraparesis. The patient had six pregnancies and gave birth to three healthy children. | 5 years, 23 years |
|  |  | Single dose of treatment led to a 60% reduction of MMA. | Asymptomatic. She is married, has three healthy children, and has no subjective clinical complaints. | 26 years |
| Goodman et al. 1970 | 1 | N/A | Improved. | N/A |
|  | 2 | N/A | N/A | N/A |
| Grandone et al. 2019 | 1 | Homocysteine levels normalized. | Recovered. Successful second pregnancy carried out. | N/A |
| Grangé et al. 2015 | 1 | Homocysteine levels normalized. | Recovered. Cessation of hemodialysis therapy. | 5 months |
|  | 2 | N/A | Died at 18 years old, from pulmonary veno-occlusive disease associated with hypertrophic cardiomyopathy. The diagnosis was made 15 years later, when his brother (patient 1) was diagnosed. | N/A |
| Guigonis et al. 2005 | 1 | Disease markers decreased. | Improved. Residual proteinuria, chronic renal failure | Yes |
|  | 2 | Disease markers decreased. | Improved. Residual proteinuria | Yes |
| Gündüz et al. 2014 | 1 | Disease markers decreased. | Recovered at the last examination at the age of 3½years. | 2 years |
| Gurkas et al. 2015 | 1 | N/A | Recovered. | 1 year, 2 years |
| Heil et al. 2007 | 1 | Disease markers decreased. | Recovered. | 1 year |
|  | 2 | Disease markers decreased. | Recovered, but the patient still experienced extreme hyperactivity and concentration problems, increased appetite and overweight | 1 year |
| Higashimoto et al. 2019 | 1 | Disease markers normalized. | Recovered | 4 months |
|  | 2-3 | Disease markers normalized. | N/A | 3 weeks |
| Huemer et al. 2014 | 1 | Disease markers decreased. | Recovered. | 5 years |
|  | 2 | Disease markers decreased. | Recovered. Residues of myelopathy. | 4 years |
|  | 3 | Disease markers normalized. | Recovered. Persistent neuropsychological deficits including confusion and amnesia. | 2 months |
| Iodice et al. 2013 | 1 | Disease markers decreased. | Death | N/A |
|  | 2 | Disease markers normalized. | Recovered. | N/A |
| Jiménez-Varo et al. 2015 | 1 | Disease markers decreased. | Recovered. | N/A |
| Kılıç et al. 2013 | 1 | Disease markers decreased. | Improved. Mild spastic ataxic gait and bilateral extensor plantar responses. | 1 year |
| Koenig et al. 2015 | 1 | Disease markers decreased. | Recovered. | N/A |
| Kömhoff et al. 2013 | 1 | N/A | Died of right ventricular failure | 12 days |
|  | 2 | N/A | Died of right ventricular failure | 14 days |
|  | 3 | N/A | Renal transplantation. Died of right ventricular failure | 7 years |
|  | 4 | N/A | Chronic kidney disease II/ WHO FC, PAH World Health Organization functional class IV | 3.7 years |
|  | 5 | N/A | Chronic kidney disease II/ WHO FC, PAH World Health Organization functional class II | 14 years |
| Lemoine et al. 2018 | 1 | N/A | Improved. Stop hemodialysis | N/A |
|  | 2 | N/A | The patient was not treated and died of pulmonary veinoocclusive disease at 18 years of age. | N/A |
|  | 3 | N/A | Improved. Stop hemodialysis | N/A |
|  | 4 | N/A | Improved. Stop hemodialysis.  This patient relapsed 5 years after treatment initiation because of inobservance | 5 years |
|  | 5 | N/A | Improved. Stop hemodialysis | N/A |
|  | 6 | N/A | Improved. The patient received a kidney transplant 1 year after the diagnosis. | 1 year |
|  | 7 | N/A | Imoroved. She received a second kidney transplant 6 years after the diagnosis. | 6 years |
| Li et al. 2015 | 1 | Disease markers normalized. | Improved. | 1.75 years |
|  | 2 | Disease markers normalized. | Improved. | 1 year |
|  | 3 | The level of MMA in urine declined after treatment. | The patient died of multiple organ failure. | N/A |
| Lin et al. 2009 | 1 | Disease markers normalized. | Improved. | N/A |
| Liu et al. 2015 (1) | 1 | Disease markers decreased. | Improved. Gait abnormalities | 1 year |
|  | 2 | Disease markers decreased. | Improved. Gait abnormalities | 1 year |
|  | 3 | Disease markers decreased. | Improved. Gait abnormalities | 1 year |
|  | 4 | Disease markers decreased. | Improved. Gait abnormalities | 1 year |
|  | 5 | Disease markers decreased. | Improved. | 1 year |
| Liu et al. 2015 (2) | 1 | Disease markers decreased. | Recovered. At the age of 23 years, she became pregnant. Uneventful pregnancy. The c.482G>A mutation was detected on the MMACHC gene in the infant. | 6 months, 1 year, 4 years, 7 years, and 8 years |
| Liu et al. 2017 | 1 | N/A | Asymptomatic | 3 years |
|  | 2 | N/A | Improved initially. Died suddenly | 6 months |
|  | 3 | N/A | Improved | 9 months |
|  | 4 | N/A | Improved | 1 mont |
| Liu et al. 2020 | 1-4 | N/A | Asymptomatic | 1 year |
| Losito et al. 2012 | 1 | N/A | Diagnosed with cblC deficiency 18 years after initial presentation. | N/A |
| Mitchell et al. 1986 | 1 | N/A | Improved. With sequelae of emotional lability and slight lower limb hyperreflexia. | 7.5 years |
| Navarro et al. 2018 | 1 | N/A | Dialysis-dependent. Considered for renal transplantation. | 1 year |
| Nogueira et al. 2017 | 1-5 | N/A | N/A | N/A |
| Petropoulos et al. 2018 | 1 | N/A | Recovered. Off dialysis. | 6 months |
| Philipponnet et al. 2020 | 1 | Disease markers normalized. | Recovered. | 6 months |
| Pollini et al. 2020 | 1 | Disease markers decreased. | Improvement of spastic paresis with restoration of autonomous gait.  At the age of 17, he complained an acute loss of strength and paresthesia in the right arm. The diagnosis of relapsing-remitting MS was definitively made in comorbidity with cblC defect. | N/A |
| Powers et al. 2001 | 1 | N/A | Died at 34 | N/A |
|  | 2 | N/A | Died at 45 | N/A |
| Profitlich et al. 2009 | 1 | N/A | N/A | N/A |
| Rahmandar et al. 2014 | 1 | Disease markers decreased. | Improved. Intermittent adherence to the prescribed regimen | 6 months |
| Roze et al. 2003 | 1 | Disease markers decreased. | Partial recovery, persistent moderate myelopathy. The patient had recurrent miscarriages, probably secondary to placental thrombosis. She also had two uneventful pregnancies with anticoagulation therapy, leading to two healthy children. | 5 years |
|  | 2 | Disease markers decreased. | Improved. Full recovery of the mental and upper extremity function; residual paraplegia requiring wheelchair use. Four and a half years after initiation of hydroxocobalamin therapy, she presented with severe neuropathy and myelopathy. | 4.5 years |
| Shinnar et al. 1984 | 1 | Disease markers decreased. | Improved. Persistent myelopathy. | 4 months |
|  | 2 | Disease markers normalized. | Asymptomatic. | N/A |
| Thauvin-Robinet et al. 2007 | 1 | Disease markers decreased. | Died suddenly at home a few months later | N/A |
|  | 2 | N/A | Recovered | 6 months |
|  | 3 | Variations of plasma homocysteine levels because of the changes in treatment. | During the 2 years after introduction of this treatment, she did not present any recurrence of thromboembolic complications | 2 years |
| Tsai et al. 2007 | 1 | Homocysteine and methylmalonic acid levels remain elevated due to suboptimal compliance. | Wheelchair-bound. Emotionally stable with use of an antidepressant | 2 years |
| Van Hove et al. 2002 | 1 | Reduced total homocysteine levels | Neurologic and psychomotor functions have been excellent. | 1 year |
|  | 2 | Reduced total homocysteine levels | Neurologic and psychomotor functions have been excellent | 1 year |
| Wang et al. 2012 | 1 | N/A | Recovered | N/A |
|  | 2 | N/A | Recovered | N/A |
|  | 3 | N/A | Weakness in the patient's legs did not improve. | N/A |
| Wang et al. 2018 | 1, 3-8 | N/A | Improved | 1-3 years |
|  | 2 | N/A | Asymptomatic | 1-3 years |
| Wang et al. 2019 (1) | 1-14 | N/A | N/A | N/A |
| Wang et al. 2019 (2) | 1-26 | N/A | Recovered | 0.5-5 years |
| Wang et al. 2019 (3) | 1 | N/A | Memory decline and psychiatric symptoms were remarkably recovered | 3-4 weeks |
|  | 2 | N/A | Cognitive impairment was partly recovered | 3-4 weeks |
|  | 3 | N/A | All symptoms were fully recovered | 3-4 weeks |
|  | 4 | N/A | Moderate improvement of cognitive impairment | 3-4 weeks |
|  | 5 | N/A | Mild improvement of gait disturbance | 3-4 weeks |
|  | 6 | N/A | Remarkable improvement of cognitive impairment | 3-4 weeks |
|  | 7 | N/A | Weakness of lower limbs was remarkably recovered | 3-4 weeks |
|  | 8 | N/A | Weakness of lower limbs was remarkably recovered | 3-4 weeks |
|  | 9 | N/A | Psychiatric symptoms were remarkably recovered | 3-4 weeks |
|  | 10 | N/A | Psychiatric symptoms were remarkably recovered | 3-4 weeks |
|  | 11 | N/A | Weakness of lower limbs was partly recovered | 3-4 weeks |
|  | 12 | N/A | Weakness of lower limbs was remarkably recovered | 3-4 weeks |
|  | 13 | N/A | Moderate improvement of vision and weakness | 3-4 weeks |
|  | 14 | N/A | Mild improvement of cognitive impairment | 3-4 weeks |
|  | 15 | N/A | Epilepsy and thrombosis were improved with antiepileptic and anticoagulant drugs | 3-4 weeks |
|  | 16 | N/A | Mild improvement of vision and depression | 3-4 weeks |
| Wang et al. 2019 (4) | 1 | N/A | Improvement | 4 weeks |
| Wei et al. 2019 | 1-8 | The levels of serum HCY and urine MMA were slightly decreased. | Improvement | 1 month |
| Wei et al. 2020 | 1 | N/A | N/A | N/A |
| Wen et al. 2020 | 1 | Decrease of urine MMA and plasma homocysteine. | Recovered | 3 months |
| Wu et al. 2017 | 1-2 | Decrease of urine MMA and plasma homocysteine. | Recovered | 6 months |
| Wu et al. 2018 | 1-3 | N/A | N/A | N/A |
| Zhao et al. 2021 | 1 | N/A | Improved | 6 months and 1 year |
|  |  |  | Recovered clinically and biochemically:64 patients  Improved: 78 patients  Did not improve: 4 patients  Asymptomatic: 11 patients  Died: 12 patients died  N/A: 30 patients | Mean follow-up: 31.87+43.48 months |
| **Abbreviations:** N/A: Not available | | | | |
